# A Systematic Topological Picture of ROS Scavenging Activity via Formal Hydrogen Atom Transfer in Phenols and Polyphenols

**DOI:** 10.1021/acs.joc.6c00415

**Published:** 2026-05-12

**Authors:** Davide Zeppilli, Matteo Filippi, Andrea Madabeni, Laura Orian

**Affiliations:** Dipartimento di Scienze Chimiche, Università di Padova, Via Marzolo 1, 35131 Padova, Italy

## Abstract

In this work, we employ a systematic quantitative computational approach to study the scavenging activity via formal hydrogen atom transfer (f-HAT) of a series of phenolic compounds. The most common features present in natural polyphenols are included to analyze their effect in the reactivity with radicals. All molecules are classified according to the analyzed effect: aromaticity size (class A), number and position of multiple hydroxyl (OH) groups (class B), and type and position of various substituents inspired by natural systems (classes C–E). The analysis also includes the corresponding anions for systems with p*K*_a_ values that permit deprotonation under physiological conditions. Finally, all the identified criteria for selecting effective polyphenolic antioxidants are validated in real systems, such as anthocyanidins, providing insightful tools for drug design.

## Graphical abstract

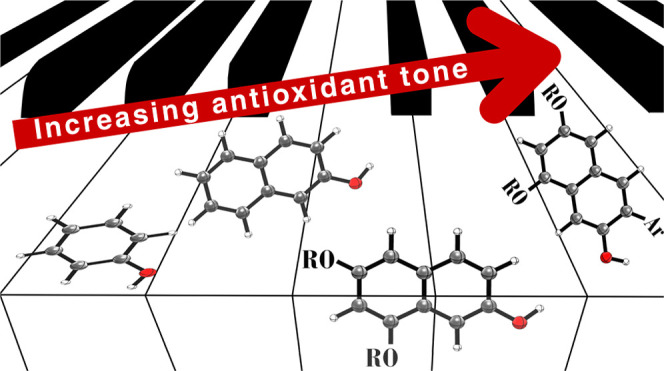

## 1 Introduction

The upkeep of the redox balance between pro-oxidant and antioxidant species is required for the proper functioning of cells, as an integral part of homeostasis. When this balance shifts uncontrollably toward pro-oxidants, the cells enter a state of oxidative stress,^1^ leading to damage in phospholipids, nucleic acids, and proteins.^2^ This condition is associated with diseases like neurodegenerative disorders (e.g., Parkinson’s, Alzheimer’s), mood disorders, schizophrenia, cardiovascular diseases, rheumatoid arthritis, diabetes, and certain cancers.^3–12^ However, it remains unclear whether oxidative stress is a cause or a symptom of these pathologies. Free radicals, part of the broader group of reactive oxygen and nitrogen species (ROS/RNS), are often blamed for their harmful effects but they are naturally produced by many biological processes,^13^ being essential for redox signaling.^14,15^ Nevertheless, excessive accumulation of these highly reactive species can disrupt cellular functions and cause significant damage.^16^ Therefore, this delicate cellular balance must be preserved. To this purpose, biological systems have developed a series of endogenous antioxidant defenses to inhibit radical-driven oxidation,^17^ including enzymes like superoxide dismutase (SOD),^18^ glutathione peroxidase (GPx),^19,20^ and catalase,^21^ as well as molecular systems like glutathione (GSH)^22^ and melatonin.^23,24^ When endogenous mechanisms falter, external antioxidants may be needed to counteract oxidative stress. These exogenous antioxidants, obtained through diet, act as scavengers by quenching free radicals. Historically, natural compounds were used to treat conditions later identified as related to oxidative stress, while modern antioxidant drugs have been developed for the same purpose.^25^

Among exogenous antioxidants, polyphenols are well-studied for their potent radical-scavenging and anti-inflammatory properties.^26–29^ Polyphenols derived from plants, fruits, vegetables and nuts, are classified^30–32^ into phenolic acids, flavonoids, and nonflavonoids like stilbenes (e.g., resveratrol)^33^ and lignans.^34^ Phenolic acids include hydroxycinnamic acids (e.g., *p*-coumaric acid and caffeic acid)^35^ and hydrobenzoic acids (e.g., gallic acid);^36^ while flavonoids, like anthocyanins (cyanidins) and flavonols (quercetin), represent the majority and are present in food like chocolate, tea, and wine.^37–39^

Polyphenols are extensively studied due to their ability to contrast oxidative stress.^40^ Studies reveal that their antioxidant activity can reduce inflammation, inhibit tumor progression, and support cardiovascular health.^41^ Despite their benefits, polyphenols can also exhibit pro-oxidant activity in the presence of transition metals, potentially propagating free radical reactions if unstable radical products react with oxygen. This dual nature underscores the need for balanced intake in health contexts.^42,43^

Exploring the mechanisms and energetics of the chemical reactions underlying antioxidant action is key to evaluating the ROS scavenging potential of substances used as supplements or drugs. Computational methods, particularly quantum chemical calculations, offer an efficient way to analyze the thermodynamics and kinetics of these reactions. Indeed, in silico approaches have been successfully employed for the investigation of radical scavenging properties of various compounds, including food components and pharmaceuticals.^44–49^ However, most of these studies are focused on a single (class of) molecules of interest; conversely, systematic analyses that allow for the identification of significant and recurring molecular topologies are less common. A comprehensive approach helps in exploring the modulation of molecular topologies to provide quantitative information about structure–activity relationships as well as their interpretation. This has an immediate impact on determining the antioxidant power of organic molecules and on the design of new scavengers. To the best of our knowledge, this approach has not been pursued for polyphenols so far.

Thus, in this work, we employ a systematic bottom-up approach to evaluate and rationalize the scavenging potential of important molecular motifs recurrent in natural antioxidants by employing several simple molecular models of polyphenols and phenolic compounds. These models were built to reproduce different effects, like the role of aromaticity extension, position and type of substituents. These chemical modulations take inspiration from well-known molecular classes, e.g. flavonoids, but also specific molecules such as caffeic acid, gallic acid, resveratrol and curcumin.^50^ The ROS scavenging activity of these species was tested focusing on hydrogen atom abstraction mechanism.

In general, radical scavenging may occur through different mechanisms, depending on both molecular and environmental properties.^51^ More specifically, in polar media at physiological pH, sequential proton loss electron transfer (SPLET) is the most relevant mechanism for many polyphenols, like flavonoids and phenolic acids.^51–53^ Indeed, they may deprotonate and engage in subsequent electron transfer reactions (Scheme 1). Conversely, for neutral phenolic molecules, the key mechanism is formal hydrogen atom transfer (f-HAT), which involve the transfer of a proton and an electron from a donor to an acceptor in a single step (Scheme 1).^43,54^ However, from a thermodynamic point of view, the two reactions are not distinguishable.

**Scheme 1:**
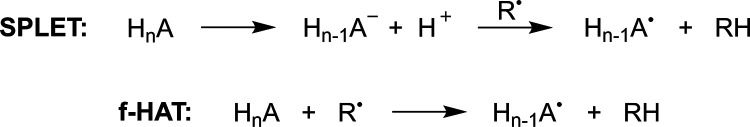
Sequential Proton Loss Electron Transfer (SPLET) and Formal Hydrogen Atom Transfer (f-HAT) for a Generic Antioxidant H_*n*_A and Free Radical R^•^

The term f-HAT is generally used when focusing on the thermodynamics of such processes, since a suitable kinetic description may discriminate between the proper hydrogen atom transfer (HAT) and concerted proton electron transfer (CPET). Specifically, HAT is found if the proton and the electron transfers can be described as a single process, i.e., they move together as a hydrogen atom. Conversely, the mechanism is CPET if the proton and the electron are transferred separately, often involving different molecular moieties for each individual process.^55–59^ Differentiating these mechanisms requires the analysis of the electron flow throughout the reaction pathway; to this purpose, nonempirical localized orbitals, such as IBOs (Intrinsic Bond Orbitals),^55,60,61^ are insightful, allowing a quantum-mechanically accurate but still chemically intuitive description.

In this work, f-HAT thermodynamics was evaluated for all compounds, while kinetic analysis and mechanistic details are reported just for few selected reactions. It is worth to point out that f-HAT reactions are usually well-behaved, and follow a Hammond behavior, i.e., the more negative the reaction energy, the lower the activation energy associated with the processes. Thus, kinetic considerations can be drawn with confidence even from a thermodynamic analysis. Indeed, the focus of the present work is to systematically check how different molecular topologies affect the thermodynamics of f‑HAT. Noteworthy, thermodynamic considerations are also valid for SPLET; indeed, the overall energetics of these two mechanisms are identical, since the same processes occur with just a different number of sequential steps (one or two, respectively). Particularly, five different ROSs are considered for each reaction: ^•^OH, ^•^OCH_3_, ^•^OOH, ^•^OOCH_3_, and ^•^OOCH=CH_2_. The hydroxyl radical (^•^OH) is the most reactive and electrophilic radical, capable of damaging macromolecules through near diffusion-limited reactions, followed by the methoxy radical (^•^OCH_3_).^62,63^ Conversely, hydroperoxyl radicals are less reactive and stable enough to diffuse within physiological environments.^64,65^ ^•^OOH and ^•^OOCH_3_ were selected to evaluate their reactivity against the corresponding alkoxyl radicals, while ^•^OOCH=CH_2_ serves as a simplified model for lipid hydroperoxyl radicals.

## 2 Computational Methods

Initial molecular geometries were manually constructed using the Chemcraft molecule builder.^66^ The XYZ coordinates were then used in input for full geometry optimization. All calculations were carried out with the Gaussian16 software package.^67^ Any multiple conformations were sampled manually, since the molecules are small and mostly rigid, and the geometry with the lowest energy was selected. M06-2X functional^68^ was used for geometry optimizations, combined with the 6–31G­(d) basis set. Frequency calculations were conducted at M06–2X/6–31G­(d) level of theory to obtain thermodynamic corrections to electronic energies, employing statistical mechanics formulas for ideal gases at 298 K and 1 atm.^69^ For the enthalpy correction of the H atom, only the translational motion was considered. The standard-state conversion from atmospheric pressure to 1 M concentration was considered in the calculation of Gibbs free energies in solution.^69^ In particular, for the reactions in water involving the hydroxyl radical, the correction term for the H_2_O product included its concentration of 55.5 M. The nature of the stationary states was also assessed: all minima are associated only to real frequencies, while one imaginary frequency characterizes transition states and is associated with the vibrational mode leading from the reactants to the products. Single point energy calculations were carried out to obtain more accurate estimates of the electronic energies, using the same functional combined with the extended 6–311+G­(d,p) basis set. In addition, two condensed phases were considered, i.e., water and benzene, by employing the continuum SMD model^70^ to simulate polar (water) and nonpolar (benzene) environments, respectively. This protocol (level of theory: (SMD)- M06–2X/6–311+G­(d,p)//M06–2X/6–31G­(d)) was validated and successfully applied to analyze the scavenging potential of small organic molecules, including minimal models of polyphenols.^44–46,71–74^ If not differently specified, the results discussed in the main text are computed in water (as a general and well-parametrized case of polar environment) and, in these cases, the corresponding results in the gas phase and benzene are qualitatively analogous.

Reaction paths were calculated using the intrinsic reaction coordinate (IRC) method.^75^ The intrinsic bond orbitals (IBOs)^60,61^ were computed and inspected to analyze the electron flow along the reaction path, as implemented in the IboView software. Since this software does not support the 6–31G basis set family, single point energy calculations were performed using using def2TZVP basis set. Electron spin density surfaces were drawn with VMD,^76^ with an isodensity value of 0.003 a.u. Spin contamination was checked for all open shell species (doublets) and was found to be negligible.

## 3 Results and Discussion

A wide series of phenolic compounds are analyzed to provide a systematic topological perspective over the effect of different structural features on the f-HAT reactivity of model phenolic compounds. In detail, we evaluate the effect of the aromatic moiety’s extension, as well as the chemical nature and position of several substituents, taking inspiration form natural antioxidants (e.g., curcumin, caffeic acids, gallic acid, resveratrol, flavonoids). All molecules are conveniently sorted into classes based on the structural modifications starting from the simple phenol. In particular, class A represents polycyclic monophenols and class B encompasses simple models of polyphenols; para-substituted phenols belong to class C, while meta-substituted phenols fall into class D. Finally, class E is formed by the ortho-substituted phenols. The same substituents are considered in the compounds of classes C, D and E to consistently evaluate the effect of the different position in the ring.

### 3.1 Class A

The extent of spin-delocalization is recognized to affect the stability of radical products in f-HAT reactions, i.e., the more delocalized the spin density in the radical product, the more favored the HAT thermodynamics. Thus, the first interesting question we wish to address is to what extent the size of the aromatic system can tune the stability of the phenoxyl radical to the point of influencing the selectivity of these systems toward different radicals. To this purpose, we have designed eight model monophenolic polyaromatic scaffolds spanning one to four condensed benzene rings (Class A, Figure 1). Each of these compounds may undergo f-HAT from the hydroxyl group; hence, a unique radical product is obtained from each reaction. Thermodynamic results of the radical scavenging of five different ROSs via f-HAT are also reported in Figure 1 for class A molecules in water (see Figure S1 and Table S1 for results in the gas phase and benzene).

**Figure 1:**
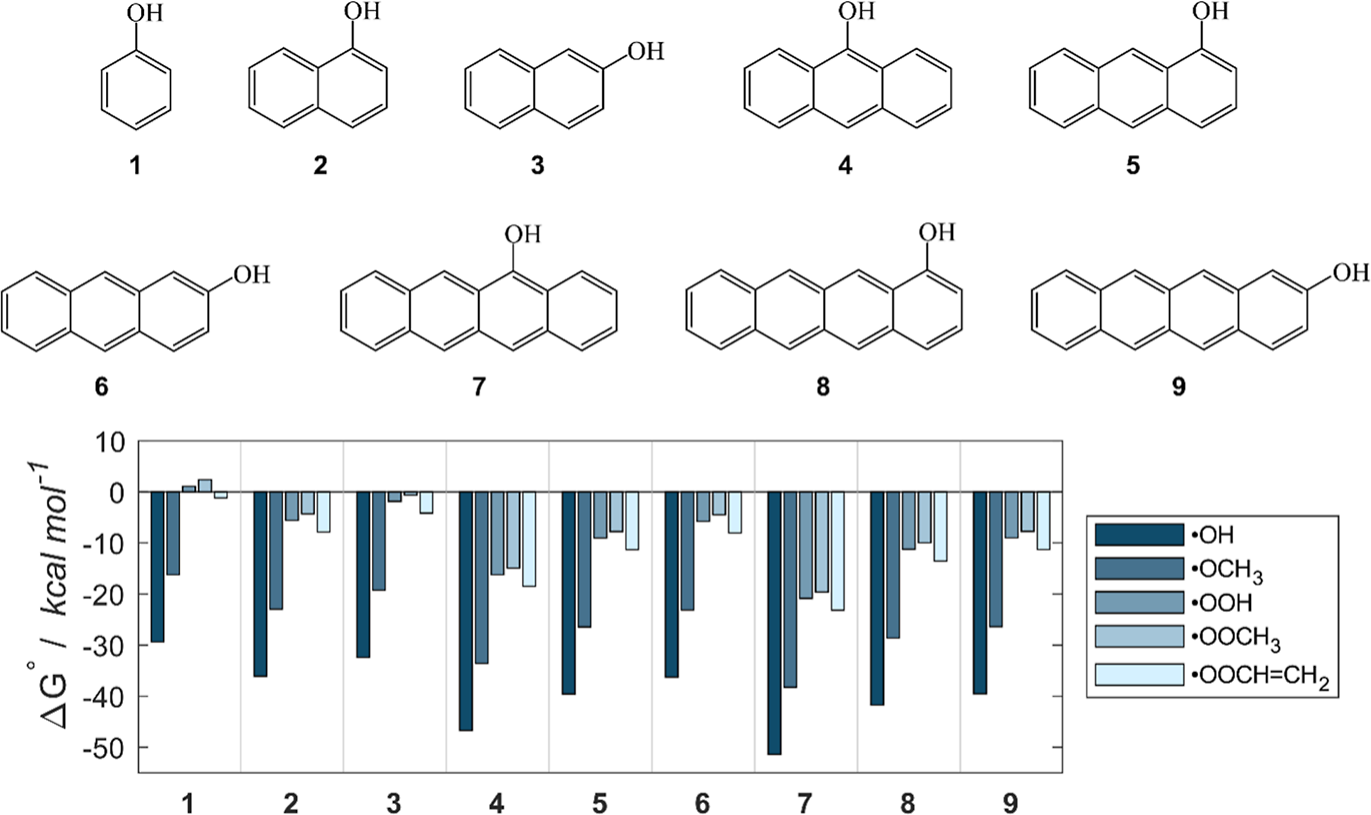
Class A compounds, which are aromatic polycyclic derivatives of phenol (**1**), and Gibbs free reaction energies (kcal mol^–1^) computed in water for the scavenging of ^•^OH, ^•^OCH_3_, ^•^OOH, ^•^OOCH_3_ and ^•^OOCH=CH_2_ via f-HAT from the OH site of **1**–**9**. Level of theory: SMD-M06–2X/6–311+G­(d,p)//M06–2X/6–31G­(d).

Expectedly, all compounds react very exergonically with hydroxyl and alkoxyl radicals. However, **1** (i.e., phenol) is not selective for ^•^OOH and ^•^OOCH_3_, and the reactivity with ^•^OOCH=CH_2_ is almost thermoneutral. As per our hypothesis, by extending the π system, all reactions become more and more exergonic, regardless of the ROS. This outcome is the result of the increased delocalization of the unpaired electron in the product over a greater number of carbon atoms. Notably, moving from phenol (**1**) to naphatalen-1-ol (**2**) is enough to impart to the molecular scaffold a (hydro, alkyl and alkenyl) peroxyl scavenging capacity. Even ^•^OOCH_3,_ the least reactive radical among those investigated, reacts with **2** with a Δ*G*° of ca. −4 kcal mol^–1^. Interestingly, the progressive addition of aromatic rings is only of limited advantage, as relative variations in Δ*G*° values decrease in absolute magnitude with increasing scaffold size. For instance, when moving from **2** to **5**, the energy gain is 3.5 kcal mol^–1^, while it decreases to 2.1 kcal mol^–1^ by proceeding from **5** to **8**, regardless of the ROS. Indeed, the radical product stability is progressively less influenced by any modification of the carbon skeleton further and further from the reaction center, as shown also by the spin densities of Figure 2.

**Figure 2:**
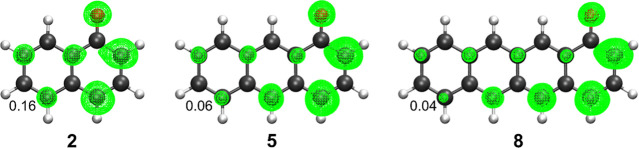
Spin densities of the radical products of f-HAT from the OH site of **2**, **5** and **8**. Isosurface value: 0.003 a.u. Spin density values of the furthest carbon atom are also reported. Level of theory: M06–2X/6–311+G­(d,p)//M06–2X/6–31G­(d).

The position of the OH group also plays a significant role; indeed, when comparing compounds with OH in pseudo α position (**2**, **5** and **8**) vs species with the hydroxyl group in pseudo β position (**3**, **6** and **9**), the former are characterized by more favorable f-HAT. Consequently, Δ*G*° values computed for **6** and **9** are comparable to those of **2** and **5**, respectively, although in these molecules a different number of condensed aromatic rings is present.

Indeed, pseudo α substituted compounds better stabilize the presence of an unpaired electron than the pseudo β analogues. Comparing the spin densities (Figure S2), a higher degree of spin delocalization is observed in **2** rather than in **3**. Indeed, the most negative Δ*G*° values are computed for **7** and **4**, which are able to delocalize the spin density in multiple rings close to the OH site. Particularly, by observing the spin density distributions (Figure S3), the unpaired electron is efficiently delocalized over nine and seven carbon atoms in the f-HAT product of **7** and **4**, respectively.

### 3.2 Class B

Polyphenol antioxidants are characterized by the presence of multiple hydroxyl functions on the aromatic building blocks of their scaffolds. While multiple OH groups can increase the radical scavenging potential for statistical reasons, they also affect the electronic structure of the title compounds, thus influencing the intrinsic thermodynamic stability of the radical product. To quantify this effect, all possible polyphenols accessible to one benzene ring are considered (class B), and the f-HAT thermodynamics is analyzed to evaluate the best positions for OH group enhancing the radical scavenging potential (Figure 3; see Figure S4 for results in the gas phase and benzene). The penta-substituted system has been neglected because there is no significant improvement in the presence of a full substituted ring (**19**). For asymmetric systems, distinct f-HAT sites are labeled with a lower-case letter (a, b, c).

**Figure 3:**
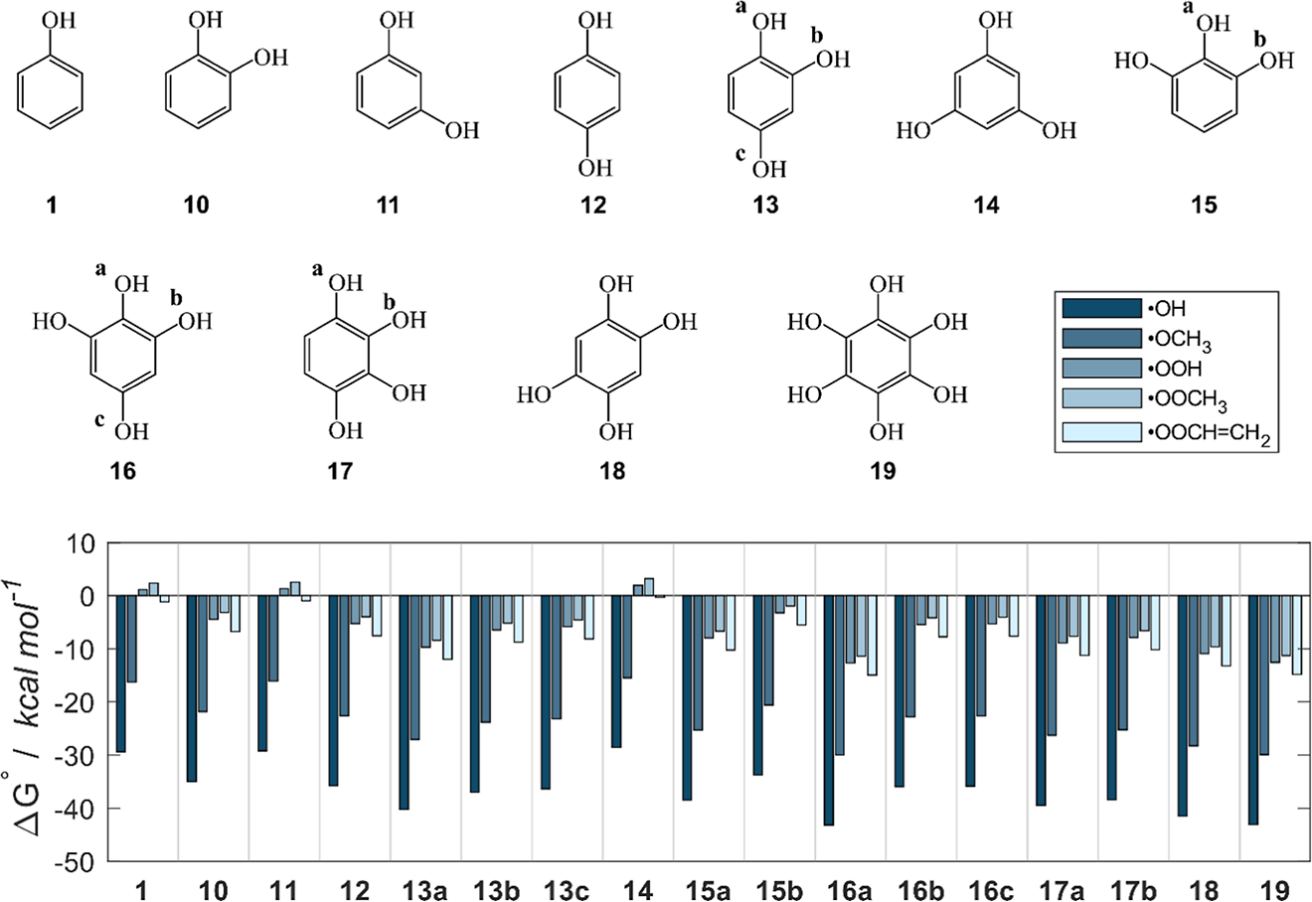
Class B compounds, which are simple models of polyphenols, and Gibbs free reaction energies (kcal mol^–1^) computed in water for the scavenging of ^•^OH, ^•^OCH_3_, ^•^OOH, ^•^OOCH_3_ and ^•^OOCH=CH_2_ via f-HAT from the OH sites of **1**, **10**–**19**. Level of theory: SMD-M06–2X/6–311+G­(d,p)//M06–2X/6–31G­(d).

Class B compounds exhibit an exergonic reactivity with all tested radicals, except **11** and **14**, i.e., the (doubly) meta-substituted species, whose reactivity is similar to that of the unsubstituted phenol **1**. For straight comparison, Δ*G*° values relative to the quenching of the ^•^OOH radical (i.e., one of the least reactive radicals) in water are reported in Table 1 in descending order of reactivity.

**Table 1:**
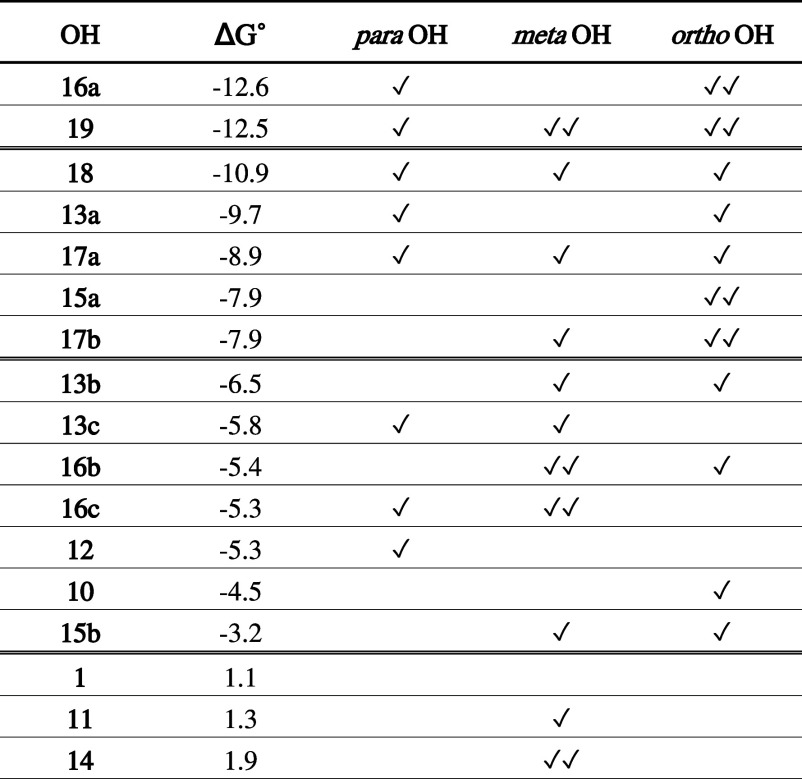
Gibbs Free Reaction Energies (kcal mol^–1^) Computed in Water for the Scavenging of ^•^OOH via f-HAT from the OH Sites of **1**, **10**–**19** (Class B Compounds)^a^

a Relative position of additional OH substituents are highlighted with respect to the reactive OH site. Level of theory: SMD-M06-2X/6-311+G­(d,p)//M06-2X/6-31G­(d).

Notably, monosubstitution of the phenol ring is enough to shift the reactivity toward peroxyl radicals from endergonic to exergonic. Indeed, the (ortho-substituted) catechol (**10**) and (para-substituted) quinol (**12**) already display moderately negative Δ*G*° (−4.5 and −5.3 kcal mol^–1^ respectively) in presence of ^•^OOH. Conversely, addition of either one (**11**) or two (**14**) OH groups in the meta position of phenol does not significantly influence the energetics. This observation suggests that only positions which can allow a significant spin delocalization are effective in tuning the stability of the final radicals (Figure S5). Conversely, electron donation via an induction mechanism (which is active even in meta position) has little to no effect on the stability of the radical product. This is supported by the negligible effect of halogen substitution in H abstraction, for instance, since their electron withdrawing inductive effects are offset by their weaker donating properties.^77,78^

Thus, it does not come unexpected that the most favored H abstraction sites are those in which all para and ortho positions to the H abstraction site are functionalized with additional OH groups, i.e., **16a** or any site of **19**. Indeed, moving from **12**, in which both ortho positions are nonfunctionalized, to **13a** (one ortho position is vacant), and to **16a** (no ortho or para positions vacant), Δ*G*° in presence of ^•^OOH becomes significantly more negative from −5.3 to −9.7 and to −12.6 kcal mol^–1^, respectively. The lack of influence of meta substituents is further highlighted by the virtually indistinguishable reactivities of **16a** and **19**, which differ only for two additional hydroxyl groups in meta position in the latter; indeed, no appreciable difference in the spin densities of their radical products is computed (Figure S5).

While f-HAT sites with one unsubstituted ortho or para position are characterized by Δ*G*° of ca. 2–4.5 kcal mol^–1^ less negative than **16a** and **19**, they can react with ^•^OOH with Δ*G*° of ca. −8 or lower. Moreover, **15a** and **17b** benefit from the presence of intramolecular hydrogen bonds that stabilize the radical oxygen, although this is not sufficient to reach the stabilization effects of para substituents in water. However, this picture changes in the gas phase or in the presence of low polar solvents, like in benzene (Table S2). In these environments, the hydrogen bonds are more impactful on the radical stability; therefore, the reactivity of **15a** and **17b** sites is enhanced, and comparable to simultaneously para- and mono ortho-substituted sites (**13a**, **17a** and **18**) with Δ*G*° of around −10 kcal mol^–1^.

Δ*G*° values in the range between −3 and −6 kcal mol^–1^ are computed for compounds with either para- or mono ortho-substituted sites, while the presence of groups in meta position has no significant effect. However, a Δ*G*° difference of 3.3 kcal mol^–1^ (2.2 kcal mol^–1^ in the gas phase) is computed for the H abstractions from **13b** and **15b**, which are isomers. This exception can be ascribed to the presence of a OH group in meta position with respect to the radical oxygen product but, at the same time, the same group can be seen as in para position with respect to the H bond donor hydroxyl group. The H bond increases the donor’s radical character, allowing a small spin delocalization on the OH group in meta position with respect to the reaction center. As shown in Figure 4, the spin density of the product of **13b** is slightly delocalized on a OH group unlike in the product of **15b**. Moreover, if the intramolecular H bond is removed in the former by a simple rotation of the hydroxyl group, the delocalization on the additional OH group is prevented, and a reduced reactivity is predicted.

**Figure 4:**
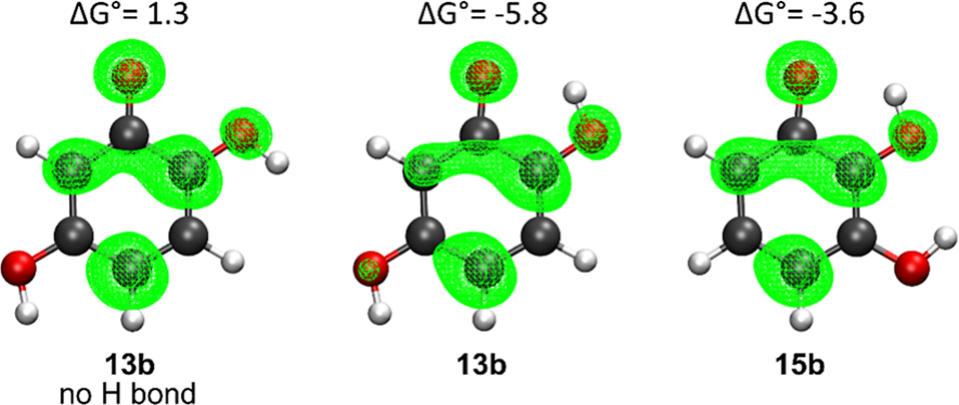
Spin densities of the radical products of f-HAT from the OH site **13b** (with and without the intramolecular H bond) and **15b**. Isosurface value: 0.003 a.u. Gibbs free reaction energies computed for the scavenging of ^•^OOH in the gas phase are also shown. Level of theory: M06–2X/6–311+G­(d,p)//M06–2X/6–31G­(d).

Noteworthy, H abstraction from **18** is more favorable than from **17a** for the same reason, since they only differ for the permutation of an OH group in meta position. Conversely, this spin delocalization in meta position induced by an H bond is not effective if two OH groups simultaneously create two H bonds with the same radical oxygen. No difference is indeed computed between **16a** and **19**, meaning that each H bond is less strong in these species, not allowing a delocalization on OH substituents in meta position.

In fact, the intramolecular H bond may have a double effect. Besides the stabilization of the radical product, a H bond may allow or facilitate an intramolecular f-HAT process, leading to a more stable final product, like the direct product of H abstraction from **13a** and **15a**. Such an intramolecular process is associated to a low activation barrier (7 kcal mol^–1^ for **13**), which is smaller or at least comparable to those of intermolecular f-HATs with free radicals (see the next paragraph).

### 3.3 Class C

The systematic analysis of class B molecules unravels the para position as the most effective for the reactivity of phenyl compounds via f-HAT, at least in water. Indeed, electronic effects are the sole contribution to the reactivity modulation of para-substituted compounds, while steric effects and H bonds may have an additional influence with substituents in ortho position. Therefore, para-substituted compounds are analyzed in class C to evaluate the effect of different groups on f-HAT. Besides the OH group, carboxyl groups are considered in ketones and carboxylic acids, as well as an olefinic pendant and their combinations. Figure 5 shows all the substituents included in class C, which are inspired by groups present in natural antioxidants; for example, **21** is found in the chemical scaffold of gallic acid, **23** and **24** are from curcumin and caffeic acid. Lastly, an additional aromatic ring is used as substituent, similarly to what can be found in honokiol and magnolol (**26**) or resveratrol (**27** and **28**).

**Figure 5:**
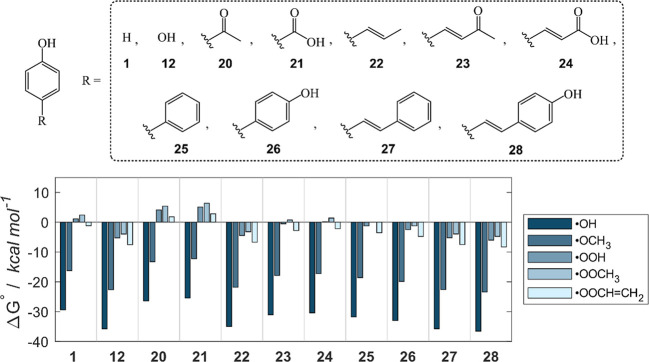
Class C compounds, which are para-substituted phenols, and Gibbs free reaction energies (kcal mol^–1^) computed in water for the scavenging of ^•^OH, ^•^OCH_3_, ^•^OOH, ^•^OOCH_3_ and ^•^OOCH=CH_2_ via f-HAT from the OH sites of **1**, **12**, **20**–**28**. Level of theory: SMD-M06–2X/6–311+G­(d,p)//M06–2X/6–31G­(d).

Figure 5 also shows the Gibbs free energies of f-HAT from the OH site of each compound of class C, while the corresponding data in the gas phase and benzene are in Figure S6. At first glance, any substituent leads to enhanced scavenging activity with respect to **1**. Exceptions are **20** and **21**, which are not selective for peroxyl radicals. Indeed, any other Δ*G*° value becomes more negative, but only slightly more exergonic reactions are found for **23** and **24**. The presence of a strongly polar medium does not change the energy trend, but thermodynamics is overall more favored. For instance, in benzene (Table S3), Δ*G*° are slightly more positive with differences of 1–2 kcal mol^–1^.

Focusing on the f-HAT in presence of ^•^OOH in water, it is possible to sort the substituents by the corresponding Δ*G*°; Figure 6 shows this reactivity order, from the most to the least favored reaction. The chosen substituents can be classified into three subgroups based on the computed Gibbs free f-HAT energy with respect to **1**: (i) more negative Δ*G*° as for **28**, **12**, **27**, **22**, **26**, and **25**; (ii) more positive Δ*G*° of **20** and **21**; (iii) comparable Δ*G*° (**23**, **24** and of course **1**).

**Figure 6:**
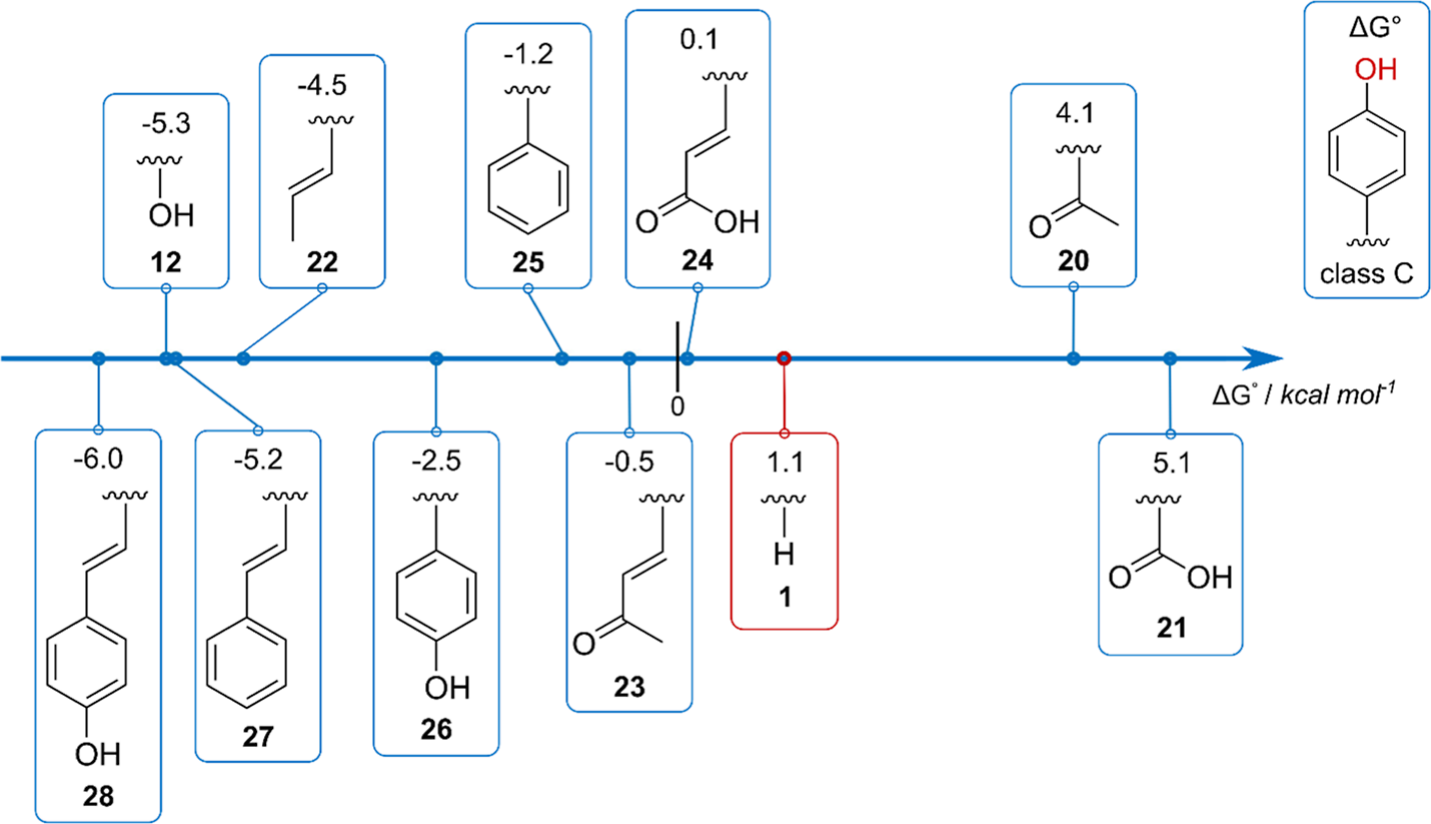
Para-substituted phenols of class C (**1**, **12**, **20**–**28**) are sorted by increasing Gibbs free reaction energies (kcal mol^–1^) computed in water for the scavenging of ^•^OOH via f-HAT from the OH site. Level of theory: SMD-M06–2X/6–311+G­(d,p)//M06–2X/6–31G­(d).

At first glance, it may seem that a substituent with a more extended π-system favors the reaction more significantly. However, this does not explain the enhanced reactivity of **12** or the comparable reactivity of **23** and **24** with respect to **1**. Furthermore, despite the possibility to delocalize the spin in an extended π-system, the reactions of **20** and **21** are much less favorable than when the conjugation is not extended at all (**1**). Therefore, it is worth referring to the electronic effects of the substituents. Indeed, the substituents of subgroup (i) are electron-donors, while those of subgroups (ii) are electron-withdrawing moieties.

The significant role of electron donors and acceptors in the scavenging activity was already hypothesized^79,80^ and proved in vitro;^81,82^ however, in this work, we quantified and rationalized these electronic effects in a systematic scale based on the reactivity of the selected compounds toward biologically relevant ROS models. In this sense, we wish to provide insights into the entity of these effects in enhancing the reactivity of phenols toward peroxyl radicals, unravelling which groups are more suitable than others.

Lastly, in **23** and **24** (subgroup (iii)), two vinylic carbons (weakly electron-donating) are interposed between the aromatic ring and the carbonyl group (electron-withdrawing). Consequently, Gibbs free reaction energies are not particularly different from Δ*G*° of **1**.

Due to the variability of Class C scavengers in thermodynamic terms (either more or less reactive than **1**), we have calculated the activation energies of f-HATs. Table 2 shows the Gibbs free activation energy for the quenching of ^•^OOH via f-HAT by all class C compounds in the gas phase, water and benzene. The same energy trend is observed, regardless of the environment. The smallest barriers are computed for compounds of subgroup (i), while the highest ones are computed for **20** and **21**. The media only modulate the heights; particularly, gas phase and benzene values are comparable, while Δ*G* ^⧧^ may increase (like **1**, **20** and **21**) or decrease (like **27** and **28**) by 1 or 2 kcal mol^–1^. Overall, the kinetic feasibility reflects the thermodynamic stability. This behavior can be interpreted in light of the Hammond postulate, according to which a more negative reaction energy is associated with a lower activation barrier. Analysis of the transition state structures for the f-HAT reactions (Figure 7) further supports this interpretation. In particular, the extent of O–H bond cleavage in the phenolic scaffold and the degree of O–H bond formation in the hydroperoxyl radical indicate that the transition state is early (reactant-like) when the reaction is exergonic (as in the case of **12**), whereas it is late (more product-like) when the reaction is endergonic (as in the case of **21**). The same trend is observed for all the other calculated transition states.

**Table 2:** Gibbs Free Activation Energies (kcal mol^–1^) Computed in the Gas Phase, Water and Benzene for the Scavenging of ^•^OOH via f-HAT from the OH Sites of **1**, **12**, **20**–**28** (Class C Compounds) in Decreasing Order of Reactivity^a^

	water	gas phase	benzene
28	12.3	14.0	13.3
12	13.7	14.0	13.4
27	13.9	14.8	14.2
22	14.0	14.7	14.1
26	16.0	15.8	15.2
25	16.8	15.9	15.5
23	18.3	16.7	16.5
24	18.7	17.2	17.1
1	18.9	17.4	17.1
20	21.7	18.8	18.8
21	22.3	19.2	19.3

a Level of theory: (SMD)-M06-2X/6-311+G­(d,p)//M06-2X/6-31G­(d).

**Figure 7:**
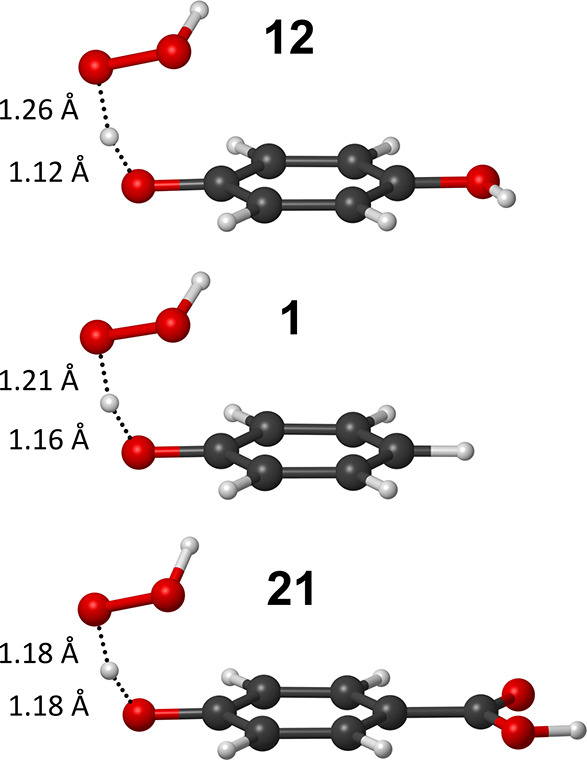
Fully optimized structures of selected transition states. Level of theory: M06–2X/6–31G­(d).

In the literature, the scavenging mechanism of phenolic compounds is associated with CPET rather than to a proper HAT, in which an electron is transferred directly form the π system of the aromatic ring.^44,83–87^ To assess whether this is true in the panel of compounds under investigation, we analyzed the electron flow along the reaction path by inspecting the spin IBOs involved in the reactivity. Particularly, it is interesting to see if the CPET is still active in electron depleted system; thus, a mechanism investigation is reported for **20**, as a reference case.

Figure 8 shows the change in the main α and β spin IBOs involved in the H abstraction from **20** by ^•^OOH, starting from the reactant, through the transition state, until the product. As the proton is progressively transferred from the hydroxyl group of **20** to the peroxyl radical, the electron transfer involves the π system of the aromatic ring, as shown by the blue spin IBO (β_π_ in Figure 8), which, initially localized on the ring, evolves in the direction of the phenolic oxygen and of the peroxyl moiety. Then, as the reaction proceeds, it remains localized only on the peroxyl moiety, as one spin orbital of the newly formed OH σ bond. Meanwhile, the corresponding α spin orbital (α_π_ purple IBO in Figure 8) does not change during the reaction and remains unpaired, causing the radical character of the product.

**Figure 8:**
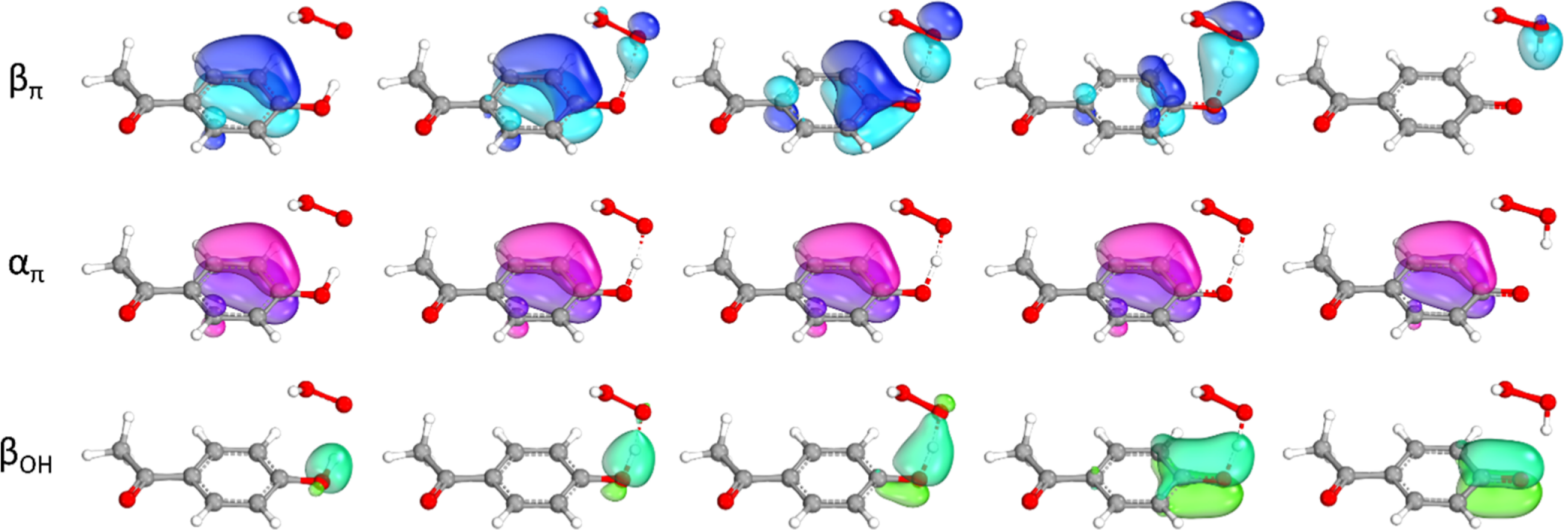
Changes in the main spin IBOs involved in the scavenging of ^•^OOH via f-HAT from the OH site of **20** along the reaction path: β_π_ spin IBO (blue) transferred from the π system of the ring to the peroxyl radical and the corresponding α_π_ spin IBO (purple), β_OH_ spin IBO of the OH σ bond (green). Level of theory: M06–2X/def2TZVP//M06–2X/6–31G­(d).

Lastly, the β spin orbital of the OH σ bond (β_OH_ green IBO in Figure 8) does not move together with the proton; indeed, it remains localized on the phenol as a π C=O bond orbital. The transfer of such electron would occur in the case of proper HAT. Therefore, since the transferred electron comes from a spin orbital which is not localized on the transferred proton, the reaction mechanism can be described as CPET.

Analogous electronic pictures are found in the case of **22** and **23** (Figures S7 and S8), similarly to the CPET description of the H abstraction from **1** previously reported by some of us.^83^ Hence, the f-HAT reactions reported so far should be rigorously described as CPETs, regardless of the electron donating/withdrawing groups. Moreover, electronic trends may be easily rationalized by the tendency of an aromatic electron to be transferred; electron rich compounds are more likely to lose one electron from the π system in a CPET process, being associated with the most exergonic reactions, as in class C compounds.

In the next paragraphs, H abstractions will be referred to as f-HAT for thermodynamic considerations; kinetic evaluations can be found in the Supporting Information for a representative of each class to confirm the reiteration of the CPET mechanism. Overall, as already reported, the CPET mechanism in phenolic compound depends on the orbital interaction between the involved molecules,^83,84^ and the OH reaction site allows a strong interaction to perform CPET. Changing the substituent does not prevent this interaction from occurring, but it may just modulate the reactivity, without switching the mechanism to HAT.

### 3.4 Class D and E

In paragraph 3.2, we reported that meta substitution has virtually no effect on the reactivity of polyphenols. To extend our discussion, the effect of group C substituents was also evaluated for meta-substituted compounds, i.e., class D (Figure 9); in Figure S9, results in the gas phase and benzene are shown. No significant difference is computed in the energetics within compounds of class D since Δ*G*° values are comparable, regardless of the substituent or the extension of the π system. Thus, the insertion of a substituent in the meta position does not enhance the reactivity of **1**. Conversely, only a small effect of 2 kcal mol^–1^ is found in the Δ*G*° of **29** and **30**, which are slightly less reactive than **1**. As for class C compounds, the environment does not affect this trend, but the reactivity is reduced in less polar media (Table S4).

**Figure 9:**
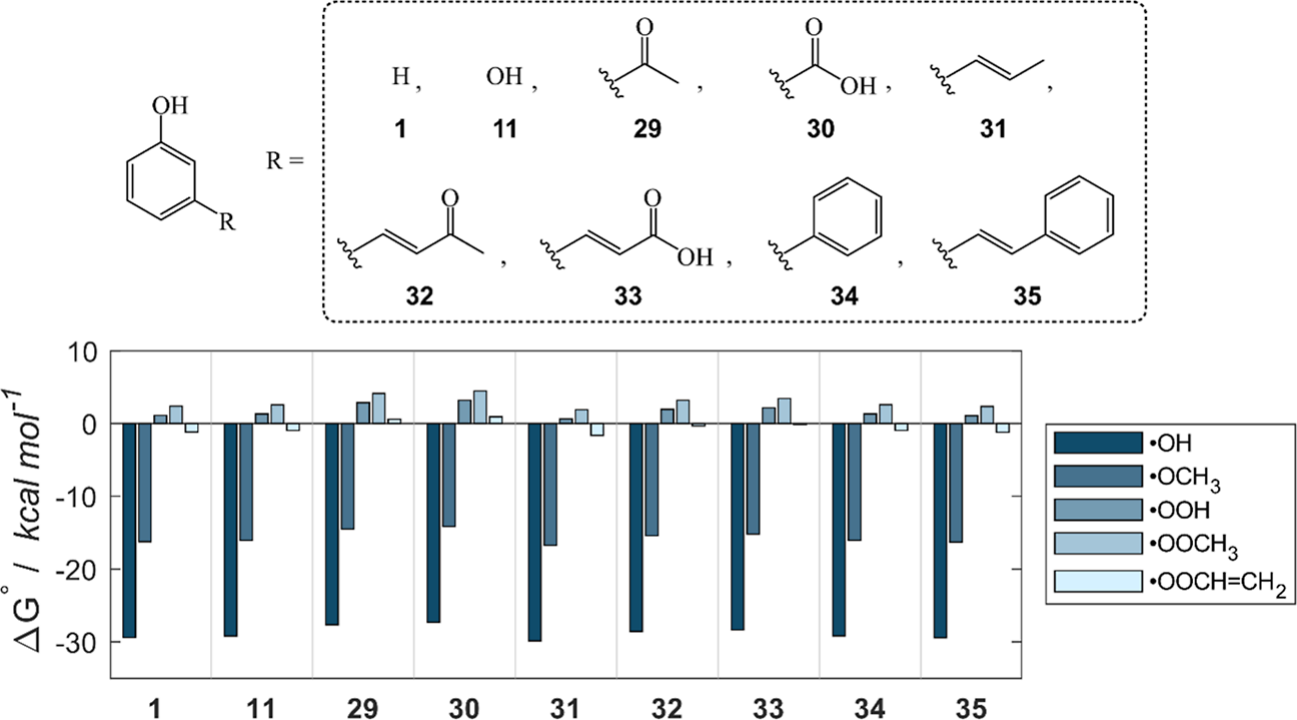
Class D compounds which are meta-substituted phenols, and Gibbs free reaction energies (kcal mol^–1^) computed in water for the scavenging of ^•^OH, ^•^OCH_3_, ^•^OOH, ^•^OOCH_3_ and ^•^OOCH=CH_2_ via f-HAT from the OH sites of **1**, **11**, **29**–**35**. Level of theory: SMD-M06–2X/6–311+G­(d,p)//M06–2X/6–31G­(d).

The presence of a substituent in meta position does not provide any additional stabilization, as can be observed by looking at the spin densities of few compounds of class D (Figure S10). In each case, the substituent is not involved in the delocalization of the spin, explaining why no significant difference is computed. For the same reason, the H abstraction site is neither enriched nor depleted of electron density by the presence of any substituent. However, the slight worsening of the energetics in **29** and **30** may be rationalized by the electron-withdrawing character of the substituent, which still lowers the reactivity of these compounds.

Overall, para-substituted compounds are more reactive than meta-substituted ones. Exceptions are those with carbonyl groups (**20** and **21** compared to **29** and **30**, respectively), since the meta-substituted species (**29** and **30**) are slightly more reactive. Indeed, in these cases, the meta-substitution does not allow to withdraw electron density from the phenolic oxygen (like in the corresponding para-substituted phenols **20** and **21**), making the radical less unstable. However, f-HATs for these systems are still less favorable compared to other compounds and phenol itself. Thus, the presence of electron-withdrawing substituents is not an advantage, but overall, it brings less disadvantages if they are inserted in meta position. To sum up, the lack of any reactivity trend in class D meta-substituted compounds highlights that f-HAT reactivity is ruled by electronic effects, while the inductive term has little to no importance, neither in withdrawing nor in donating electron density. Accordingly, the spin density is minimally affected by the nature of the meta-substituent (Figure S10).

Finally, ortho-substituted compounds were examined in class E, maintaining consistency with the previous classes regarding the added groups. Figure S11 and Table S5 show the studied compounds (**36**–**42**) and Gibbs free energies of f-HAT from the OH site of each molecule in the three different environments (water, gas phase and benzene). The substituent effect is ruled by electronic properties, as for para-substituted class C compounds. Electron-donor groups enhance the reactivity with respect to **1**, while electron-withdrawing groups lead to less negative Δ*G*°.

The electronic effects are reduced for ortho-substituted phenols if compared to the para-substituted one. This may also be ascribed to steric effects which may slightly change the energy trends. For instance, the carboxylic acid is slightly more reactive than the ketone due to the formation of an intramolecular H bond, which stabilizes the product. This cannot occur in the corresponding para-substituted compound, and the carboxyl acid was found to be less reactive than the ketone. However, carboxyl acids are likely to be deprotonated in physiological conditions; thus, no intramolecular H bond may occur.

We must highlight that Gibbs free energies are discussed, so far; however, Bond Dissociation Enthalpies (BDE) are standard descriptors in analogous studies. Thus, for the sake of completeness, BDEs are reported in Table 3. In general, the calculated O–H BDEs follow the same reactivity trend observed for the computed Gibbs free energies of the radical reactions, indicating that the thermodynamics discussed above is primarily driven by the intrinsic propensity of these substrates to undergo H atom transfer. For example, the trends related to ring size (**2** vs. **5** vs **8**) and to the pseudo-ortho/pseudopara arrangement (**2** vs. **3** or **5** vs. **6**) observed in class A; the higher favorability of reactions with ortho and para substituents in class B (**10**, **12** vs **11**); the different behavior of para substituents in class C (**21** vs **12**); and the negligible effect of meta substituents in class D (cf. **1**, **11**, **14** with **29–35**) are all consistently reproduced by the BDE trends. Therefore, BDEs and reaction Gibbs free energies provide the same chemical picture and lead to identical reactivity trends. However, the latter values are able to discriminate the scavenging potential against stable and biologically persistent hydroperoxyl radicals.

**Table 3:** O–H Bond Dissociation Enthalpies (kcal mol^–1^) Computed in the Gas Phase for the Phenol and Phenolic Model Systems of Classes A–D^a^

class A	BDE	class B	BDE	class C	BDE	class D	BDE
**1**	76.6	**13a**	63.8	**20**	78.3	**29**	79.2
**2**	70.1	**13b**	67.4	**21**	79.1	**30**	78.1
**3**	73.6	**13c**	70.0	**22**	72.1	**31**	76.4
**4**	58.3	**14**	78.3	**23**	74.4	**32**	77.6
**5**	66.8	**15a**	63.0	**24**	75.2	**33**	77.7
**6**	70.0	**15b**	69.6	**25**	74.6	**34**	76.8
**7**	53.1	**16a**	57.7	**26**	73.9	**35**	76.8
**8**	64.8	**16b**	66.6	**27**	71.8		
**9**	67.2	**16c**	70.8	**28**	71.1		
**10**	68.1	**17a**	64.1				
**11**	78.0	**17b**	63.4				
**12**	71.3	**18**	62.9				
		**19**	62.7				

a Level of theory: M06-2X/6-311+G­(d,p)//M06-2X/6-31G­(d).

### 3.5 Anions

Since phenolic acids have a p*K*_a_ of ca. 4, their corresponding anions are the most representative species under physiological conditions.^88^ Therefore, f-HAT may occur directly from the anionic form, and the effect of the carboxylate group is compared to the corresponding protonated species, **21**, **24**, **30**, **33**, **37** and **40** (Figure 10). It is worth to mention that f-HAT may not be the preferential mechanism of action of anionic systems in aqueous environments, where polyphenolics’ antioxidant mechanism usually shifts in favor of SET (thus, an overall SPLET mechanism, see Introduction). However, some phenolic species can still reduce radicals via f-HAT mechanism even in water.^89,90^ Thus, aiming to provide a systematic insight into the title reaction, the effect of deprotonation on f-HAT thermodynamics is presented. If, under physiological conditions, the mechanism does indeed shift toward SET, all reactions will be even more favored than our predictions (vide infra). Intuitively, negatively charged systems are more prone to donate one electron to the incoming radical, and we expect them to be more reactive than neutral species, as previously reported.^45,48,54^ Indeed, the negatively charged carboxylate works as an electron donating substituent which promotes f-HAT with an average advantage of ca. 2 kcal mol^–1^. This effect is lower in meta-substituted compounds (due to the absence of spin delocalization over the π system, as shown in class D) and in the presence of the olefinic bridge (electron donating and withdrawing capacities balance out, vide supra). A remarkable exception is **37**, for which f-HAT from the anion is disfavored by 3.5 kcal mol^–1^, likely due to a particularly high steric repulsion between the negative charge of the carboxylate and the phenoxyl radical. Moreover, no stabilization of the product via intramolecular H bond is now possible.

**Figure 10:**
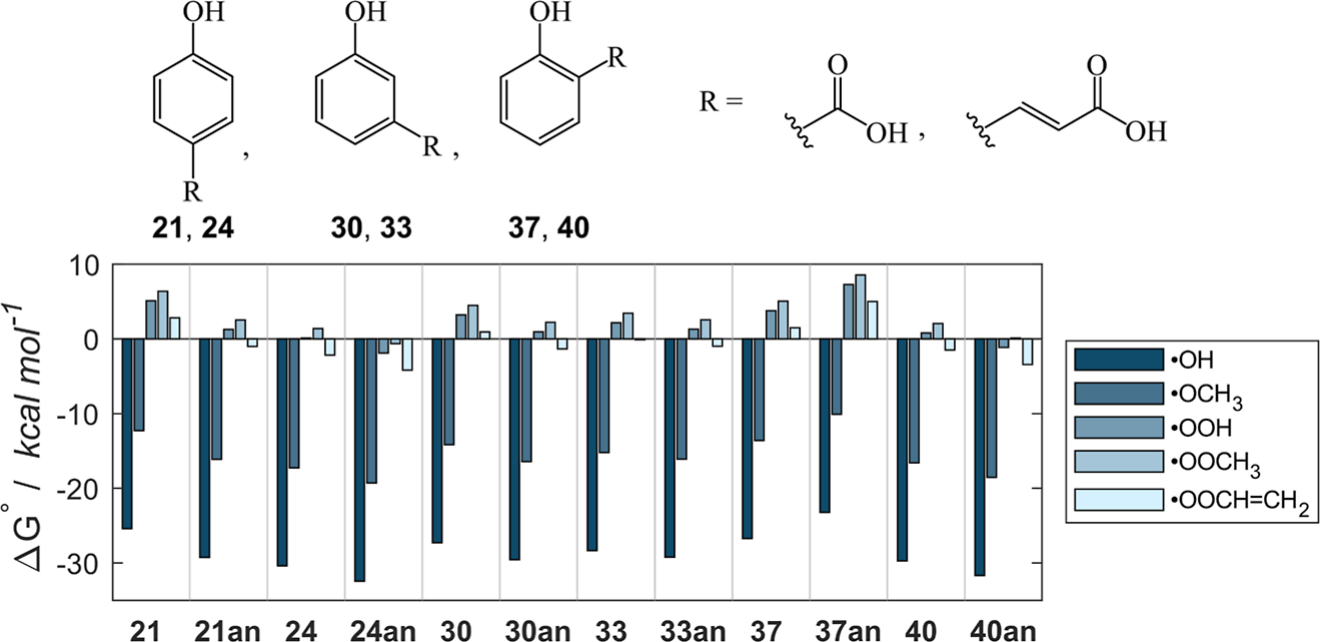
Gibbs free reaction energies (kcal mol^–1^) computed in water for the scavenging of ^•^OH, ^•^OCH_3_, ^•^OOH, ^•^OOCH_3_ and ^•^OOCH=CH_2_ via f-HAT from the OH sites of the phenolic acids **21**, **24**, **30**, **33**, **37**, **40** and their corresponding anions. Level of theory: SMD-M06–2X/6–311+G­(d,p)//M06–2X/6–31G­(d).

Overall, the inclusion of a carboxylate function improves the scavenging activity with respect to the unsubstituted phenol (**1**) in all cases, except when a direct COO^–^ is bonded in para (**21an**) or ortho (**37an**) position. In the former, the computed reactivity is comparable to **1** since the electron-withdrawing effect is offset by the negative charge, while in the latter the steric effects even worsen the reactivity.

Besides the carboxylic function, deprotonation may also affect the model polyphenols of class B. Indeed, when multiple OH groups are present the acidity increases, allowing possible deprotonation in physiological conditions. Indeed, moving from phenol (**1**) to catechol (**10**) and pyrogallol (**15**) the p*K*_a_ decreases from 10 to 9.3 and 9, respectively.^91,92^ The presence of a negatively charged oxygen does not change the previous considerations on the reactivity of class B compounds; however, the aromatic ring is further activated by the presence of the negative charge (**43**–**49**). Thus, Gibbs free energies of f-HAT in water are more favorable than those of the corresponding neutral compounds by an average of ca. 10 kcal mol^–1^ (Figure 11 and Table S6). Similar conclusions can be drawn from the BDE data (Table S7). Therefore, the deprotonation of an OH site has a stronger effect than the deprotonation of a carboxylic function. This effect may have a lower physiological impact since small scaffolds with several hydroxyl groups, such as **43**–**49**, are rare in nature; thus, the p*K*_a_ of real systems are higher and the deprotonation effect may be lower.

**Figure 11:**
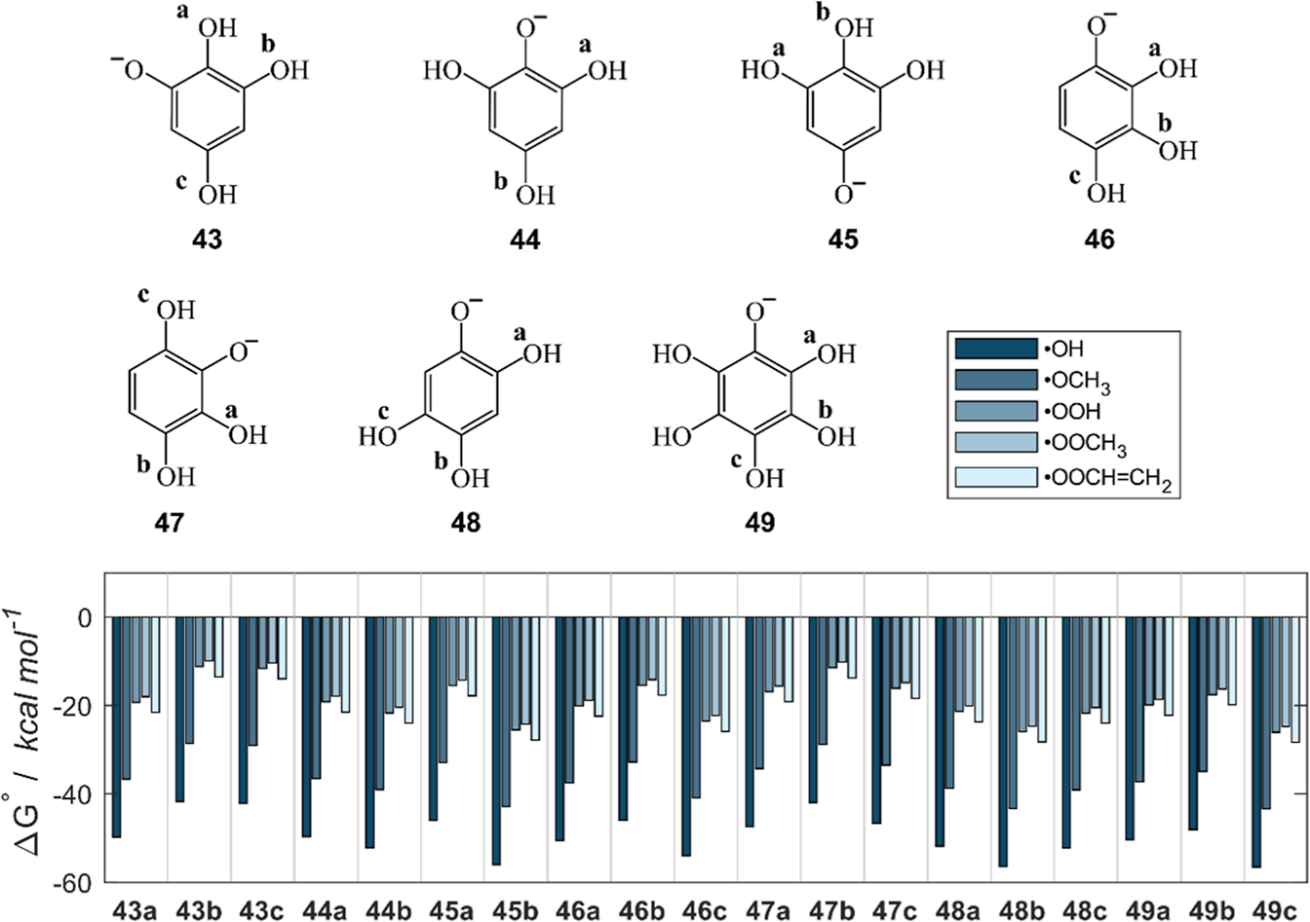
Chemical structure of the anions of class B compounds with four or more OH groups, and Gibbs free reaction energies (kcal mol^–1^) computed in water for the scavenging of ^•^OH, ^•^OCH_3_, ^•^OOH, ^•^OOCH_3_ and ^•^OOCH=CH_2_ via f-HAT from the OH sites of molecules **43**–**49**. Level of theory: SMD-M06–2X/6–311+G­(d,p)//M06–2X/6–31G­(d).

## 4 Conclusions

A systematic computational analysis is presented collecting the most important features for the scavenging activity of phenolic systems in a single work. Thus, different effects can be easily compared, such as the extension of the aromatic moiety, number, type and position of common substituents in model polyphenolic systems, and overall charge. A quantitative approach based on computing the chemical reactivity via f-HAT is used, in order to evaluate the actual effect of several properties on the quenching of free radicals. In particular, we aim to pinpoint the topological features which promote exergonic reactions with peroxyl radicals, which readily diffuse in biological environments due to their reduced reactivity when compared against hydroxyl radicals. Besides the systematic description, our analysis aims at providing rules for molecular screening and design to select an antioxidant with efficient scavenging potential by looking at the structure and optimizing it, if necessary. We can summarize the results in a “scavenging pentagram”, where the chemical features that contribute to enhancing the antioxidant power are represented as notes on an ascending scale (Figure 12).

**Figure 12:**
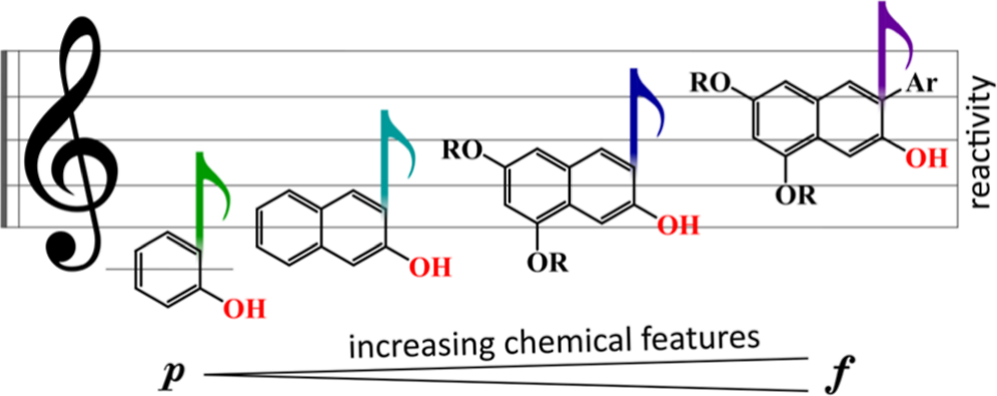
Scavenging potential of phenolic compounds via f-HAT is modulated by different factors, like the conjugation extension, number, type and position of substituents: according to the observed rules, the rational introduction of meaningful chemical features increases the reactivity, leading to efficient antioxidant activity.

As proof of the validity of our results, we selected a group of several anthocyanidins analyzing the chemical features responsible for their high reactivity toward free radicals. In this group, the most reactive site via f-HAT is always the same OH moiety, which exhibits similar properties in each case (Figure 13).^73,93^ It is a phenolic hydroxyl group belonging to an extended aromatic system formed by two condensed rings. An electron donor (Ar) is present in ortho position; particularly, it can be described as a substituted phenyl group with one or multiple OH and/or OMe groups. Furthermore, two additional electron donors (OR) are found in the second ring, enabling spin delocalization onto the oxygen atoms. Lastly, even though the pyrylium oxygen depauperates the ring of electron density, it is present in meta with respect to the reactive OH site, minimizing its unfavorable effect on f-HAT reactivity.

**Figure 13:**
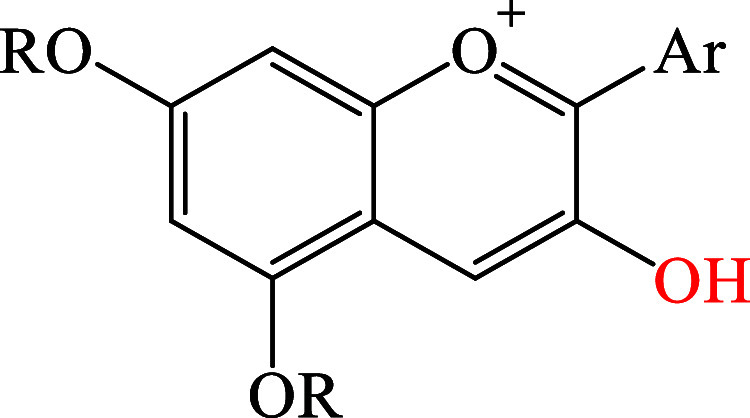
Chemical structures of a general anthocyanidin; the most reactive site is highlighted in red.

Thus, this example demonstrates the applicability of the results presented in this study about nature and position of substituents. In general, all the chemical features affect the antioxidant potential, but the presented chemically driven rules should be taken into account to rationally design new antioxidant drugs.

## Supplementary Material



## Data Availability

The data underlying this study are available in the published article and its Supporting Information.

## Notes

Gibbs free reaction energies (f-HAT) of each molecule; spin densities of selected radical products; IBOs for the f-HAT of selected reactions; structures and energies of class E compounds; BDE for class E and anions; coordinates, energies and imaginary frequencies of all optimized structures (PDF)
