# Supplementary material for: A Systematic Topological Picture of ROS Scavenging Activity via Formal Hydrogen Atom Transfer in Phenols and Polyphenols
Source: J Org Chem. 2026 May 12;91(20):6938–50. doi: 10.1021/acs.joc.6c00415 (PMC13200188; doi:10.1021/acs.joc.6c00415)
Supplement: Supplementary file 1 [file jo6c00415_si_001.pdf]

# – Supporting Information –

A Systematic Topological Picture of ROS Scavenging Activity via formal Hydrogen Atom Transfer in Phenols and Polyphenols

Davide Zeppilli [ǂ], Matteo Filippi [ǂ] Andrea Madabeni and Laura Orian [*]

Dipartimento di Scienze Chimiche, Università di Padova, Via Marzolo 1, 35131 Padova, Italy

[ǂ] These authors equally contributed to this study.

[*] Correspondence: laura.orian@unipd.it

LIST of TABLES and FIGURES:

Figure S1 Gibbs free reaction energies (kcal mol^{-1}) computed in gas phase (red) and benzene (green) for the scavenging of •OH, •OCH_3, •OOH, •$OOCH_3$ and •OOCH=CH_2 via f-HAT from the OH site of molecules **1**-**9** (class A). Level of theory: (SMD)-M06-2X/6-311+G(d,p)//M06-2X/6-31G(d)…..……………………………………………………………….S3

Figure S2 Spin densities of the radical products of f-HAT from the OH site of 2 and 3. Isosurface value of 0.003 a.u.. For both radicals, the reported resonance formulas show the possibility to delocalize the unpaired electron on one ring, maintaining the aromaticity on the other ring (indicated by a circle). Level of theory: M06-2X/6-311+G(d,p)// M06-2X/6-31G(d)……………………………………………………………………………………………..……S3

Figure S3 Spin densities of the radical products of f-HAT from the OH site of **4** and **7**. Isosurface value of 0.003 a.u.. Level of theory: M06-2X/6-311+G(d,p)//M06-2X/6-31G(d).…………………………………………………………S4

Table S1 Gibbs free reaction energies (ΔG°, kcal mol^{-1}) computed in the gas phase, water and benzene for the scavenging of •OH, •OCH_3, •OOH, •$OOCH_3$ and •OOCH=CH_2 via f-HAT from the OH site of molecules **1**-**9** (class A). Level of theory: (SMD)-M06-2X/6-311+G(d,p)//M06-2X/6-31G(d)………………….…………………………………………………….S4

Figure S4 Gibbs free reaction energies (kcal mol^{-1}) computed in gas phase (red) and benzene (green) for the scavenging of •OH, •OCH_3, •OOH, •$OOCH_3$ and •OOCH=CH_2 via f-HAT from the OH site of molecules **1**, **10**-**19** (class B). Level of theory: (SMD)-M06-2X/6-311+G(d,p)//M06-2X/6-31G(d)…………..…………………………………………………S5

Table S2 Gibbs free reaction energies (ΔG°, kcal mol^{-1}) computed in the gas phase, water and benzene for the scavenging of •OH, •OCH_3, •OOH, •$OOCH_3$ and •OOCH=CH_2 via f-HAT from the OH site of molecules **10**-**19** (class B). Level of theory: (SMD)-M06-2X/6-311+G(d,p)//M06-2X/6-31G(d)………………...…………………………………………S5

Figure S5 Spin densities of the radical products of f-HAT from the OH site of **16b** and **19**. Isosurface value of 0.003 a.u.. Level of theory: M06-2X/6-311+G(d,p)//M06-2X/6-31G(d)……….…………………………………………………S6

Figure S6 Gibbs free reaction energies (kcal mol^{-1}) computed in gas phase (red) and benzene (green) for the scavenging of •OH, •OCH_3, •OOH, •$OOCH_3$ and •OOCH=CH_2 via f-HAT from the OH site of molecules **1**, **12**, **20**-**28** (class C). Level of theory: (SMD)-M06-2X/6-311+G(d,p)//M06-2X/6-31G(d)……….....……………………………………………S6

Table S3 Gibbs free reaction energies (ΔG°, kcal mol^{-1}) computed in the gas phase, water and benzene for the scavenging of $^{\bullet}$OH, $^{\bullet}OCH_3$, $^{\bullet}$OOH, $^{\bullet}OOCH_3$ and $^{\bullet}OOCH=CH_2$ via f-HAT from the OH site of molecules **20-28** (class C). Level of theory: (SMD)-M06-2X/6-311+G(d,p)//M06-2X/6-31G(d)…………..…………………………………………………S7

Figure S7 Changes in the main spin IBOs involved in the scavenging of $^{\bullet}$OOH via f-HAT from the OH site of **22** along the reaction path: β_π spin IBO (blue) transferred from the π system of the ring to the peroxyl radical and the corresponding α_π spin IBO (purple), β_{OH} spin IBO of the OH σ bond (green). Level of theory: M06-2X/def2TZVP//M06-2X/6-31G(d)………S7

Figure S8 Changes in the main spin IBOs involved in the scavenging of $^{\bullet}$OOH via f-HAT from the OH site of **23** along the reaction path: β_π spin IBO (blue) transferred from the π system of the ring to the peroxyl radical and the corresponding α_π spin IBO (purple), β_{OH} spin IBO of the OH σ bond (green). Level of theory: M06-2X/def2TZVP//M06-2X/6-31G(d)………S8

Figure S9 Gibbs free reaction energies (kcal mol^{-1}) computed in gas phase (red) and benzene (green) for the scavenging of $^{\bullet}$OH, $^{\bullet}OCH_3$, $^{\bullet}$OOH, $^{\bullet}OOCH_3$ and $^{\bullet}OOCH=CH_2$ via f-HAT from the OH site of molecules **1**, **11**, **29-35** (class D). Level of theory: (SMD)-M06-2X/6-311+G(d,p)//M06-2X/6-31G(d)…………..……………………………………………S8

Table S4 Gibbs free reaction energies (ΔG°, kcal mol^{-1}) computed in the gas phase, water and benzene for the scavenging of $^{\bullet}$OH, $^{\bullet}OCH_3$, $^{\bullet}$OOH, $^{\bullet}OOCH_3$ and $^{\bullet}OOCH=CH_2$ via f-HAT from the OH site of molecules **29-35** (class D). Level of theory: (SMD)-M06-2X/6-311+G(d,p)//M06-2X/6-31G(d)……………..………………………………………………S9

Figure S10 Spin densities of the radical products of f-HAT from the OH site of **1**, **29** and **31**. Isosurface value of 0.003 a.u.. Level of theory: M06-2X/6-311+G(d,p)//M06-2X/6-31G(d)…….…………………………………………………S9

Table S5 Gibbs free reaction energies (ΔG°, kcal mol^{-1}) computed in the gas phase, water and benzene for the scavenging of $^{\bullet}$OH, $^{\bullet}OCH_3$, $^{\bullet}$OOH, $^{\bullet}OOCH_3$ and $^{\bullet}OOCH=CH_2$ via f-HAT from the OH site of molecules **36-42** (class E). Level of theory: (SMD)-M06-2X/6-311+G(d,p)//M06-2X/6-31G(d)……………..……………………………………………S9

Figure S11 Chemical structure of class E compounds, ortho-substituted phenols. Gibbs free reaction energies (kcal mol^{-1}) computed in water (blue), gas phase (red) and benzene (green) for the scavenging of $^{\bullet}$OH, $^{\bullet}OCH_3$, $^{\bullet}$OOH, $^{\bullet}OOCH_3$ and $^{\bullet}OOCH=CH_2$ via f-HAT from the OH sites of molecules **1**, **10**, **36-42** (class E). Level of theory: (SMD)-M06-2X/6-311+G(d,p)//M06-2X/6-31G(d)…………………………………………...…………………………..S10

Figure S12 Changes in the β_π spin IBO involved in the scavenging of $^{\bullet}$OOH via f-HAT from the OH site of **2**, **29** and **36** along the reaction path. Level of theory: M06-2X/def2TZVP//M06-2X/6-31G(d)….……………………………S11

Table S6 Gibbs free reaction energies (ΔG°, kcal mol^{-1}) computed in the gas phase, water and benzene for the scavenging of $^{\bullet}$OH, $^{\bullet}OCH_3$, $^{\bullet}$OOH, $^{\bullet}OOCH_3$ and $^{\bullet}OOCH=CH_2$ via f-HAT from the OH site of anions **21an**, **24an**, **33an**, **37an**, **40an**, **43-49**. Level of theory: (SMD)-M06-2X/6-311+G(d,p)//M06-2X/6-31G(d)…………………..………………………S12

Table S7 O-H Bond Dissociation Enthalpies (kcal mol^{-1}) computed in the gas phase for the phenolic model systems of classes E and anions. Level of theory: M06-2X/6-311+G(d,p)//M06-2X/6-31G(d)…………………….…………………...S13

Table S8 Coordinates (Å), electronic energies (E, Hartree) and Gibbs free energies (G, Hartree) of stationary points and imaginary frequencies (Nimag, cm^{-1}) of transition states. Level of theory: M06-2X/6-31G(d)…………………………S13

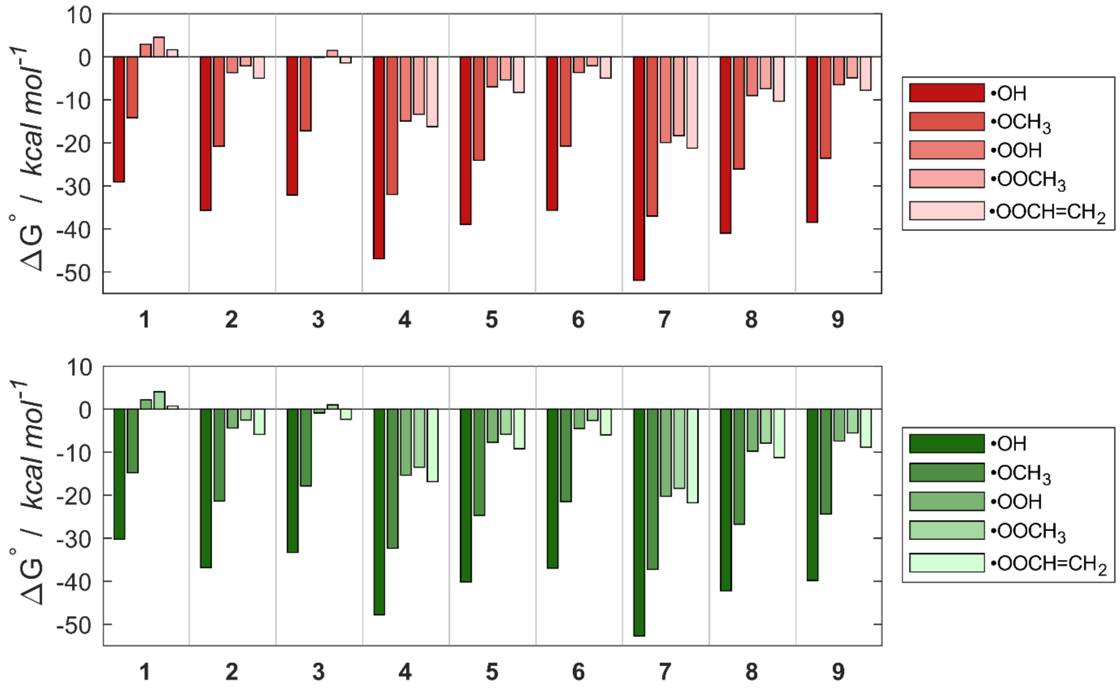

Figure S1 Gibbs free reaction energies (kcal mol^{-1}) computed in gas phase (red) and benzene (green) for the scavenging of $^{\bullet}$OH, $^{\bullet}OCH_3$, $^{\bullet}$OOH, $^{\bullet}OOCH_3$ and $^{\bullet}OOCH=CH_2$ via f-HAT from the OH site of molecules **1-9** (class A). Level of theory: (SMD)-M06-2X/6-311+G(d,p)//M06-2X/6-31G(d).

2

3

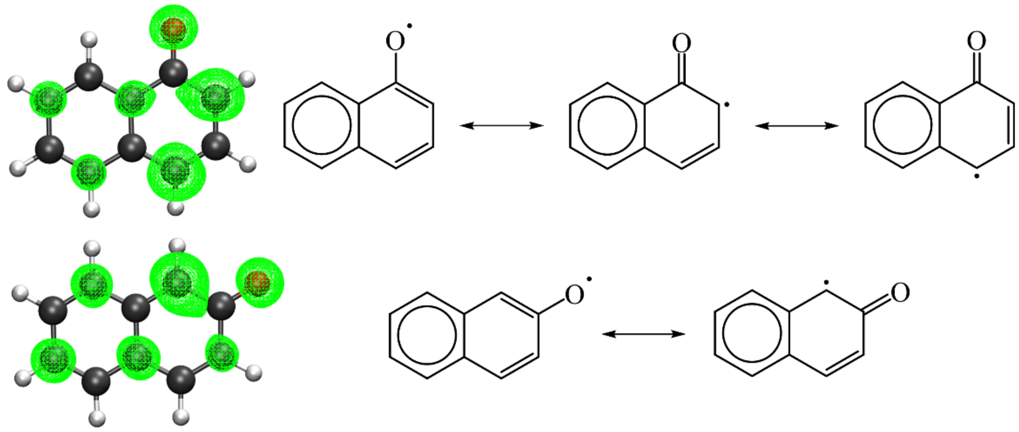

Figure S2 Spin densities of the radical products of f-HAT from the OH site of **2** and **3**. Isosurface value of 0.003 a.u.. For both radicals, the reported resonance formulas show the possibility to delocalize the unpaired electron on one ring, maintaining the aromaticity on the other ring (indicated by a circle). Level of theory: M06-2X/6-311+G(d,p)//M06-2X/6-31G(d).

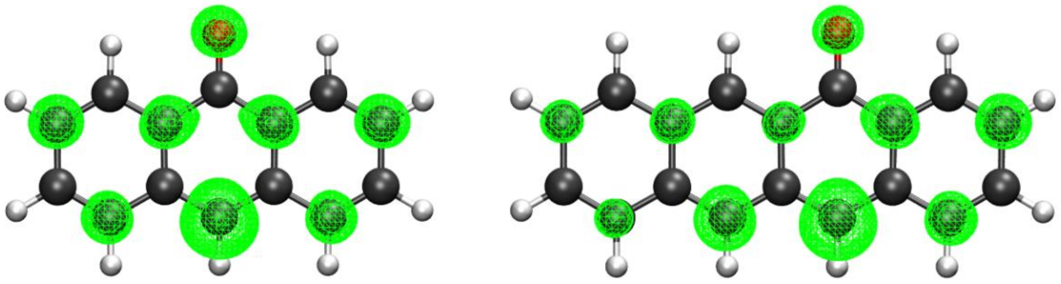

4 **7**

Figure S3 Spin densities of the radical products of f-HAT from the OH site of **4** and **7**. Isosurface value of 0.003 a.u.. Level of theory: M06-2X/6-311+G(d,p)//M06-2X/6-31G(d).

Table S1 Gibbs free reaction energies (ΔG°, kcal mol^{-1}) computed in the gas phase, water and benzene for the scavenging of $^{\bullet}$OH, $^{\bullet}OCH_3$, $^{\bullet}$OOH, $^{\bullet}OOCH_3$ and $^{\bullet}OOCH{=}CH_2$ via f-HAT from the OH site of molecules **1-9** (class A). Level of theory: (SMD)-M06-2X/6-311+G(d,p)//M06-2X/6-31G(d).

	**gas phase**					**water**					**benzene**				
	$^{\bullet}$OH	$^{\bullet}OCH_3$	$^{\bullet}$OOH	$^{\bullet}OOCH_3$	$^{\bullet}OOCH{=}CH_2$	$^{\bullet}$OH	$^{\bullet}OCH_3$	$^{\bullet}$OOH	$^{\bullet}OOCH_3$	$^{\bullet}OOCH{=}CH_2$	$^{\bullet}$OH	$^{\bullet}OCH_3$	$^{\bullet}$OOH	$^{\bullet}OOCH_3$	$^{\bullet}OOCH{=}CH_2$
**1**	-29.0	-14.1	3.0	4.5	1.7	-29.4	-16.3	1.1	2.4	-1.2	-30.2	-14.8	2.2	4.1	0.7
**2**	-35.7	-20.8	-3.7	-2.1	-5.0	-36.1	-23.0	-5.6	-4.3	-7.9	-36.8	-21.4	-4.4	-2.5	-5.9
**3**	-32.1	-17.2	-0.1	1.5	-1.4	-32.4	-19.2	-1.9	-0.6	-4.2	-33.3	-17.9	-0.9	1.0	-2.4
**4**	-46.9	-32.0	-14.9	-13.3	-16.2	-46.7	-33.6	-16.2	-14.9	-18.5	-47.8	-32.3	-15.4	-13.5	-16.8
**5**	-39.0	-24.1	-7.0	-5.4	-8.3	-39.6	-26.4	-9.1	-7.8	-11.3	-40.2	-24.7	-7.7	-5.8	-9.2
**6**	-35.7	-20.8	-3.7	-2.1	-5.0	-36.3	-23.1	-5.8	-4.5	-8.0	-37.0	-21.5	-4.5	-2.6	-6.0
**7**	-51.9	-37.0	-19.9	-18.3	-21.2	-51.4	-38.3	-20.9	-19.6	-23.2	-52.7	-37.3	-20.3	-18.4	-21.7
**8**	-41.0	-26.1	-9.0	-7.4	-10.3	-41.8	-28.6	-11.2	-10.0	-13.5	-42.2	-26.8	-9.8	-7.9	-11.2
**9**	-38.5	-23.6	-6.5	-4.9	-7.8	-39.5	-26.4	-9.0	-7.7	-11.3	-39.8	-24.4	-7.4	-5.5	-8.9

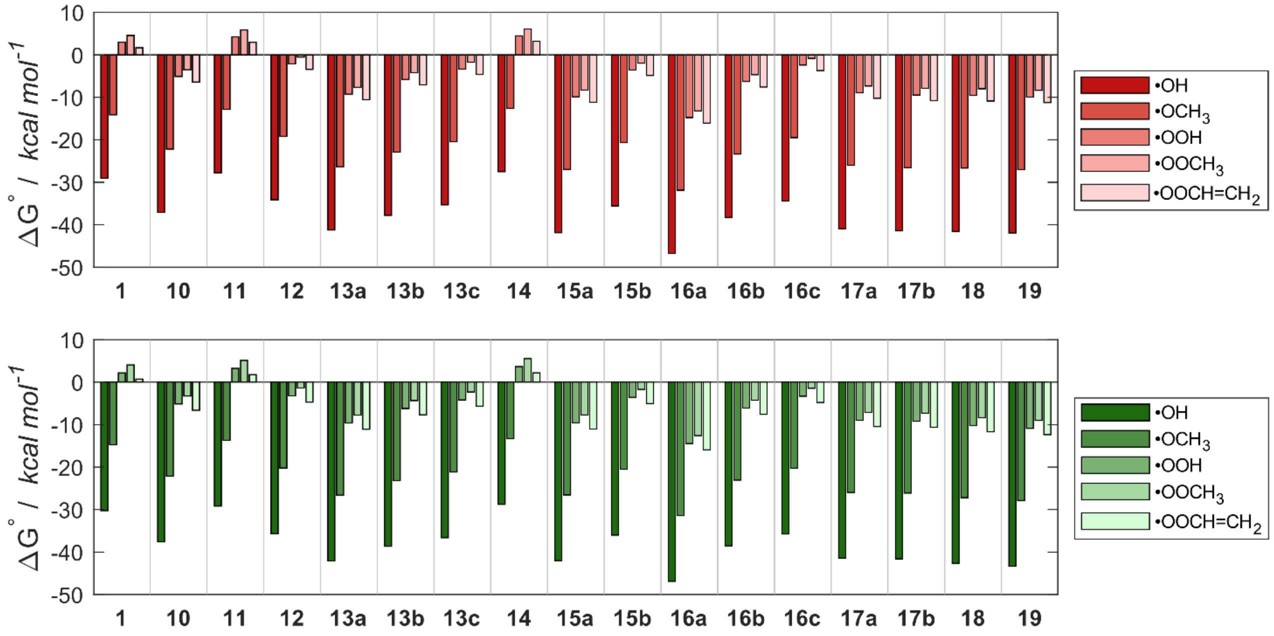

Figure S4 Gibbs free reaction energies (kcal mol^{-1}) computed in gas phase (red) and benzene (green) for the scavenging of $^{\bullet}$OH, $^{\bullet}OCH_3$, $^{\bullet}$OOH, $^{\bullet}OOCH_3$ and $^{\bullet}OOCH{=}CH_2$ via f-HAT from the OH site of molecules **1**, **10-19** (class B). Level of theory: (SMD)-M06-2X/6-311+G(d,p)//M06-2X/6-31G(d).

Table S2 Gibbs free reaction energies (ΔG°, kcal mol^{-1}) computed in the gas phase, water and benzene for the scavenging of $^{\bullet}$OH, $^{\bullet}OCH_3$, $^{\bullet}$OOH, $^{\bullet}OOCH_3$ and $^{\bullet}OOCH{=}CH_2$ via f-HAT from the OH site of molecules **10-19** (class B). Level of theory: (SMD)-M06-2X/6-311+G(d,p)//M06-2X/6-31G(d).

	**gas phase**					**water**					**benzene**				
	$^{\bullet}$OH	$^{\bullet}OCH_3$	$^{\bullet}$OOH	$^{\bullet}OOCH_3$	$^{\bullet}OOCH{=}CH_2$	$^{\bullet}$OH	$^{\bullet}OCH_3$	$^{\bullet}$OOH	$^{\bullet}OOCH_3$	$^{\bullet}OOCH{=}CH_2$	$^{\bullet}$OH	$^{\bullet}OCH_3$	$^{\bullet}$OOH	$^{\bullet}OOCH_3$	$^{\bullet}OOCH{=}CH_2$
**10**	-37.1	-22.2	-5.1	-3.5	-6.4	-35.0	-21.8	-4.5	-3.2	-6.8	-37.6	-22.1	-5.1	-3.3	-6.6
**11**	-27.8	-12.9	4.2	5.8	2.9	-29.2	-16.1	1.3	2.6	-1.0	-29.2	-13.7	3.2	5.1	1.8
**12**	-34.1	-19.2	-2.1	-0.5	-3.4	-35.8	-22.6	-5.3	-4.0	-7.5	-35.7	-20.2	-3.2	-1.4	-4.7
**13a**	-41.3	-26.3	-9.3	-7.7	-10.6	-40.2	-27.1	-9.7	-8.4	-12.0	-42.1	-26.6	-9.6	-7.8	-11.1
**13b**	-37.8	-22.9	-5.8	-4.2	-7.1	-37.0	-23.8	-6.5	-5.2	-8.7	-38.6	-23.2	-6.2	-4.3	-7.7
**13c**	-35.3	-20.4	-3.3	-1.7	-4.6	-36.3	-23.2	-5.8	-4.6	-8.1	-36.6	-21.2	-4.2	-2.3	-5.7
**14**	-27.5	-12.6	4.5	6.1	3.2	-28.6	-15.4	1.9	3.2	-0.3	-28.8	-13.3	3.7	5.5	2.2
**15a**	-41.9	-27.0	-9.9	-8.3	-11.2	-38.5	-25.3	-7.9	-6.7	-10.2	-42.0	-26.6	-9.6	-7.7	-11.1
**15b**	-35.6	-20.7	-3.6	-2.0	-4.9	-33.8	-20.6	-3.2	-2.0	-5.5	-36.0	-20.5	-3.6	-1.7	-5.0
**16a**	-46.8	-31.9	-14.8	-13.2	-16.1	-43.1	-30.0	-12.6	-11.4	-14.9	-46.9	-31.4	-14.5	-12.6	-15.9
**16b**	-38.3	-23.4	-6.3	-4.7	-7.6	-36.0	-22.8	-5.4	-4.2	-7.7	-38.5	-23.1	-6.1	-4.2	-7.6
**16c**	-34.4	-19.5	-2.4	-0.8	-3.7	-35.8	-22.7	-5.3	-4.1	-7.6	-35.8	-20.3	-3.3	-1.4	-4.8
**17a**	-40.9	-26.0	-8.9	-7.4	-10.2	-39.4	-26.3	-8.9	-7.7	-11.2	-41.4	-26.0	-9.0	-7.1	-10.5
**17b**	-41.5	-26.6	-9.5	-7.9	-10.8	-38.4	-25.3	-7.9	-6.6	-10.2	-41.6	-26.1	-9.2	-7.3	-10.6
**18**	-41.6	-26.7	-9.6	-8.0	-10.9	-41.4	-28.3	-10.9	-9.6	-13.2	-42.7	-27.2	-10.2	-8.4	-11.7
**19**	-41.9	-27.0	-10.0	-8.4	-11.2	-43.1	-29.9	-12.5	-11.3	-14.8	-43.3	-27.9	-10.9	-9.0	-12.4

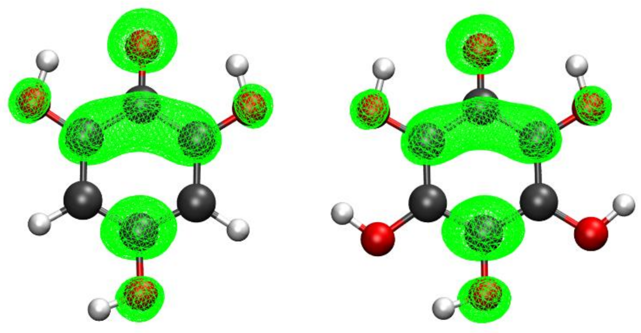

Figure S5 Spin densities of the radical products of f-HAT from the OH site of **16a** and **19**. Isosurface value of 0.003 a.u.. Level of theory: M06-2X/6-311+G(d,p)//M06-2X/6-31G(d).

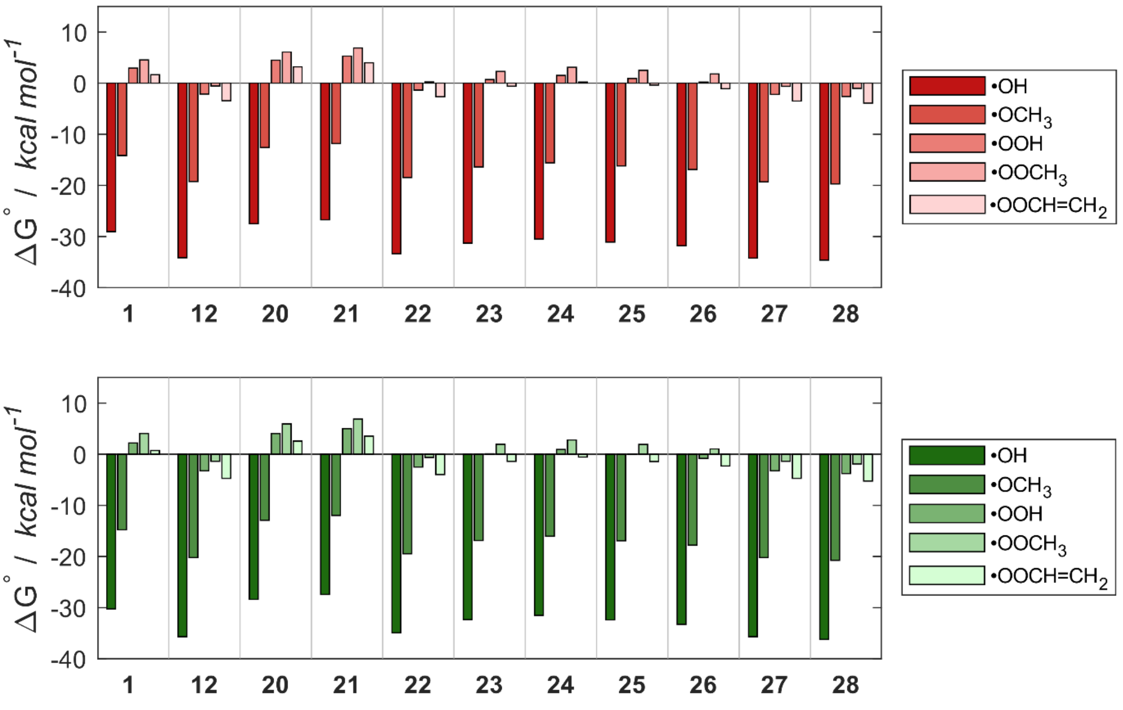

Figure S6 Gibbs free reaction energies (kcal mol^{-1}) computed in gas phase (red) and benzene (green) for the scavenging of $^{\bullet}$OH, $^{\bullet}OCH_3$, $^{\bullet}$OOH, $^{\bullet}OOCH_3$ and $^{\bullet}OOCH{=}CH_2$ via f-HAT from the OH site of molecules **1**, **12**, **20-28** (class C). Level of theory: (SMD)-M06-2X/6-311+G(d,p)//M06-2X/6-31G(d).

Table S3 Gibbs free reaction energies (ΔG°, kcal mol^{-1}) computed in the gas phase, water and benzene for the scavenging of $^{•}OH$, $^{•}OCH_3$, $^{•}OOH$, $^{•}OOCH_3$ and $^{•}OOCH{=}CH_2$ via f-HAT from the OH site of molecules **20-28** (class C). Level of theory: (SMD)-M06-2X/6-311+G(d,p)//M06-2X/6-31G(d).

	**gas phase**					**water**					**benzene**				
	$^{•}OH$	$^{•}OCH_3$	$^{•}OOH$	$^{•}OOCH_3$	$^{•}OOCH{=}CH_2$	$^{•}OH$	$^{•}OCH_3$	$^{•}OOH$	$^{•}OOCH_3$	$^{•}OOCH{=}CH_2$	$^{•}OH$	$^{•}OCH_3$	$^{•}OOH$	$^{•}OOCH_3$	$^{•}OOCH{=}CH_2$
**20**	-27.5	-12.6	4.5	6.1	3.2	-26.4	-13.3	4.1	5.4	1.8	-28.4	-12.9	4.1	5.9	2.6
**21**	-26.7	-11.8	5.3	6.9	4.0	-25.4	-12.3	5.1	6.4	2.8	-27.4	-12.0	5.0	6.9	3.5
**22**	-33.4	-18.5	-1.4	0.2	-2.7	-35.0	-21.8	-4.5	-3.2	-6.7	-34.9	-19.5	-2.5	-0.6	-4.0
**23**	-31.3	-16.4	0.7	2.3	-0.6	-31.0	-17.9	-0.5	0.7	-2.8	-32.4	-16.9	0.1	2.0	-1.4
**24**	-30.5	-15.6	1.5	3.1	0.2	-30.4	-17.3	0.1	1.4	-2.2	-31.5	-16.0	0.9	2.8	-0.5
**25**	-31.1	-16.2	0.9	2.5	-0.4	-31.8	-18.6	-1.2	0.0	-3.5	-32.4	-16.9	0.1	1.9	-1.4
**26**	-31.8	-16.9	0.2	1.8	-1.1	-33.0	-19.9	-2.5	-1.2	-4.8	-33.3	-17.8	-0.8	1.0	-2.3
**27**	-34.2	-19.3	-2.2	-0.6	-3.5	-35.7	-22.6	-5.2	-4.0	-7.5	-35.7	-20.2	-3.2	-1.4	-4.7
**28**	-34.6	-19.7	-2.6	-1.0	-3.9	-36.5	-23.4	-6.0	-4.8	-8.3	-36.2	-20.7	-3.8	-1.9	-5.2

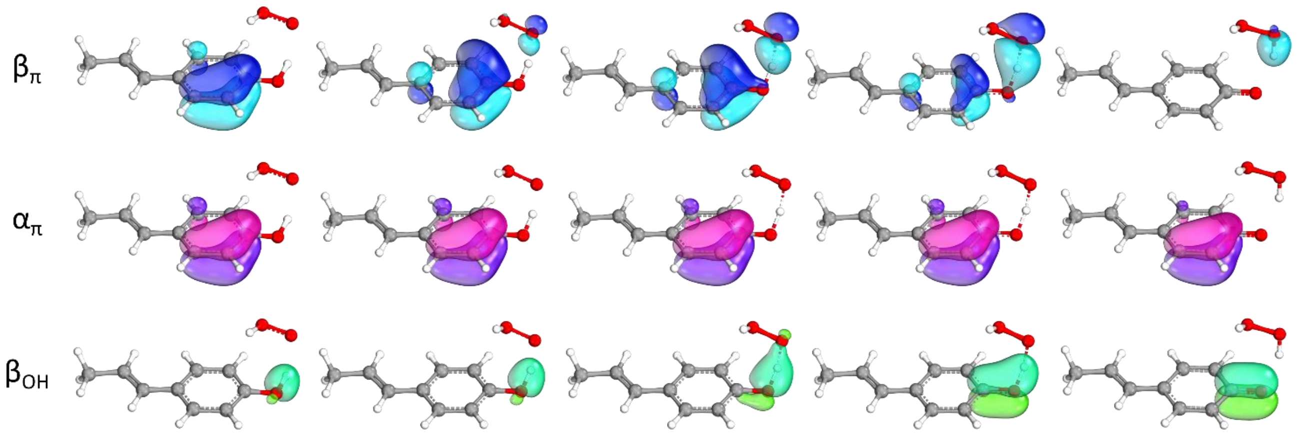

Figure S7 Changes in the main spin IBOs involved in the scavenging of $^{•}OOH$ via f-HAT from the OH site of **22** along the reaction path: β_π spin IBO (blue) transferred from the π system of the ring to the peroxyl radical and the corresponding α_π spin IBO (purple), β_{OH} spin IBO of the OH σ bond (green). Level of theory: M06-2X/def2TZVP//M06-2X/6-31G(d).

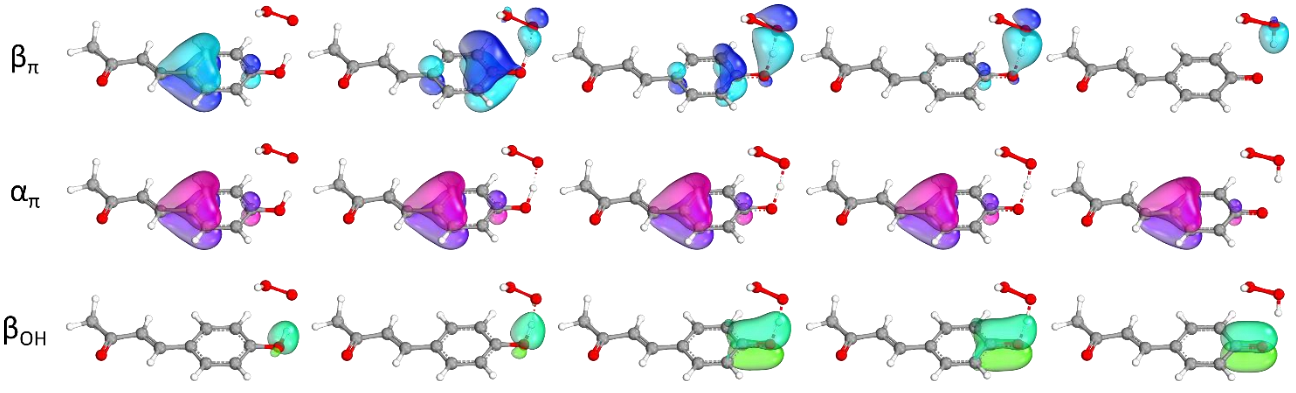

Figure S8 Changes in the main spin IBOs involved in the scavenging of $^{\bullet}$OOH via f-HAT from the OH site of **23** along the reaction path: β_{π} spin IBO (blue) transferred from the π system of the ring to the peroxyl radical and the corresponding α_{π} spin IBO (purple), β_{OH} spin IBO of the OH σ bond (green). Level of theory: M06-2X/def2TZVP//M06-2X/6-31G(d).

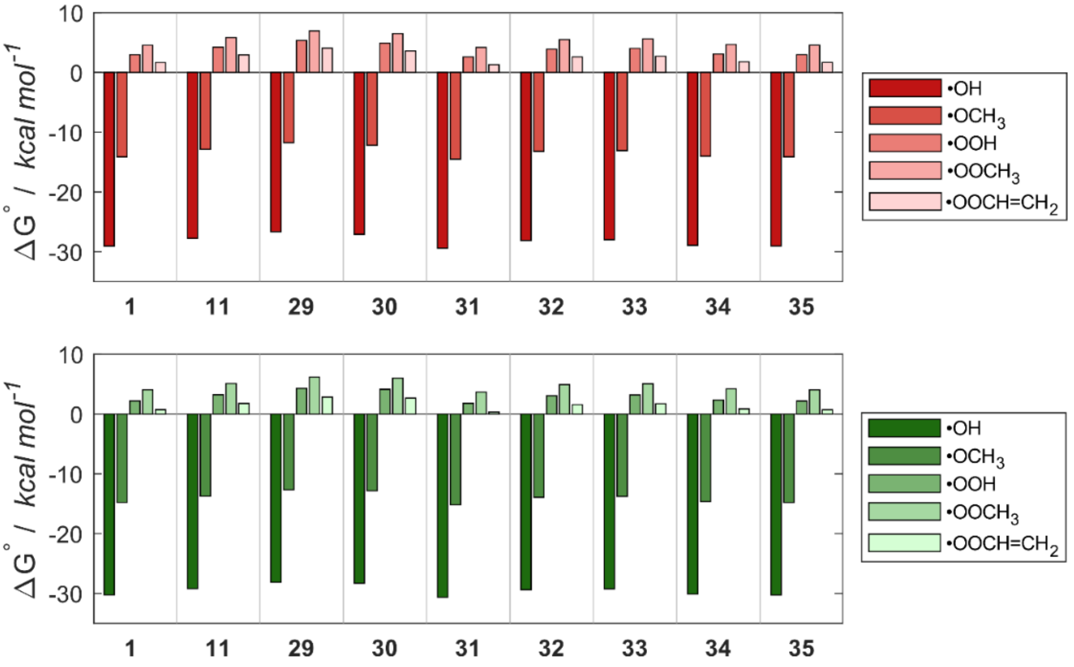

Figure S9 Gibbs free reaction energies (kcal mol^{-1}) computed in gas phase (red) and benzene (green) for the scavenging of $^{\bullet}$OH, $^{\bullet}OCH_3$, $^{\bullet}$OOH, $^{\bullet}OOCH_3$ and $^{\bullet}OOCH{=}CH_2$ via f-HAT from the OH site of molecules **1**, **11**, **29-35** (class D). Level of theory: (SMD)-M06-2X/6-311+G(d,p)//M06-2X/6-31G(d).

Table S4 Gibbs free reaction energies (ΔG°, kcal mol^{-1}) computed in the gas phase, water and benzene for the scavenging of $^{•}OH$, $^{•}OCH_3$, $^{•}OOH$, $^{•}OOCH_3$ and $^{•}OOCH=CH_2$ via f-HAT from the OH site of molecules **29-35** (class D). Level of theory: (SMD)-M06-2X/6-311+G(d,p)//M06-2X/6-31G(d).

	gas phase					water					benzene				
	$^{•}OH$	$^{•}OCH_3$	$^{•}OOH$	$^{•}OOCH_3$	$^{•}OOCH=CH_2$	$^{•}OH$	$^{•}OCH_3$	$^{•}OOH$	$^{•}OOCH_3$	$^{•}OOCH=CH_2$	$^{•}OH$	$^{•}OCH_3$	$^{•}OOH$	$^{•}OOCH_3$	$^{•}OOCH=CH_2$
**29**	-26.6	-11.7	5.3	6.9	4.1	-27.7	-14.5	2.9	4.1	0.6	-28.1	-12.7	4.3	6.2	2.8
**30**	-27.1	-12.2	4.9	6.5	3.6	-27.3	-14.2	3.2	4.5	0.9	-28.3	-12.8	4.1	6.0	2.7
**31**	-29.4	-14.5	2.6	4.2	1.3	-29.9	-16.7	0.6	1.9	-1.6	-30.6	-15.2	1.8	3.7	0.3
**32**	-28.1	-13.2	3.9	5.5	2.6	-28.6	-15.4	1.9	3.2	-0.3	-29.4	-13.9	3.1	4.9	1.6
**33**	-28.0	-13.1	4.0	5.6	2.7	-28.4	-15.2	2.2	3.4	-0.1	-29.2	-13.8	3.2	5.1	1.7
**34**	-28.9	-14.0	3.1	4.7	1.8	-29.2	-16.1	1.3	2.6	-1.0	-30.1	-14.6	2.3	4.2	0.9
**35**	-29.0	-14.1	3.0	4.6	1.7	-29.4	-16.3	1.1	2.3	-1.2	-30.3	-14.8	2.2	4.1	0.7

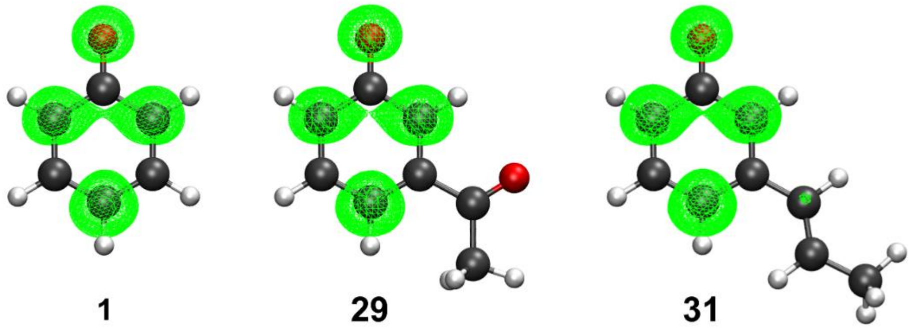

1 **29** **31**

Figure S10 Spin densities of the radical products of f-HAT from the OH site of **1**, **29** and **31**. Isosurface value of 0.003 a.u.. Level of theory: M06-2X/6-311+G(d,p)//M06-2X/6-31G(d).

Table S5 Gibbs free reaction energies (ΔG°, kcal mol^{-1}) computed in the gas phase, water and benzene for the scavenging of $^{•}OH$, $^{•}OCH_3$, $^{•}OOH$, $^{•}OOCH_3$ and $^{•}OOCH=CH_2$ via f-HAT from the OH site of molecules **36-42** (class E). Level of theory: (SMD)-M06-2X/6-311+G(d,p)//M06-2X/6-31G(d).

	gas phase					water					benzene				
	$^{•}OH$	$^{•}OCH_3$	$^{•}OOH$	$^{•}OOCH_3$	$^{•}OOCH=CH_2$	$^{•}OH$	$^{•}OCH_3$	$^{•}OOH$	$^{•}OOCH_3$	$^{•}OOCH=CH_2$	$^{•}OH$	$^{•}OCH_3$	$^{•}OOH$	$^{•}OOCH_3$	$^{•}OOCH=CH_2$
**36**	-17.1	-2.2	14.8	16.4	13.6	-24.6	-11.5	5.9	7.2	3.6	-20.0	-4.5	12.5	14.3	11.0
**37**	-22.8	-7.9	9.2	10.7	7.9	-26.7	-13.6	3.8	5.0	1.5	-25.4	-10.0	7.0	8.9	5.5
**38**	-33.1	-18.2	-1.1	0.5	-2.4	-33.3	-20.2	-2.8	-1.5	-5.1	-34.2	-18.7	-1.8	0.1	-3.2
**39**	-30.5	-15.6	1.5	3.1	0.2	-30.4	-17.2	0.1	1.4	-2.1	-31.5	-16.1	0.9	2.8	-0.6
**40**	-30.0	-15.0	2.0	3.6	0.7	-29.7	-16.6	0.8	2.1	-1.5	-31.0	-15.5	1.5	3.3	0.0
**41**	-31.2	-16.3	0.8	2.4	-0.5	-30.9	-17.7	-0.3	0.9	-2.6	-32.0	-16.6	0.4	2.3	-1.1
**42**	-33.6	-18.7	-1.6	0.0	-2.9	-33.6	-20.5	-3.1	-1.8	-5.4	-34.6	-19.1	-2.1	-0.3	-3.6

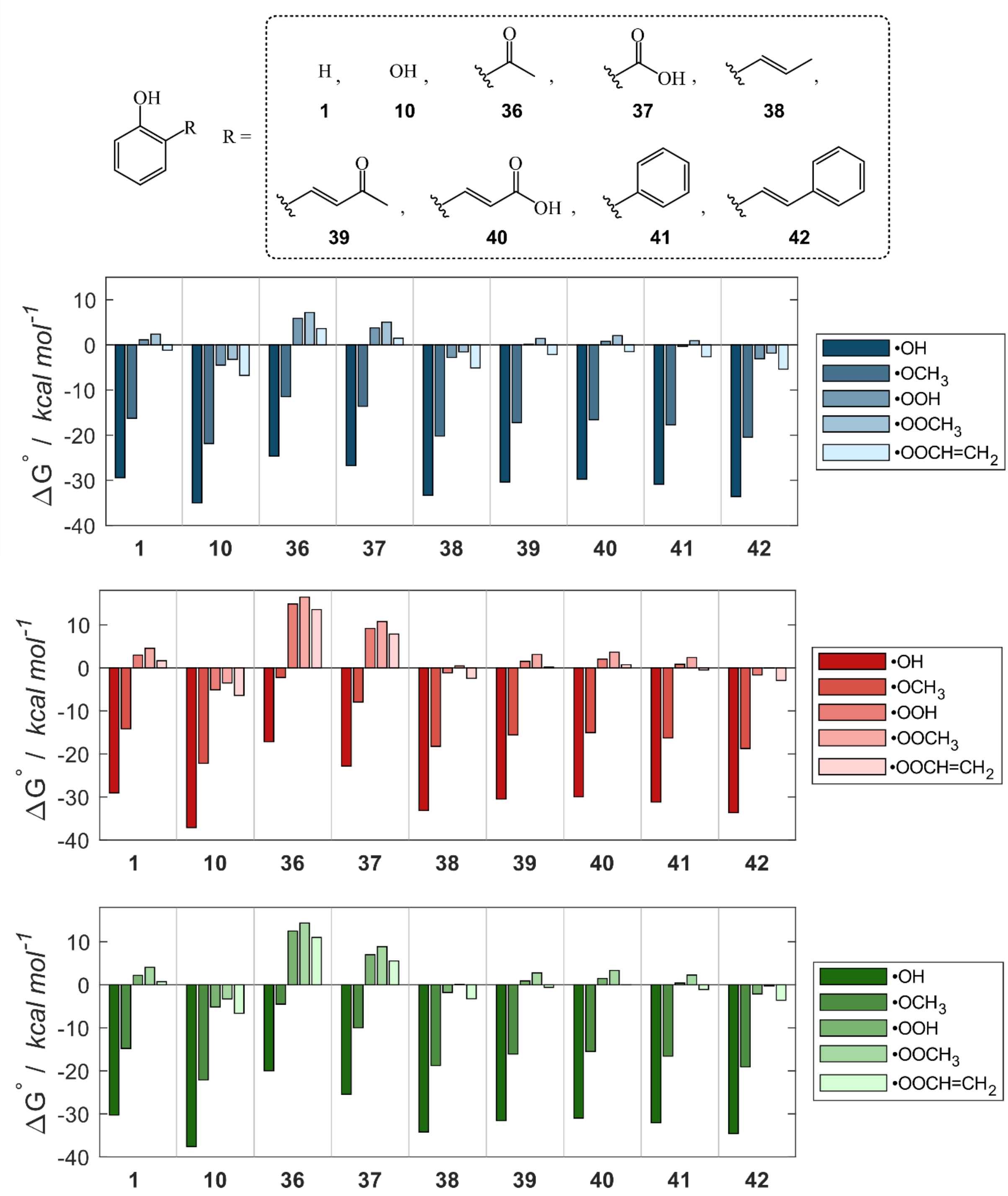

Figure S11 Chemical structure of class E compounds, ortho-substituted phenols, and Gibbs free reaction energies (kcal mol^{-1}) computed in water (blue), gas phase (red) and benzene (green) for the scavenging of $^{\bullet}$OH, $^{\bullet}OCH_3$, $^{\bullet}$OOH, $^{\bullet}OOCH_3$ and $^{\bullet}OOCH{=}CH_2$ via f-HAT from the OH sites of molecules **1**, **10**, **36-42**. Level of theory: (SMD)-M06-2X/6-311+G(d,p)//M06-2X/6-31G(d).

2

29

36

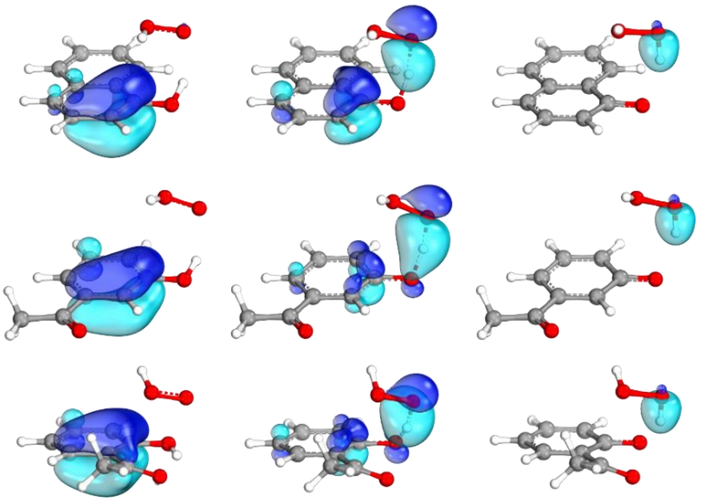

Figure S12 Changes in the β_π spin IBO involved in the scavenging of $^{\bullet}$OOH via f-HAT from the OH site of **2**, **29** and **36** along the reaction path. Level of theory: M06-2X/def2TZVP//M06-2X/6-31G(d).

To evaluate the effect of the position of a substituent on the scavenging mechanism, the IBO analysis was employed on one *ortho*- and one *meta*-substituted compound. **29** and **36** are chosen to maintain the same substituent as in the previous analysis, i.e. the ketone group. No significant differences are computed with respect to the *para*-substituted analogue (**20**), since Figure S12 shows a similar β_π spin IBO of the aromatic system being transferred to the ROS. Indeed, the CPET mechanism is retrieved, regardless of the position of a substituent on the aromatic ring. Furthermore, neither does the presence of an extended π system allow a change of mechanism, as in **2** the same conclusions on the electron flow can be drawn (Figure S12).

Table S6 Gibbs free reaction energies (ΔG°, kcal mol^{-1}) computed in the gas phase, water and benzene for the scavenging of •OH, •OCH_3, •OOH, •$OOCH_3$ and •$OOCH=CH_2$ via f-HAT from the OH site of anions **21an**, **24an**, **33an**, **37an**, **40an**, **43-49**. Level of theory: (SMD)-M06-2X/6-311+G(d,p)//M06-2X/6-31G(d).

	**gas phase**					**water**					**benzene**				
	•OH	•OCH_3	•OOH	•$OOCH_3$	•$OOCH=CH_2$	•OH	•OCH_3	•OOH	•$OOCH_3$	•$OOCH=CH_2$	•OH	•OCH_3	•OOH	•$OOCH_3$	•$OOCH=CH_2$
**21an**	-39.3	-24.4	-7.3	-5.7	-8.6	-29.3	-16.1	1.3	2.5	-1.0	-36.5	-21.1	-4.1	-2.2	-5.6
**24an**	-42.5	-27.6	-10.5	-8.9	-11.8	-32.4	-19.3	-1.9	-0.6	-4.2	-39.1	-23.6	-6.6	-4.8	-8.1
**30an**	-34.6	-19.7	-2.6	-1.0	-3.9	-29.6	-16.4	0.9	2.2	-1.3	-33.5	-18.0	-1.1	0.8	-2.5
**33an**	-33.0	-18.1	-1.0	0.6	-2.3	-29.2	-16.1	1.3	2.6	-1.0	-32.2	-16.7	0.2	2.1	-1.2
**37an**	-10.3	4.6	21.7	23.3	20.4	-23.2	-10.1	7.3	8.6	5.0	-14.2	1.3	18.3	20.1	16.8
**40an**	-38.8	-23.9	-6.8	-5.2	-8.1	-31.7	-18.5	-1.2	0.1	-3.4	-36.7	-21.2	-4.2	-2.3	-5.7
**43a**	-48.3	-33.4	-16.4	-14.8	-17.6	-49.8	-36.7	-19.3	-18.0	-21.6	-49.5	-34.0	-17.0	-15.2	-18.5
**43b**	-43.3	-28.4	-11.3	-9.7	-12.6	-41.7	-28.6	-11.2	-10.0	-13.5	-43.7	-28.2	-11.3	-9.4	-12.7
**43c**	-47.4	-32.5	-15.4	-13.8	-16.7	-42.2	-29.0	-11.7	-10.4	-14.0	-46.6	-31.1	-14.2	-12.3	-15.6
**44a**	-47.0	-32.1	-15.0	-13.4	-16.3	-49.7	-36.6	-19.2	-17.9	-21.5	-48.6	-33.1	-16.1	-14.3	-17.6
**44b**	-59.1	-44.2	-27.1	-25.5	-28.4	-52.2	-39.1	-21.7	-20.4	-24.0	-57.9	-42.4	-25.5	-23.6	-26.9
**45a**	-57.3	-42.4	-25.3	-23.7	-26.6	-46.0	-32.9	-15.5	-14.3	-17.8	-54.8	-39.3	-22.3	-20.5	-23.8
**45b**	-70.2	-55.3	-38.3	-36.7	-39.6	-56.0	-42.9	-25.5	-24.3	-27.8	-66.9	-51.4	-34.4	-32.6	-35.9
**46a**	-51.0	-36.1	-19.0	-17.5	-20.3	-50.6	-37.5	-20.1	-18.8	-22.4	-51.6	-36.1	-19.1	-17.3	-20.6
**46b**	-49.6	-34.7	-17.6	-16.1	-18.9	-46.0	-32.8	-15.4	-14.2	-17.7	-49.6	-34.1	-17.1	-15.2	-18.6
**46c**	-60.3	-45.4	-28.3	-26.8	-29.6	-54.1	-40.9	-23.6	-22.3	-25.8	-59.4	-44.0	-27.0	-25.1	-28.5
**47a**	-48.8	-33.9	-16.8	-15.2	-18.1	-47.4	-34.3	-16.9	-15.6	-19.2	-49.4	-33.9	-17.0	-15.1	-18.4
**47b**	-42.3	-27.4	-10.3	-8.7	-11.6	-42.0	-28.8	-11.5	-10.2	-13.8	-43.2	-27.7	-10.7	-8.8	-12.2
**47c**	-43.7	-28.8	-11.7	-10.1	-13.0	-46.7	-33.5	-16.1	-14.9	-18.4	-45.2	-29.7	-12.7	-10.8	-14.2
**48a**	-45.8	-30.9	-13.8	-12.2	-15.1	-51.9	-38.8	-21.4	-20.1	-23.7	-47.9	-32.4	-15.4	-13.5	-16.9
**48b**	-66.6	-51.7	-34.6	-33.1	-35.9	-56.5	-43.3	-25.9	-24.7	-28.2	-64.6	-49.1	-32.1	-30.2	-33.6
**48c**	-61.7	-46.8	-29.7	-28.1	-31.0	-52.2	-39.1	-21.7	-20.5	-24.0	-60.0	-44.5	-27.5	-25.6	-29.0
**49a**	-51.9	-37.0	-19.9	-18.4	-21.2	-50.4	-37.3	-19.9	-18.6	-22.2	-52.5	-37.1	-20.1	-18.2	-21.6
**49b**	-53.0	-38.1	-21.0	-19.4	-22.3	-48.1	-35.0	-17.6	-16.3	-19.9	-52.7	-37.2	-20.3	-18.4	-21.7
**49c**	-65.3	-50.4	-33.3	-31.7	-34.6	-56.6	-43.4	-26.0	-24.8	-28.3	-64.1	-48.6	-31.7	-29.8	-33.1

Table S7 O-H Bond Dissociation Enthalpies (kcal mol^{-1}) computed in the gas phase for the phenolic model systems of classes E and anions. Level of theory: M06-2X/6-311+G(d,p)//M06-2X/6-31G(d).

Class E	BDE	Anions	BDE	Anions	BDE
**36**	89.8	**21an**	66.5	**46a**	54.5
**37**	89.1	**24an**	63.0	**46b**	55.6
**38**	72.8	**30an**	71.2	**46c**	44.7
**39**	75.2	**33an**	72.8	**47a**	56.5
**40**	75.4	**37an**	97.2	**47b**	63.2
**41**	74.5	**40an**	67.5	**47c**	62.1
**42**	72.3	**43a**	57.2	**48a**	60.3
		**43b**	62.2	**48b**	38.1
		**43c**	58.1	**48c**	43.1
		**44a**	58.7	**49a**	52.7
		**44b**	46.2	**49b**	51.6
		**45a**	47.0	**49c**	38.9
		**45b**	33.7		

Table S8 Coordinates (Å), electronic energies (E, Hartree) and Gibbs free energies (G, Hartree) of stationary points and imaginary frequencies (Nimag, cm^{-1}) of transition states. Level of theory: M06-2X/6-31G(d).

$^{\bullet}$OH E= -75.688700 G= -75.697143
Nimag= 0

O	-0.006151000	0.000000000	-1.510008000
H	-0.006151000	0.000000000	-2.488867000

HOH E= -76.373400 G= -76.370181
Nimag= 0

O	0.019792000	-0.005769000	-1.526814000
H	0.002994000	-0.002034000	-2.492171000
H	-0.889189000	0.196357000	-1.271714000

$^{\bullet}$OCH$_3$ E= -114.9889688 G= -114.974437
Nimag= 0

C	-0.009738000	-0.577810000	0.000000000
H	1.056999000	-0.867510000	0.000000000
H	-0.460335000	-1.006509000	0.905992000
H	-0.460335000	-1.006509000	-0.905992000
O	-0.009738000	0.793424000	0.000000000
H	0.871595000	-1.051960000	0.000000000

HOCH$_3$ E= -115.6547578 G= -115.625324
Nimag= 0

C	-0.046785000	0.658091000	0.000000000
H	-1.091646000	0.973590000	0.000000000
H	0.437523000	1.077763000	0.891937000
H	0.437523000	1.077763000	-0.891937000
O	-0.046785000	-0.753213000	0.000000000

$^{\bullet}$OOH E= -150.832724 G= -150.840308
Nimag= 0

O	0.055503000	0.709875000	0.000000000
O	0.055503000	-0.601901000	0.000000000
H	-0.888054000	-0.863793000	0.000000000

HOOH E= -151.4681908 G= -151.462737
Nimag= 0

O	0.000000000	0.713705000	-0.055572000
O	0.000000000	-0.713705000	-0.055572000
H	-0.812252000	-0.891397000	0.444578000
H	0.812252000	0.891397000	0.444578000

$^{\bullet}$OOCH$_3$ E= -190.1247238 G= -190.106311
Nimag= 0

O	1.169320000	-0.278307000	0.000017000
O	0.157975000	0.542855000	-0.000016000
C	-1.081506000	-0.181434000	-0.000021000
H	-1.130743000	-0.801366000	-0.896590000
H	-1.131372000	-0.800256000	0.897305000
H	-1.867213000	0.573839000	-0.000590000

HOOCH$_3$ E= -190.7584410 G= -190.727715
Nimag= 0

O	-1.147069000	0.270796000	0.102545000
O	-0.025279000	-0.605400000	0.019773000
H	-1.569086000	0.104132000	-0.756207000
C	1.115476000	0.222340000	-0.021549000
H	1.963984000	-0.466065000	-0.031970000
H	1.125724000	0.842163000	-0.925757000
H	1.165302000	0.862565000	0.864682000

•OOCH=CH$_2$ E= -228.1915676 G= -228.169501

Nimag= 0

C	-1.389731000	-0.500063000	0.000186000
C	-0.628608000	0.583193000	-0.000384000
H	-0.954065000	-1.490586000	0.001155000
H	-2.466422000	-0.390841000	-0.000307000
H	-0.972696000	1.610046000	-0.001325000
O	0.762674000	0.580046000	0.000699000
O	1.300227000	-0.608472000	-0.000491000

HOOCH=CH$_2$ E= -228.8294479 G= -228.795188

Nimag= 0

C	1.406517000	-0.554260000	0.001289000
C	0.696641000	0.565615000	0.013829000
H	0.940238000	-1.527673000	-0.060358000
H	2.485961000	-0.488479000	0.033521000
H	1.124964000	1.562825000	0.054755000
O	-0.663060000	0.702224000	-0.016204000
O	-1.287489000	-0.566962000	-0.101344000
H	-1.565717000	-0.696899000	0.821765000

Reactants

1 E= -307.329665839 G= -307.252649

Nimag= 0

C	1.459724000	-0.002560000	-1.590836000
C	0.302766000	-0.357463000	-0.902738000
C	2.613494000	0.351389000	-0.898429000
H	-0.598328000	-0.633926000	-1.445875000
H	3.513008000	0.627411000	-1.438130000
C	0.300782000	-0.358135000	0.491987000
C	2.600694000	0.347461000	0.495887000
H	3.494895000	0.621866000	1.047414000
C	1.452993000	-0.004672000	1.194890000
H	1.424902000	-0.013201000	2.279137000
H	1.453956000	-0.004310000	-2.676608000
O	-0.797984000	-0.695042000	1.222970000
H	-1.523307000	-0.915719000	0.622098000

2 E= -460.91303836 G= -460.791865

Nimag= 0

O	1.073124000	2.755318000	0.214223000
C	2.383087000	0.739969000	0.126594000
C	2.416233000	-0.672447000	0.048361000
C	1.257831000	-1.399597000	-0.014138000
C	-0.000521000	-0.739010000	-0.001136000
C	-0.050137000	0.679046000	0.077189000
C	1.178851000	1.399770000	0.140615000
C	-1.303222000	1.341659000	0.090356000
C	-2.467493000	0.618635000	0.027723000
C	-2.427598000	-0.793884000	-0.050619000
C	-1.225437000	-1.453633000	-0.064593000
H	3.311608000	1.303752000	0.175884000
H	3.378711000	-1.174310000	0.038633000
H	1.282736000	-2.483613000	-0.074091000
H	-1.319150000	2.424278000	0.150657000
H	-3.425678000	1.128500000	0.038096000
H	-3.355984000	-1.354683000	-0.099913000
H	-1.190566000	-2.538244000	-0.124561000
H	1.960832000	3.138315000	0.250577000

3 E= -460.911292496 G= -460.790547

Nimag= 0

O	3.520532000	1.538622000	0.000000000
C	2.378574000	0.796565000	0.000000000
C	2.423235000	-0.621151000	0.000000000
C	1.260917000	-1.345450000	0.000000000
C	-0.002494000	-0.700797000	0.000000000
C	-0.039472000	0.722792000	0.000000000
C	1.173165000	1.453831000	0.000000000
C	-1.305428000	1.369635000	0.000000000
C	-2.465790000	0.640510000	0.000000000
C	-2.426700000	-0.776150000	0.000000000
C	-1.221323000	-1.428114000	0.000000000
H	3.387374000	-1.125228000	0.000000000
H	1.296791000	-2.431658000	0.000000000
H	1.160196000	2.539318000	0.000000000
H	-1.334972000	2.455949000	0.000000000
H	-3.425180000	1.148790000	0.000000000
H	-3.353976000	-1.340088000	0.000000000
H	-1.179303000	-2.514492000	0.000000000
H	4.285756000	0.947407000	0.000000000

4 E= -614.486154709 G= -614.322154

Nimag= 0

O	-0.018423000	-2.747706000	-0.086565000
C	3.664349000	0.599516000	0.019176000
C	3.617200000	-0.825440000	-0.015867000
C	2.418885000	-1.476996000	-0.040577000
C	1.194917000	-0.740471000	-0.029196000
C	2.513284000	1.330129000	0.026490000
C	1.234021000	0.690581000	0.001964000
C	-0.051017000	-1.388554000	-0.053087000
C	-1.255184000	-0.663899000	-0.032292000
C	0.042382000	1.418967000	0.000065000

C	-1.192322000	0.771926000	-0.017153000
C	-2.541524000	-1.291425000	-0.019465000
C	-3.687639000	-0.553273000	-0.014948000
C	-3.626995000	0.871515000	-0.019890000
C	-2.421676000	1.505804000	-0.016832000
H	4.627352000	1.100522000	0.038719000
H	4.544223000	-1.389898000	-0.023926000
H	2.370521000	-2.559689000	-0.068344000
H	2.544012000	2.416115000	0.051361000
H	0.076850000	2.505344000	0.014083000
H	-2.629478000	-2.375293000	0.010339000
H	-4.653010000	-1.048680000	-0.000050000
H	-4.548277000	1.445287000	-0.018922000
H	-2.366476000	2.591136000	-0.011856000
H	-0.903669000	-3.094164000	-0.257836000

5 E= -614.488839073 G= -614.323912
Nimag= 0

O	2.345067000	-2.794149000	0.087194000
C	3.678934000	0.647777000	0.019798000
C	3.649624000	-0.777236000	0.050051000
C	2.454787000	-1.438581000	0.058702000
C	1.211265000	-0.718136000	0.037586000
C	2.527895000	1.375179000	-0.000914000
C	1.257175000	0.715774000	0.007237000
C	-0.016051000	-1.378221000	0.045793000
C	-1.217649000	-0.664165000	0.024801000
C	0.053517000	1.422268000	-0.013539000
C	-1.182043000	0.769807000	-0.005565000
C	-2.487946000	-1.321218000	0.032650000
C	-3.646763000	-0.603329000	0.011749000
C	-3.611505000	0.823478000	-0.018528000
C	-2.420221000	1.486342000	-0.026860000
H	4.642429000	1.147686000	0.013526000
H	4.581569000	-1.336987000	0.066270000
H	2.552131000	2.460511000	-0.023825000
H	-0.034772000	-2.463499000	0.068783000
H	0.079125000	2.509694000	-0.036570000
H	-2.506427000	-2.407626000	0.055691000
H	-4.605615000	-1.112063000	0.017975000
H	-4.544468000	1.378320000	-0.035002000
H	-2.388563000	2.572517000	-0.049868000
H	3.232137000	-3.180681000	0.098381000

6 E= -614.486522462 G= -614.322243
Nimag= 0

O	4.771029000	-1.575333000	0.000000000
C	3.672444000	0.585665000	0.000000000
C	3.623009000	-0.843801000	0.000000000
C	2.425415000	-1.498599000	0.000000000
C	1.201042000	-0.765285000	0.000000000
C	2.521646000	1.312651000	0.000000000
C	1.244060000	0.670830000	0.000000000
C	-0.044145000	-1.400516000	0.000000000
C	-1.236714000	-0.674436000	0.000000000
C	0.052329000	1.397410000	0.000000000
C	-1.190064000	0.760832000	0.000000000
C	-2.516417000	-1.316255000	0.000000000
C	-3.666288000	-0.584946000	0.000000000
C	-3.618742000	0.842766000	0.000000000
C	-2.420372000	1.491455000	0.000000000
H	4.640143000	1.082723000	0.000000000
H	2.410258000	-2.583720000	0.000000000
H	2.558378000	2.398669000	0.000000000
H	-0.084798000	-2.487553000	0.000000000
H	0.092861000	2.484944000	0.000000000
H	-2.548433000	-2.402615000	0.000000000
H	-4.630338000	-1.084045000	0.000000000
H	-4.546375000	1.406365000	0.000000000
H	-2.375551000	2.577522000	0.000000000
H	5.532171000	-0.979206000	0.000000000

7 E= -768.059035831 G= -767.851830
Nimag= 0

O	-0.131376000	-2.899747000	-0.053687000
C	-1.286766000	0.580046000	0.008238000
C	-1.287963000	-0.859106000	-0.017471000
C	-2.508668000	-1.555656000	-0.029779000
C	-3.721183000	-0.879911000	-0.017746000
C	-3.729344000	0.562113000	0.007917000
C	-2.524932000	1.250206000	0.020182000
C	-4.995500000	1.239266000	0.020004000
C	-6.161592000	0.539955000	0.007613000
C	-6.153282000	-0.893016000	-0.017923000
C	-4.976789000	-1.574837000	-0.030074000
H	-2.496084000	-2.640785000	-0.049008000
H	-2.530935000	2.337854000	0.039548000
H	-4.997867000	2.325924000	0.039322000
H	-7.112305000	1.063978000	0.017035000
H	-7.097084000	-1.429019000	-0.027507000
H	-4.962024000	-2.661379000	-0.049397000
C	2.401249000	1.284644000	0.021232000
C	-0.059380000	1.270838000	0.020875000
C	3.604968000	-0.814460000	-0.018248000
C	1.144607000	0.587565000	0.008481000
C	-0.046946000	-1.544813000	-0.029733000
C	1.167545000	-0.857265000	-0.017907000
C	3.584122000	0.616856000	0.008668000
C	2.442235000	-1.519122000	-0.030971000
H	4.556921000	-1.335150000	-0.028776000
H	2.503363000	-2.605247000	-0.052276000
H	4.522262000	1.162523000	0.018712000

H	2.377343000	2.370914000	0.041324000
H	-0.060856000	2.357521000	0.040586000
H	0.753567000	-3.287023000	-0.055842000

8 E= -768.061339467 G= -767.852805
Nimag= 0

O	2.360785000	-2.878901000	-0.009249000
C	-1.284686000	0.579559000	0.009512000
C	-1.274479000	-0.862151000	0.000593000
C	-2.500292000	-1.554337000	-0.004368000
C	-3.714242000	-0.883520000	-0.000840000
C	-3.725065000	0.560095000	0.008171000
C	-2.522578000	1.251131000	0.013080000
C	-4.992631000	1.235292000	0.011746000
C	-6.157872000	0.535135000	0.006773000
C	-6.146888000	-0.898487000	-0.002210000
C	-4.970293000	-1.579412000	-0.005856000
H	-2.489621000	-2.642243000	-0.011112000
H	-2.531331000	2.339100000	0.019828000
H	-4.995868000	2.322121000	0.018530000
H	-7.109399000	1.057585000	0.009575000
H	-7.090006000	-1.435848000	-0.006095000
H	-4.955466000	-2.666159000	-0.012644000
C	2.414401000	1.299906000	0.015365000
C	-0.056191000	1.270425000	0.014305000
C	3.599864000	-0.822532000	0.003371000
C	1.158329000	0.602910000	0.010792000
C	-0.036715000	-1.537953000	-0.002869000
C	1.158079000	-0.840446000	0.002092000
C	3.583639000	0.608361000	0.011624000
C	2.430285000	-1.520901000	-0.001090000
H	4.550149000	-1.350602000	0.000966000
H	4.531866000	1.136670000	0.014727000
H	2.405510000	2.385687000	0.021617000
H	-0.065221000	2.358206000	0.020855000
H	-0.021559000	-2.623409000	-0.009441000
H	3.259096000	-3.238966000	-0.010989000

9 E= -768.058846685 G= -767.851051
Nimag= 0

O	4.776577000	-1.495235000	-0.027617000
C	-1.288553000	0.580869000	0.001283000
C	-1.274197000	-0.861924000	-0.003420000
C	-2.503423000	-1.551289000	-0.001119000
C	-3.715859000	-0.879024000	0.005493000
C	-3.728397000	0.565107000	0.010199000
C	-2.525626000	1.254257000	0.008007000
C	-4.995589000	1.241012000	0.017044000
C	-6.161072000	0.541665000	0.019076000
C	-6.149089000	-0.892292000	0.014398000
C	-4.973134000	-1.573942000	0.007878000
H	-2.495911000	-2.639237000	-0.004650000
H	-2.532532000	2.342376000	0.011525000
H	-4.998170000	2.327916000	0.020562000
H	-7.112657000	1.063865000	0.024302000
H	-7.092153000	-1.429894000	0.016152000
H	-4.959475000	-2.660700000	0.004337000
C	2.404915000	1.291995000	-0.009941000
C	-0.060483000	1.270125000	-0.001004000
C	3.594297000	-0.820415000	-0.021072000
C	1.150090000	0.595747000	-0.007597000
C	-0.037498000	-1.535740000	-0.010136000
C	1.168743000	-0.849113000	-0.012325000
C	3.581755000	0.615326000	-0.016423000
C	2.430183000	-1.526364000	-0.019103000
H	2.463413000	-2.611109000	-0.022708000
H	4.528063000	1.152318000	-0.018205000
H	2.395450000	2.378528000	-0.006441000
H	-0.066601000	2.358251000	0.002503000
H	-0.031966000	-2.623349000	-0.013668000
H	5.508011000	-0.862961000	-0.028511000

10 E= -382.523985955 G= -382.444272
Nimag= 0

O	-1.581244000	-0.704332000	-2.208298000
C	0.698672000	-0.017786000	-2.257156000
C	-0.447313000	-0.351614000	-1.550202000
C	1.859650000	0.343338000	-1.572906000
H	2.751262000	0.602820000	-2.133943000
C	-0.427117000	-0.322514000	-0.148623000
C	1.876384000	0.371136000	-0.183684000
H	2.777338000	0.651436000	0.351222000
C	0.725354000	0.036014000	0.532050000
H	0.723598000	0.053388000	1.619637000
H	0.661415000	-0.046911000	-3.340896000
O	-1.612646000	-0.670006000	0.449842000
H	-1.517517000	-0.629323000	1.410951000
H	-2.259825000	-0.899136000	-1.542160000

11 E= -382.523528444 G= -382.443690
Nimag= 0

O	0.744423000	0.058883000	-3.601347000
C	0.693470000	0.034807000	-2.242096000
C	-0.467599000	-0.301526000	-1.553587000
C	1.863580000	0.363982000	-1.552487000
H	-1.385228000	-0.559902000	-2.074632000
H	2.752072000	0.621876000	-2.116730000
C	-0.458907000	-0.308996000	-0.159234000
C	1.849731000	0.350297000	-0.165615000
H	2.753688000	0.604726000	0.379156000
C	0.698347000	0.016584000	0.545320000
H	0.697916000	0.008796000	1.631991000

O	-1.624513000	-0.645688000	0.457844000
H	-1.492940000	-0.615847000	1.415729000
H	-0.121704000	-0.185827000	-3.956194000

12 E= -382.519789981 G= -382.440392

Nimag= 0

O	3.018409000	0.688768000	-2.186776000
C	0.693979000	-0.001551000	-2.231952000
C	-0.458512000	-0.336346000	-1.530590000
C	1.857808000	0.350980000	-1.549511000
H	-1.369718000	-0.612283000	-2.050207000
C	-0.456921000	-0.321427000	-0.138173000
C	1.859412000	0.365882000	-0.157092000
H	2.770634000	0.641779000	0.362519000
C	0.706909000	0.031133000	0.544261000
H	0.715135000	0.044914000	1.631915000
H	0.685726000	-0.015317000	-3.319607000
O	-1.617499000	-0.659273000	0.499126000
H	-1.478251000	-0.608482000	1.454650000
H	2.879266000	0.637734000	-3.142302000

13 E= -457.714841545 G= -457.632529

Nimag= 0

C	0.788795000	0.046187000	-2.311167000
C	-0.371556000	-0.252113000	-1.593907000
C	1.950614000	0.397994000	-1.634243000
H	-1.287544000	-0.528779000	-2.104657000
C	-0.377731000	-0.201091000	-0.210594000
C	1.958938000	0.453366000	-0.238321000
H	2.874920000	0.729811000	0.275877000
C	0.801218000	0.155261000	0.459942000
H	0.781437000	0.002891000	-3.397038000
O	-1.509179000	-0.491769000	0.489828000
H	-1.301472000	-0.398968000	1.432913000
O	3.121445000	0.702535000	-2.268240000
H	2.987782000	0.626902000	-3.223048000
O	0.701167000	0.178993000	1.826047000
H	1.550122000	0.433331000	2.212432000

14 E= -457.717913538 G= -457.635106

Nimag= 0

O	3.090984000	0.694615000	-2.257762000
C	0.776002000	0.041548000	-2.315790000
C	-0.378746000	-0.255259000	-1.589832000
C	1.928346000	0.392106000	-1.621264000
C	-0.392837000	-0.206094000	-0.200684000
C	1.945495000	0.450315000	-0.226658000
H	2.863068000	0.727706000	0.282176000
C	0.779824000	0.149213000	0.468416000
H	0.750599000	-0.006990000	-3.399508000
H	2.956359000	0.619505000	-3.212843000
O	0.720077000	0.184292000	1.826272000
H	1.585611000	0.442226000	2.173096000
H	-1.284433000	-0.434680000	0.374088000
O	-1.481164000	-0.592645000	-2.311285000
H	-2.211718000	-0.775025000	-1.703844000

15 E= -457.718501687 G= -457.636024

Nimag= 0

C	0.665781000	-0.007717000	-2.184051000
C	-0.479461000	-0.333544000	-1.460916000
C	1.831559000	0.330317000	-1.505297000
H	2.724006000	0.584262000	-2.067172000
C	-0.447906000	-0.318066000	-0.067899000
C	1.872559000	0.348328000	-0.111796000
H	2.783461000	0.612491000	0.418339000
C	0.723798000	0.021600000	0.600407000
H	0.618851000	-0.026091000	-3.266809000
O	-1.598555000	-0.645377000	0.596254000
H	-1.418294000	-0.591285000	1.548060000
O	-1.626806000	-0.666080000	-2.104144000
H	-2.298290000	-0.857021000	-1.429241000
O	0.626103000	-0.000397000	1.968465000
H	1.475911000	0.245600000	2.358114000

16 E= -532.908525761 G= -532.823650

Nimag= 0

C	0.594256000	0.007348000	-2.245415000
C	-0.559273000	-0.331926000	-1.537248000
C	1.760539000	0.351971000	-1.572254000
H	2.653846000	0.614737000	-2.124918000
C	-0.538016000	-0.324008000	-0.150792000
C	1.764128000	0.354790000	-0.179936000
C	0.619817000	0.018279000	0.545115000
H	0.625394000	0.021482000	1.632770000
O	-1.721313000	-0.672161000	0.446090000
H	-1.623822000	-0.645570000	1.407299000
O	-1.676958000	-0.662506000	-2.261846000
H	-2.387039000	-0.870357000	-1.635020000
O	2.932209000	0.700062000	0.434378000
H	2.810020000	0.666434000	1.392739000
O	0.571197000	-0.001151000	-3.599739000
H	-0.321647000	-0.265156000	-3.876232000

17 E= -532.910478065 G= -532.825433

Nimag= 0

O	-1.597801000	-0.666455000	-2.190902000
O	-1.601552000	-0.634937000	0.520996000
C	0.696519000	-0.003548000	-2.226936000
C	-0.457908000	-0.328832000	-1.528981000
C	1.861404000	0.341766000	-1.536625000
H	2.764322000	0.596803000	-2.084619000

C	-0.446116000	-0.308541000	-0.131178000
C	1.866945000	0.359957000	-0.150178000
C	0.707871000	0.033210000	0.554037000
H	0.672689000	-0.023414000	-3.310224000
H	-1.433805000	-0.574939000	1.475072000
H	-2.278640000	-0.855574000	-1.525553000
O	2.943695000	0.681543000	0.642929000
H	3.706752000	0.882453000	0.084809000
O	0.655365000	0.034198000	1.919512000
H	1.532523000	0.289772000	2.247376000

18 E= -532.907081486 G= -532.822805
Nimag= 0

O	2.993533000	0.714662000	0.535562000
C	0.685587000	0.020222000	-2.225934000
C	-0.464157000	-0.320351000	-1.522360000
C	1.840705000	0.365922000	-1.536410000
C	-0.443564000	-0.310311000	-0.126052000
C	1.861351000	0.375827000	-0.140111000
C	0.711523000	0.035490000	0.563466000
H	0.740451000	0.047167000	1.649215000
H	0.656615000	0.008727000	-3.311686000
O	-1.628697000	-0.661507000	0.475016000
H	-1.527671000	-0.632425000	1.435678000
O	-1.596256000	-0.659469000	-2.198028000
H	-2.277414000	-0.861835000	-1.536986000
H	3.674913000	0.916294000	-0.125478000
O	3.025711000	0.717546000	-2.137512000
H	2.926548000	0.681979000	-3.098151000

19 E= -683.295731216 G= -683.205555
Nimag= 0

O	-1.554350000	-0.639486000	-2.225914000
C	0.709350000	0.030177000	-2.244882000
C	-0.427839000	-0.309490000	-1.521185000
C	1.880230000	0.373598000	-1.578884000
C	-0.394429000	-0.305700000	-0.131501000
C	1.913656000	0.377271000	-0.189239000
C	0.776399000	0.037711000	0.534501000
O	-1.541693000	-0.647802000	0.532843000
H	-1.357707000	-0.596485000	1.484314000
H	-2.252315000	-0.848993000	-1.585017000
O	3.040104000	0.707329000	0.515582000
H	3.737645000	0.918402000	-0.125258000
O	3.027327000	0.716032000	-2.243332000
H	2.843236000	0.664861000	-3.194797000
O	0.756218000	0.025634000	1.903425000
H	1.637826000	0.287568000	2.213244000
O	0.729663000	0.042156000	-3.613813000
H	-0.152324000	-0.218414000	-3.923693000

20 E= -459.917779030 G= -459.807530
Nimag= 0

C	-0.648677000	-1.399761000	-0.493833000
C	0.746104000	-1.451863000	-0.538730000
H	1.222812000	-2.360366000	-0.889806000
C	1.484150000	-0.349262000	-0.136553000
H	2.568018000	-0.402095000	-0.176230000
C	0.850858000	0.814826000	0.313629000
C	-0.546020000	0.850608000	0.352453000
C	-1.295895000	-0.244068000	-0.046506000
H	-2.382550000	-0.210003000	-0.013780000
C	1.601284000	2.027487000	0.756973000
O	1.011877000	3.017759000	1.141120000
C	3.116185000	1.993313000	0.716265000
H	3.472837000	1.814217000	-0.303199000
H	3.503150000	1.188011000	1.349000000
H	3.494968000	2.951051000	1.072307000
H	-1.026660000	1.758127000	0.703256000
O	-1.324687000	-2.503790000	-0.897289000
H	-2.276213000	-2.344230000	-0.820772000

21 E= -495.842405929 G= -495.754094
Nimag= 0

C	-0.631728000	-1.389687000	-0.480584000
C	0.764190000	-1.427442000	-0.541314000
H	1.245496000	-2.334271000	-0.890292000
C	1.496600000	-0.316102000	-0.158282000
H	2.580007000	-0.336120000	-0.201992000
C	0.845889000	0.839288000	0.287462000
C	-0.547890000	0.868809000	0.345187000
C	-1.289047000	-0.238982000	-0.036486000
H	-2.375288000	-0.217187000	0.008018000
C	1.583833000	2.052555000	0.707595000
O	1.071149000	3.074753000	1.099240000
H	-1.035373000	1.773665000	0.693239000
O	-1.298214000	-2.504816000	-0.867306000
H	-2.250910000	-2.357363000	-0.781581000
O	2.923759000	1.914380000	0.613064000
H	3.301339000	2.760029000	0.909224000

22 E= -423.990522637 G= -423.857812
Nimag= 0

H	1.870748000	-1.768837000	-0.354095000
C	1.007138000	-1.128726000	-0.200448000
C	-0.250814000	-1.709854000	-0.113311000
H	-0.359025000	-2.789945000	-0.186285000
C	-1.380846000	-0.907956000	0.053784000
C	-1.240594000	0.477101000	0.126337000
C	0.023084000	1.042172000	0.038588000
C	1.174753000	0.258372000	-0.115310000
C	2.490886000	0.913577000	-0.186225000

C	3.684027000	0.329425000	-0.048509000
H	2.462137000	1.991154000	-0.353955000
H	3.739520000	-0.740174000	0.152455000
C	4.992308000	1.054549000	-0.139459000
H	5.569817000	0.947698000	0.785862000
H	5.612568000	0.652537000	-0.948935000
H	4.839602000	2.121192000	-0.326014000
H	0.125381000	2.122896000	0.097256000
O	-2.639262000	-1.420260000	0.141995000
H	-2.594380000	-2.384373000	0.076621000
H	-2.128922000	1.087246000	0.249216000

23 E= -537.281316939 G= -537.141841

Nimag= 0

H	1.924124000	-1.756166000	-0.039195000
C	1.052770000	-1.108524000	-0.029849000
C	-0.208610000	-1.682824000	-0.034399000
H	-0.317273000	-2.764912000	-0.047388000
C	-1.345552000	-0.869226000	-0.022242000
C	-1.208576000	0.519135000	-0.005398000
H	-2.102406000	1.133037000	0.004082000
C	0.059102000	1.078207000	-0.000984000
C	1.215331000	0.283546000	-0.013158000
C	2.523385000	0.935915000	-0.008074000
C	3.733931000	0.358574000	-0.020374000
C	4.958203000	1.202123000	-0.012362000
O	4.908430000	2.416307000	0.005809000
H	2.517113000	2.026112000	0.007358000
H	3.866644000	-0.720682000	-0.036567000
C	6.279240000	0.461942000	-0.028348000
H	7.102387000	1.176278000	-0.020105000
H	6.345509000	-0.170628000	-0.920315000
H	6.351541000	-0.198082000	0.843021000
H	0.164561000	2.159761000	0.012227000
O	-2.605088000	-1.374148000	-0.026160000
H	-2.564818000	-2.340943000	-0.036594000

24 E= -573.205817691 G= -573.087701

Nimag= 0

H	1.936032000	-1.724316000	-0.033987000
C	1.060659000	-1.082270000	-0.020380000
C	-0.194104000	-1.661963000	-0.015657000
H	-0.322762000	-2.738782000	-0.025323000
C	-1.334633000	-0.851007000	0.002020000
C	-1.202855000	0.536758000	0.014895000
C	0.064798000	1.104634000	0.009948000
C	1.218843000	0.314114000	-0.007733000
C	2.525022000	0.969376000	-0.012243000
C	3.730851000	0.385251000	-0.029282000
C	4.938315000	1.232250000	-0.030901000
O	4.962124000	2.441654000	-0.017940000
H	2.517337000	2.059225000	-0.000463000
H	3.882947000	-0.688496000	-0.042365000
O	6.066778000	0.490269000	-0.049100000
H	6.805621000	1.122726000	-0.048875000
H	0.163828000	2.186771000	0.020027000
H	-2.087713000	1.168534000	0.028653000
O	-2.538529000	-1.476037000	0.005832000
H	-3.245170000	-0.814901000	0.018904000

25 E= -538.291534269 G= -538.139414

Nimag= 0

C	-1.175253000	0.010489000	-3.742434000
C	-1.557071000	1.146412000	-4.461815000
C	-0.783750000	-1.123030000	-4.467922000
C	-1.550521000	1.156840000	-5.852020000
H	-1.889821000	2.031225000	-3.926548000
C	-0.772908000	-1.127322000	-5.854432000
H	-0.457998000	-2.010386000	-3.932432000
C	-1.158101000	0.017585000	-6.553206000
H	-1.861083000	2.047909000	-6.393244000
H	-0.460477000	-2.000874000	-6.416521000
C	-1.184608000	0.005016000	-2.259742000
C	-1.587692000	-1.132236000	-1.549548000
C	-0.791271000	1.136727000	-1.535434000
H	-0.448560000	2.017968000	-2.070694000
H	-1.921748000	-2.009854000	-2.096032000
C	-0.803342000	1.133139000	-0.144623000
C	-1.595416000	-1.138782000	-0.158714000
H	-0.488200000	2.019107000	0.398337000
H	-1.917613000	-2.028936000	0.373121000
C	-1.204452000	-0.005399000	0.549836000
H	-1.211968000	-0.009492000	1.635311000
O	-1.129636000	-0.037515000	-7.912783000
H	-1.413178000	0.814912000	-8.271717000

26 E= -613.484415675 G= -613.329590

Nimag= 0

C	-1.168785000	0.012220000	-3.739633000
C	-1.552914000	1.147734000	-4.458668000
C	-0.783498000	-1.122134000	-4.467435000
C	-1.551704000	1.157990000	-5.849132000
H	-1.883177000	2.033090000	-3.922562000
C	-0.781861000	-1.128029000	-5.854208000
H	-0.455870000	-2.009781000	-3.933437000
C	-1.166652000	0.017386000	-6.551721000
H	-1.862791000	2.049749000	-6.388989000
H	-0.475732000	-2.003462000	-6.416855000
C	-1.168734000	0.007405000	-2.257781000
C	-1.567609000	-1.126868000	-1.537187000
C	-0.770853000	1.133498000	-1.531451000
H	-0.429829000	2.018216000	-2.061854000

C	-0.771880000	1.134854000	-0.140996000
C	-1.569268000	-1.141565000	-0.150554000
H	-0.449904000	2.019247000	0.404602000
C	-1.170719000	-0.005391000	0.554523000
O	-1.146080000	-0.038631000	-7.912127000
H	-1.429866000	0.814573000	-8.268925000
H	-1.905987000	-2.007082000	-2.076722000
H	-1.885780000	-2.016817000	0.406626000
O	-1.191637000	-0.069986000	1.914481000
H	-0.898120000	0.777502000	2.276945000

27 E= -615.656740702 G= -615.475305

Nimag= 0

H	1.951773000	-1.580557000	-0.667931000
C	1.086437000	-1.006196000	-0.351345000
C	-0.155533000	-1.624879000	-0.323918000
H	-0.249472000	-2.672153000	-0.602813000
C	-1.289535000	-0.899369000	0.046114000
C	-1.169722000	0.449256000	0.380669000
C	0.077683000	1.053175000	0.349419000
C	1.234369000	0.342645000	-0.003718000
C	2.530873000	1.029524000	-0.000835000
C	3.737629000	0.444271000	-0.015400000
H	2.474081000	2.116498000	0.047216000
H	8.314280000	0.489866000	0.629005000
C	7.431954000	1.057041000	0.348482000
C	6.188840000	0.434286000	0.325698000
H	6.105058000	-0.616322000	0.592535000
C	5.031112000	1.140743000	-0.025799000
C	5.162383000	2.491675000	-0.379365000
H	4.288000000	3.052166000	-0.696296000
C	6.402864000	3.115689000	-0.355065000
H	6.482927000	4.161846000	-0.634719000
C	7.543834000	2.402980000	0.011583000
H	8.512793000	2.892008000	0.024941000
H	3.799840000	-0.642925000	0.016321000
H	0.164820000	2.104114000	0.613382000
H	-2.061520000	1.000388000	0.658755000
O	-2.532660000	-1.450019000	0.087732000
H	-2.476688000	-2.382529000	-0.163824000

28 E= -690.849912429 G= -690.665763

Nimag= 0

H	1.959592000	-1.574159000	-0.660742000
C	1.090344000	-1.002589000	-0.349726000
C	-0.149455000	-1.626431000	-0.326770000
H	-0.237494000	-2.675036000	-0.602813000
C	-1.288443000	-0.904453000	0.033689000
C	-1.175682000	0.445918000	0.363249000
C	0.069544000	1.054789000	0.337263000
C	1.231609000	0.347887000	-0.005800000
C	2.526041000	1.039104000	0.003253000
C	3.733490000	0.454660000	0.014783000
H	2.465582000	2.126562000	0.031827000
H	8.318931000	0.474436000	0.643997000
C	7.434582000	1.045881000	0.370966000
C	6.186198000	0.434931000	0.346649000
H	6.105945000	-0.617343000	0.607268000
C	5.029035000	1.143283000	0.005475000
C	5.174247000	2.497668000	-0.337446000
C	6.410471000	3.120417000	-0.316013000
C	7.549432000	2.395073000	0.042966000
H	3.791809000	-0.632337000	0.064718000
H	0.150732000	2.107056000	0.597865000
H	-2.071241000	0.994822000	0.633711000
O	-2.530131000	-1.460191000	0.070087000
H	-2.468248000	-2.393185000	-0.178099000
H	4.304332000	3.070102000	-0.644971000
H	6.522629000	4.165213000	-0.585309000
O	8.738600000	3.056631000	0.043425000
H	9.442106000	2.444686000	0.301444000

29 E= -459.916743359 G= -459.806531

Nimag= 0

O	-2.653299000	-0.224334000	-0.127398000
H	-1.247056000	-2.232690000	-0.837895000
C	-0.644991000	-1.385039000	-0.528285000
C	0.741775000	-1.435234000	-0.535617000
H	1.241031000	-2.343398000	-0.858174000
C	1.495597000	-0.333267000	-0.133361000
H	2.578903000	-0.387761000	-0.144445000
C	0.844437000	0.829298000	0.279108000
C	-0.551142000	0.885365000	0.288247000
C	-1.293880000	-0.217792000	-0.113621000
C	1.587601000	2.055004000	0.725025000
O	0.984296000	3.047851000	1.075695000
C	3.101248000	2.020978000	0.725974000
H	3.483404000	1.824975000	-0.280912000
H	3.469078000	1.224357000	1.380303000
H	3.471290000	2.983911000	1.077016000
H	-2.977587000	0.634132000	0.180126000
H	-1.029557000	1.805654000	0.614405000

30 E= -495.840751096 G= -495.752502

Nimag= 0

O	-2.643945000	-0.235528000	-0.102631000
H	-1.220561000	-2.233845000	-0.802917000
C	-0.624819000	-1.375997000	-0.509514000
C	0.763201000	-1.412555000	-0.530516000
H	1.267580000	-2.319704000	-0.847509000
C	1.511434000	-0.300345000	-0.149807000
H	2.594550000	-0.322409000	-0.163857000

C	0.843179000	0.853763000	0.253731000
C	-0.550863000	0.904245000	0.279481000
C	-1.284701000	-0.212866000	-0.102856000
C	1.571552000	2.081326000	0.674482000
O	1.041977000	3.106254000	1.032712000
H	-2.976979000	0.621943000	0.197955000
H	-1.036330000	1.822524000	0.600095000
O	2.910707000	1.944529000	0.619107000
H	3.281261000	2.794703000	0.911946000

31 E= -423.990721753 G= -423.857887

Nimag= 0

O	-2.289629000	1.315781000	0.335847000
H	1.877290000	-1.765066000	-0.379927000
C	1.012935000	-1.132444000	-0.208316000
C	-0.248517000	-1.701142000	-0.113246000
H	-0.362417000	-2.777931000	-0.193769000
C	-1.379801000	-0.908664000	0.073810000
H	-2.366870000	-1.359748000	0.143305000
C	-1.233140000	0.473798000	0.160046000
C	0.031694000	1.051573000	0.065487000
C	1.167273000	0.259225000	-0.109206000
C	2.481829000	0.921142000	-0.186531000
C	3.673547000	0.332867000	-0.057589000
H	2.450807000	1.999543000	-0.345113000
H	3.726302000	-0.738216000	0.134447000
C	4.982943000	1.055591000	-0.144605000
H	5.559309000	0.940763000	0.780358000
H	5.601241000	0.654398000	-0.955825000
H	4.833627000	2.123540000	-0.325393000
H	0.113485000	2.131929000	0.139185000
H	-3.103118000	0.795024000	0.388145000

32 E= -537.280305328 G= -537.141376

Nimag= 0

O	-2.343120000	1.294046000	0.051359000
H	1.916938000	-1.745321000	-0.039297000
C	1.047215000	-1.097487000	-0.020406000
C	-0.227479000	-1.651348000	-0.018286000
H	-0.345246000	-2.730314000	-0.035675000
C	-1.359467000	-0.843725000	0.005858000
H	-2.360132000	-1.261930000	0.007860000
C	-1.209890000	0.542676000	0.028270000
C	0.063936000	1.105306000	0.026228000
C	1.204484000	0.293850000	0.001921000
C	2.516816000	0.948601000	0.000889000
C	3.721787000	0.364019000	-0.020651000
C	4.953577000	1.201869000	-0.017967000
O	4.907092000	2.415470000	0.003575000
H	2.514738000	2.038560000	0.019708000
H	3.846434000	-0.715869000	-0.040558000
C	6.269327000	0.454673000	-0.043729000
H	7.096948000	1.163771000	-0.039296000
H	6.325570000	-0.176844000	-0.937133000
H	6.342496000	-0.207660000	0.825826000
H	0.178934000	2.187691000	0.043582000
H	-2.105658000	2.231890000	0.067110000

33 E= -573.204422021 G= -573.086809

Nimag= 0

O	-2.336466000	1.279947000	0.028156000
H	1.950329000	-1.722809000	-0.037026000
C	1.074867000	-1.082719000	-0.023575000
C	-0.195149000	-1.647275000	-0.020254000
H	-0.303565000	-2.727235000	-0.031305000
C	-1.333779000	-0.848992000	-0.002913000
H	-2.330835000	-1.275715000	-0.000127000
C	-1.196632000	0.538839000	0.011407000
C	0.072274000	1.112560000	0.008204000
C	1.218844000	0.309815000	-0.009353000
C	2.526068000	0.974737000	-0.012063000
C	3.730053000	0.390857000	-0.029047000
C	4.939617000	1.239530000	-0.028842000
O	4.960032000	2.448286000	-0.014321000
H	2.516016000	2.064319000	0.001097000
H	3.881330000	-0.682901000	-0.043391000
O	6.066339000	0.497357000	-0.047318000
H	6.807373000	1.127434000	-0.045717000
H	0.177862000	2.195969000	0.019339000
H	-2.108224000	2.220155000	0.037653000

34 E= -538.291627411 G= -538.139488

Nimag= 0

O	-0.299452000	-2.257845000	-6.457722000
C	-1.164246000	0.006866000	-3.745123000
C	-1.573069000	1.141952000	-4.458496000
C	-0.742925000	-1.122704000	-4.447513000
C	-1.555989000	1.135098000	-5.847379000
H	-1.930212000	2.014781000	-3.921452000
C	-0.729722000	-1.122445000	-5.841059000
H	-0.392545000	-2.008246000	-3.926917000
C	-1.135812000	0.008262000	-6.548325000
H	-1.881634000	2.013083000	-6.396729000
H	-1.123313000	0.005337000	-7.635799000
C	-1.179171000	0.001388000	-2.260514000
C	-1.562000000	-1.143306000	-1.552192000
C	-0.811240000	1.142515000	-1.538633000
H	-0.487312000	2.029841000	-2.075154000
H	-1.873674000	-2.028243000	-2.099803000
C	-0.825354000	1.139546000	-0.147815000
C	-1.576264000	-1.147283000	-0.161413000
H	-0.528504000	2.031621000	0.395322000

H	-1.883512000	-2.042309000	0.370943000
C	-1.208072000	-0.005629000	0.546102000
H	-1.219130000	-0.008430000	1.631591000
H	-0.323746000	-2.126967000	-7.415856000

35 E= -615.656587492 G= -615.475236

Nimag= 0

O	-1.146080000	-0.038631000	-7.912127000
C	-1.168785000	0.012220000	-3.739633000
C	-1.552914000	1.147734000	-4.458668000
C	-0.783498000	-1.122134000	-4.467435000
C	-1.551704000	1.157990000	-5.849132000
H	-1.883177000	2.033090000	-3.922562000
C	-0.781861000	-1.128029000	-5.854208000
H	-0.455870000	-2.009781000	-3.933437000
C	-1.166652000	0.017386000	-6.551721000
H	-1.862791000	2.049749000	-6.388989000
H	-0.475732000	-2.003462000	-6.416855000
C	-1.168734000	0.007405000	-2.257781000
C	-1.567609000	-1.126868000	-1.537187000
C	-0.770853000	1.133498000	-1.531451000
H	-0.429829000	2.018216000	-2.061854000
C	-0.771880000	1.134854000	-0.140996000
C	-1.569268000	-1.141565000	-0.150554000
H	-0.449904000	2.019247000	0.404602000
C	-1.170719000	-0.005391000	0.554523000
H	-1.429866000	0.814573000	-8.268925000
H	-1.905987000	-2.007082000	-2.076722000
H	-1.885780000	-2.016817000	0.406626000
O	-1.191637000	-0.069986000	1.914481000
H	-0.898120000	0.777502000	2.276945000

36 E= -459.928472381 G= -459.816679

Nimag= 0

O	-1.262678000	1.941679000	0.740141000
H	-1.204310000	-2.255764000	-0.809190000
C	-0.627938000	-1.389895000	-0.497139000
C	0.772067000	-1.436778000	-0.532338000
H	1.284141000	-2.331272000	-0.868716000
C	1.491279000	-0.326968000	-0.132115000
H	2.576169000	-0.357945000	-0.157457000
C	0.849514000	0.844218000	0.308090000
C	-0.564930000	0.874915000	0.337603000
C	-1.290024000	-0.254479000	-0.069714000
H	-2.372637000	-0.200504000	-0.035843000
H	-0.616008000	2.640971000	0.990547000
C	1.617246000	2.030236000	0.735850000
O	1.053492000	3.052209000	1.120654000
C	3.128454000	1.991079000	0.701332000
H	3.487975000	1.804263000	-0.315128000
H	3.510063000	1.190829000	1.342681000
H	3.505092000	2.951579000	1.051802000

37 E= -495.851710475 G= -495.762029

Nimag= 0

O	-1.278286000	1.954691000	0.717717000
H	-1.174409000	-2.271453000	-0.754832000
C	-0.606601000	-1.391574000	-0.467175000
C	0.793278000	-1.419795000	-0.521931000
H	1.312266000	-2.313610000	-0.849224000
C	1.504418000	-0.294016000	-0.153277000
H	2.588470000	-0.287374000	-0.185682000
C	0.839882000	0.867474000	0.271773000
C	-0.570076000	0.888346000	0.324279000
C	-1.282321000	-0.258899000	-0.051637000
H	-2.365004000	-0.222303000	-0.004072000
H	-0.650538000	2.672952000	0.947974000
C	1.583884000	2.070323000	0.666727000
O	1.071832000	3.113420000	1.046545000
O	2.916223000	1.949546000	0.581703000
H	3.286984000	2.802522000	0.867543000

38 E= -423.990752825 G= -423.857347

Nimag= 0

H	1.874166000	-1.710323000	-0.484858000
C	1.003195000	-1.097098000	-0.272614000
C	-0.248496000	-1.693890000	-0.166637000
H	-0.349572000	-2.768527000	-0.275429000
C	-1.369278000	-0.900956000	0.061042000
H	-2.354500000	-1.349561000	0.139854000
C	-1.231019000	0.478132000	0.178899000
H	-2.102264000	1.105696000	0.354704000
C	0.028004000	1.065202000	0.077268000
C	1.175999000	0.284095000	-0.141653000
O	0.204795000	2.413121000	0.192917000
H	-0.657612000	2.833720000	0.314956000
C	2.490813000	0.938740000	-0.239808000
C	3.664230000	0.349937000	0.006141000
H	2.476505000	1.990192000	-0.519522000
H	3.683918000	-0.688322000	0.337513000
C	4.992071000	1.031706000	-0.124631000
H	5.536418000	1.022650000	0.826511000
H	5.627428000	0.522543000	-0.858636000
H	4.871453000	2.071305000	-0.441002000

39 E= -537.280317165 G= -537.141119

Nimag= 0

O	0.182449000	2.433862000	0.183249000
H	1.923373000	-1.692146000	-0.300330000
C	1.038095000	-1.079263000	-0.160681000
C	-0.210004000	-1.683939000	-0.111928000
H	-0.298117000	-2.761374000	-0.198689000

C	-1.348180000	-0.893395000	0.038669000
H	-2.331994000	-1.350452000	0.075998000
C	-1.231586000	0.487356000	0.136104000
H	-2.117049000	1.108044000	0.252679000
C	0.026324000	1.087499000	0.086605000
C	1.190272000	0.309329000	-0.057750000
H	-0.686232000	2.853512000	0.254561000
C	2.493344000	0.974825000	-0.108092000
C	3.693836000	0.386300000	-0.005883000
C	4.935310000	1.202046000	-0.090936000
O	4.910963000	2.402826000	-0.272745000
H	2.487137000	2.055546000	-0.232394000
H	3.806803000	-0.683398000	0.155759000
C	6.239657000	0.448201000	0.065016000
H	7.077562000	1.140969000	-0.011800000
H	6.323609000	-0.323325000	-0.708255000
H	6.266253000	-0.061167000	1.034556000

40 E= -573.204751397 G= -573.087406

Nimag= 0

O	0.049249000	2.405568000	0.277485000
H	1.983810000	-1.646153000	-0.096642000
C	1.067374000	-1.068551000	-0.024970000
C	-0.153845000	-1.726711000	-0.009020000
H	-0.191488000	-2.808265000	-0.078441000
C	-1.329409000	-0.985950000	0.105213000
H	-2.292709000	-1.485885000	0.121284000
C	-1.276254000	0.398366000	0.204081000
H	-2.190861000	0.979907000	0.294234000
C	-0.044809000	1.053070000	0.186812000
C	1.154336000	0.326441000	0.066806000
H	-0.836067000	2.783503000	0.373267000
C	2.427660000	1.048475000	0.054754000
C	3.629704000	0.529515000	-0.228631000
C	4.820937000	1.399813000	-0.181145000
O	4.837399000	2.575248000	0.100971000
H	2.390091000	2.108698000	0.293614000
H	3.792015000	-0.506412000	-0.506389000
O	5.944366000	0.719782000	-0.500167000
H	6.672510000	1.362122000	-0.445216000

41 E= -538.289642038 G= -538.137416

Nimag= 0

O	-0.207887000	-2.183890000	-3.814241000
C	-1.169324000	-0.013829000	-3.730072000
C	-1.647296000	1.099288000	-4.427895000
C	-0.688223000	-1.098030000	-4.484540000
C	-1.653720000	1.154530000	-5.818024000
H	-2.040693000	1.932936000	-3.852876000
C	-0.690220000	-1.050255000	-5.877829000
C	-1.170642000	0.071638000	-6.544234000
H	-2.038847000	2.031667000	-6.326833000
H	-0.306760000	-1.899584000	-6.439589000
H	-1.167723000	0.092112000	-7.629495000
C	-1.162970000	-0.017339000	-2.244745000
C	-1.575938000	-1.134148000	-1.508204000
C	-0.772603000	1.134030000	-1.551006000
H	-0.427125000	1.998737000	-2.110999000
H	-1.879340000	-2.033817000	-2.031970000
C	-0.798327000	1.173467000	-0.160636000
C	-1.603385000	-1.093714000	-0.118573000
H	-0.485211000	2.074194000	0.358650000
H	-1.932264000	-1.966948000	0.436734000
C	-1.215680000	0.058867000	0.560995000
H	-1.235433000	0.086199000	1.646146000
H	0.139310000	-2.818315000	-4.457051000

42 E= -615.656985899 G= -615.475103

Nimag= 0

O	0.190068000	2.418688000	0.573612000
H	1.965715000	-1.504921000	-0.738173000
C	1.081466000	-0.955471000	-0.429192000
C	-0.152219000	-1.595247000	-0.412431000
H	-0.225298000	-2.642420000	-0.685902000
C	-1.292640000	-0.878173000	-0.060893000
H	-2.264506000	-1.361255000	-0.048704000
C	-1.191855000	0.469448000	0.267472000
H	-2.078662000	1.037423000	0.540515000
C	0.049592000	1.100953000	0.253247000
C	1.217726000	0.394610000	-0.087006000
H	-0.681606000	2.791679000	0.765141000
C	2.507969000	1.093708000	-0.086278000
C	3.704084000	0.495331000	0.016056000
H	2.450952000	2.177350000	-0.146268000
H	8.263351000	0.509165000	0.770971000
C	7.398841000	1.070244000	0.429373000
C	6.144580000	0.469554000	0.420641000
H	6.034294000	-0.557729000	0.759111000
C	5.009426000	1.168893000	-0.009282000
C	5.174009000	2.489381000	-0.452735000
H	4.317028000	3.042992000	-0.824110000
C	6.425365000	3.091257000	-0.442882000
H	6.531712000	4.113944000	-0.791976000
C	7.544046000	2.386187000	-0.000011000
H	8.521626000	2.858044000	0.002196000
H	3.745345000	-0.583228000	0.165560000

21an E= -495.275873189 G= -495.201401

Nimag= 0

C	-0.634329000	-1.401844000	-0.486892000
C	0.757377000	-1.441562000	-0.543551000
H	1.238670000	-2.350890000	-0.893503000

C	1.487426000	-0.322571000	-0.152792000
H	2.573171000	-0.311435000	-0.181261000
C	0.856865000	0.839075000	0.294700000
C	-0.534831000	0.857999000	0.343683000
C	-1.284144000	-0.251659000	-0.042992000
H	-2.374377000	-0.232090000	-0.002741000
C	1.682043000	2.080264000	0.727665000
O	0.999813000	3.055670000	1.107577000
H	-1.003642000	1.772526000	0.695154000
O	-1.330846000	-2.525790000	-0.880245000
H	-2.271975000	-2.328615000	-0.780391000
O	2.920298000	1.942848000	0.639709000

24an E= -572.634738063 G= -572.531428 Nimag= 0

H	1.929306000	-1.717545000	-0.036226000
C	1.050329000	-1.080236000	-0.021640000
C	-0.207668000	-1.663702000	-0.016409000
H	-0.330394000	-2.742426000	-0.026595000
C	-1.347757000	-0.859944000	0.002257000
C	-1.211175000	0.523969000	0.015469000
C	0.059331000	1.094196000	0.009982000
C	1.222983000	0.315440000	-0.008563000
C	2.538107000	0.971473000	-0.013480000
C	3.742863000	0.393668000	-0.028764000
C	5.051892000	1.215913000	-0.032091000
O	4.909340000	2.457434000	-0.019732000
H	2.545645000	2.061955000	-0.003431000
H	3.869080000	-0.690312000	-0.039693000
O	6.085957000	0.514578000	-0.047153000
H	0.157961000	2.176731000	0.020389000
H	-2.097496000	1.157274000	0.029959000
O	-2.572742000	-1.482077000	0.006746000
H	-3.253678000	-0.795916000	0.021301000

30an E= -495.27891803 G= -495.204162 Nimag= 0

H	-1.237856000	-2.253419000	-0.809534000
C	-0.639094000	-1.396112000	-0.516297000
C	0.755221000	-1.426817000	-0.535323000
H	1.259054000	-2.337552000	-0.853625000
C	1.497846000	-0.312380000	-0.153924000
H	2.583113000	-0.305256000	-0.158008000
C	0.851722000	0.855227000	0.254237000
C	-0.539757000	0.886792000	0.273595000
C	-1.279517000	-0.227833000	-0.107919000
C	1.657414000	2.114182000	0.685302000
O	0.955122000	3.089404000	1.027867000
O	-2.657717000	-0.221287000	-0.097225000
H	-2.927659000	0.656056000	0.208156000
H	-1.009092000	1.815040000	0.597952000
O	2.897183000	1.985290000	0.632701000

33an E= -572.637540747 G= -572.533996 Nimag= 0

H	1.936566000	-1.723478000	-0.036908000
C	1.058902000	-1.086040000	-0.023480000
C	-0.210243000	-1.649136000	-0.020221000
H	-0.315661000	-2.731048000	-0.031339000
C	-1.356556000	-0.856160000	-0.002890000
H	-2.354392000	-1.281795000	-0.000097000
C	-1.203851000	0.527179000	0.011309000
C	0.065164000	1.098511000	0.008065000
C	1.223598000	0.309771000	-0.009392000
C	2.533705000	0.973401000	-0.012052000
C	3.740423000	0.398680000	-0.028914000
C	5.046444000	1.227643000	-0.029686000
O	4.896469000	2.467891000	-0.013705000
H	2.537999000	2.063696000	0.000947000
H	3.869049000	-0.684604000	-0.043108000
O	6.082578000	0.530260000	-0.046429000
H	0.169550000	2.183191000	0.019203000
O	-2.340910000	1.294110000	0.028372000
H	-2.069839000	2.222590000	0.037515000

37an E= -495.307503805 G= -495.233050 Nimag= 0

H	-1.190921000	-2.289802000	-0.761213000
C	-0.619580000	-1.409022000	-0.473136000
C	0.775652000	-1.436005000	-0.526113000
H	1.297035000	-2.331634000	-0.853407000
C	1.484568000	-0.296245000	-0.152487000
H	2.570681000	-0.262552000	-0.174772000
C	0.835475000	0.858343000	0.268861000
C	-0.579013000	0.881911000	0.321625000
C	-1.293931000	-0.268426000	-0.055717000
H	-2.378935000	-0.234925000	-0.010143000
O	-1.223560000	1.974939000	0.721403000
H	-0.410402000	2.640321000	0.927113000
C	1.634151000	2.094506000	0.672942000
O	0.915562000	3.092811000	1.042391000
O	2.865347000	2.046436000	0.617431000

40an E= -572.631391216 G= -572.527644 Nimag= 0

H	1.992473000	-1.622483000	0.015262000
C	1.067492000	-1.054458000	0.041085000
C	-0.153681000	-1.722815000	0.024569000
H	-0.177080000	-2.807425000	-0.027177000
C	-1.338270000	-0.997109000	0.089635000
H	-2.301071000	-1.500045000	0.084064000
C	-1.282591000	0.392030000	0.171090000

H	-2.200174000	0.977037000	0.224565000
C	-0.054588000	1.049732000	0.180841000
C	1.161888000	0.341380000	0.106435000
O	0.014197000	2.413251000	0.256382000
H	-0.886367000	2.759781000	0.311835000
C	2.445588000	1.057785000	0.114284000
C	3.619957000	0.560404000	-0.289645000
C	4.938018000	1.363848000	-0.220501000
O	4.837627000	2.533333000	0.205955000
H	2.452420000	2.088839000	0.459650000
H	3.707604000	-0.449982000	-0.693568000
O	5.938056000	0.717356000	-0.601643000

43 E= -532.352366710 G= -532.279534

Nimag= 0

C	0.678985000	0.050537000	-2.386187000
C	-0.498389000	-0.307619000	-1.645652000
C	1.815260000	0.381340000	-1.599265000
H	2.745274000	0.663082000	-2.081797000
C	-0.550494000	-0.335683000	-0.277384000
C	1.742278000	0.346624000	-0.208219000
C	0.585843000	-0.004396000	0.484261000
H	0.539876000	-0.027592000	1.569848000
O	-1.724162000	-0.691065000	0.359415000
H	-2.346299000	-0.869136000	-0.364077000
O	-1.596591000	-0.627355000	-2.419060000
H	-1.187150000	-0.495997000	-3.313384000
O	2.893946000	0.682923000	0.492772000
H	2.673897000	0.608781000	1.430696000
O	0.582550000	0.034155000	-3.659895000

44 E= -532.355813298 G= -532.282362

Nimag= 0

C	0.558607000	-0.009490000	-2.286337000
C	-0.642615000	-0.339215000	-1.619918000
C	1.728723000	0.327868000	-1.611348000
H	2.635671000	0.576401000	-2.152883000
C	-0.598207000	-0.309306000	-0.211571000
C	1.714165000	0.340924000	-0.216938000
C	0.556134000	0.024024000	0.494889000
H	0.541225000	0.033163000	1.583975000
O	-1.779368000	-0.633835000	0.404061000
H	-2.344025000	-0.802678000	-0.385428000
O	-1.726968000	-0.652258000	-2.268684000
O	2.894410000	0.680688000	0.441218000
H	2.699056000	0.638839000	1.386260000
O	0.473709000	-0.050121000	-3.652967000
H	-0.468773000	-0.316518000	-3.760490000

45 E= -532.332839030 G= -532.262173

Nimag= 0

C	0.600350000	0.021376000	-2.238425000
C	-0.578779000	-0.279010000	-1.550483000
C	1.766633000	0.338411000	-1.570461000
H	2.660761000	0.570464000	-2.140494000
C	-0.544797000	-0.273317000	-0.162570000
C	1.846113000	0.362399000	-0.125122000
C	0.606095000	0.026842000	0.542629000
H	0.602098000	0.021491000	1.632235000
O	-1.743271000	-0.624469000	0.456834000
H	-1.545933000	-0.698156000	1.399910000
O	-1.735684000	-0.591726000	-2.280114000
H	-2.300786000	-1.078924000	-1.663211000
O	2.900777000	0.644237000	0.502118000
O	0.562711000	-0.011676000	-3.618297000
H	-0.335544000	-0.302866000	-3.840763000

46 E= -532.348945826 G= -532.276182

Nimag= 0

C	0.650127000	-0.016471000	-2.146022000
C	-0.463205000	-0.328688000	-1.388057000
C	1.870333000	0.345310000	-1.543217000
H	2.731247000	0.586071000	-2.161199000
C	-0.359870000	-0.279700000	0.011437000
C	2.012786000	0.405640000	-0.139010000
C	0.827314000	0.072290000	0.599209000
H	0.558943000	-0.057747000	-3.228928000
O	-1.483757000	-0.595253000	0.767156000
H	-1.197080000	-0.499591000	1.688848000
O	-1.674505000	-0.687799000	-1.968473000
H	-2.272331000	-0.852566000	-1.223928000
O	3.042740000	0.713618000	0.569082000
O	0.973305000	0.133156000	1.960893000
H	1.935479000	0.411835000	1.975371000

47 E= -532.361332063 G= -532.287414

Nimag= 0

C	0.656310000	-0.000524000	-2.187893000
C	-0.507398000	-0.311324000	-1.507683000
C	1.830392000	0.324983000	-1.482325000
H	2.753384000	0.571726000	-1.996381000
C	-0.567935000	-0.315749000	-0.087068000
C	1.801384000	0.328917000	-0.091040000
C	0.625398000	0.014762000	0.578160000
H	0.651449000	-0.011121000	-3.274925000
O	-1.650604000	-0.605086000	0.558194000
O	-1.690949000	-0.638764000	-2.128883000
H	-2.267973000	-0.790042000	-1.348302000
O	2.930891000	0.642363000	0.646034000
H	2.648991000	0.572939000	1.571666000
O	0.578411000	0.013731000	1.961205000
H	-0.359992000	-0.240383000	2.104976000

48 E= -532.345983626 G= -532.273495

Nimag= 0

C	0.598820000	-0.029678000	-2.263494000
C	-0.510180000	-0.343123000	-1.512931000
C	1.798909000	0.308761000	-1.609230000
C	-0.519138000	-0.348667000	-0.062745000
C	1.833706000	0.302906000	-0.230501000
C	0.708608000	-0.017175000	0.544936000
H	0.781272000	-0.029235000	1.631420000
H	0.559115000	-0.029367000	-3.348846000
O	-1.627971000	-0.657912000	0.504762000
O	-1.719554000	-0.679183000	-2.045394000
H	-2.220691000	-0.821705000	-1.195734000
O	3.063120000	0.639723000	0.357451000
H	2.854349000	1.036395000	1.213072000
O	2.924262000	0.654626000	-2.348020000
H	3.579824000	0.912284000	-1.681681000

49 E= -682.737428508 G= -682.659242

Nimag= 0

C	0.741261000	0.060213000	-2.272402000
C	-0.399910000	-0.295433000	-1.565293000
C	1.941164000	0.425028000	-1.608070000
C	-0.371363000	-0.296555000	-0.166220000
C	1.919672000	0.408198000	-0.200170000
C	0.788100000	0.055237000	0.513120000
O	-1.535332000	-0.658983000	0.514302000
H	-1.314756000	-0.592746000	1.454985000
O	-1.564713000	-0.648806000	-2.217989000
H	-2.198783000	-0.848563000	-1.512567000
O	3.088522000	0.764708000	0.446939000
H	3.662928000	0.947655000	-0.330668000
O	3.009923000	0.759297000	-2.260681000
O	0.769296000	0.040196000	1.903325000
H	1.659598000	0.317719000	2.168765000
O	0.798967000	0.087810000	-3.644077000
H	1.729848000	0.375653000	-3.772077000

Products

1 E= -306.686310713 G= -306.623196

Nimag= 0

C	0.799615000	0.004475000	-2.571996000
C	-0.367308000	-0.250990000	-1.894008000
C	1.975755000	0.312346000	-1.863132000
H	-1.293812000	-0.491755000	-2.404912000
H	2.892466000	0.513271000	-2.408071000
C	-0.409810000	-0.208942000	-0.444793000
C	1.978709000	0.363475000	-0.456758000
H	2.898800000	0.602945000	0.067107000
C	0.825003000	0.112008000	0.244990000
H	0.787856000	0.142112000	1.328994000
H	0.822951000	-0.029247000	-3.656719000
O	-1.465645000	-0.439057000	0.182761000

2 E= -460.280448837 G= -460.173080

Nimag= 0

C	2.452949000	0.717983000	0.000000000
C	2.457055000	-0.664904000	0.000000000
C	1.262029000	-1.382501000	0.000000000
C	-0.003087000	-0.717518000	0.000000000
C	-0.044381000	0.696386000	0.000000000
C	1.214469000	1.474872000	0.000000000
C	-1.273511000	1.361322000	0.000000000
C	-2.455413000	0.638629000	0.000000000
C	-2.425588000	-0.765340000	0.000000000
C	-1.218320000	-1.435527000	0.000000000
H	3.372004000	1.294116000	0.000000000
H	3.400515000	-1.201998000	0.000000000
H	1.278139000	-2.468530000	0.000000000
H	-1.266947000	2.446537000	0.000000000
H	-3.409343000	1.156460000	0.000000000
H	-3.355680000	-1.324903000	0.000000000
H	-1.190170000	-2.521955000	0.000000000
O	1.212248000	2.711100000	0.000000000

3 E= -460.272672511 G= -460.165625

Nimag= 0

C	2.447804000	0.784490000	0.000000000
C	2.439016000	-0.682268000	0.000000000
C	1.278052000	-1.371890000	0.000000000
C	-0.002597000	-0.702511000	0.000000000
C	-0.043241000	0.725469000	0.000000000
C	1.164820000	1.444808000	0.000000000
C	-1.304071000	1.378614000	0.000000000
C	-2.471841000	0.651048000	0.000000000
C	-2.424512000	-0.756351000	0.000000000
C	-1.208243000	-1.417782000	0.000000000
H	3.406476000	-1.173388000	0.000000000
H	1.278642000	-2.459272000	0.000000000
H	1.168608000	2.530735000	0.000000000
H	-1.327261000	2.464842000	0.000000000
H	-3.430939000	1.158433000	0.000000000
H	-3.348560000	-1.325634000	0.000000000
H	-1.176706000	-2.503981000	0.000000000
O	3.507513000	1.434129000	0.000000000

4 E= -613.873596062 G= -613.722033

Nimag= 0

C	3.688479000	0.586128000	0.000000000
C	3.648086000	-0.818279000	0.000000000
C	2.428454000	-1.477099000	0.000000000

C	1.234647000	-0.752813000	0.000000000
C	2.518541000	1.317309000	0.000000000
C	1.262174000	0.665811000	0.000000000
C	-0.054125000	-1.482177000	0.000000000
C	-1.287709000	-0.663173000	0.000000000
C	0.048157000	1.399112000	0.000000000
C	-1.214826000	0.753840000	0.000000000
C	-2.529662000	-1.301428000	0.000000000
C	-3.699689000	-0.558179000	0.000000000
C	-3.640706000	0.845566000	0.000000000
C	-2.422023000	1.492423000	0.000000000
H	4.645761000	1.098046000	0.000000000
H	4.573465000	-1.385165000	0.000000000
H	2.365107000	-2.560497000	0.000000000
H	2.546853000	2.403650000	0.000000000
H	0.086702000	2.485173000	0.000000000
H	-2.542991000	-2.386634000	0.000000000
H	-4.662901000	-1.058090000	0.000000000
H	-4.559300000	1.424048000	0.000000000
H	-2.373642000	2.578054000	0.000000000
O	-0.097968000	-2.707924000	0.000000000

5 E= -613.861372913 G= -613.710291
Nimag= 0

C	3.695508000	0.602235000	0.007584000
C	3.681575000	-0.781091000	0.018527000
C	2.437549000	-1.534386000	0.023054000
C	1.180312000	-0.740829000	0.014584000
C	2.512097000	1.339167000	0.000083000
C	1.236133000	0.692960000	0.003246000
C	-0.032470000	-1.385433000	0.017712000
C	-1.247241000	-0.660398000	0.009936000
C	0.048350000	1.414956000	-0.004412000
C	-1.203184000	0.767658000	-0.001320000
C	-2.509691000	-1.310346000	0.013016000
C	-3.671020000	-0.582727000	0.005288000
C	-3.628357000	0.835556000	-0.005918000
C	-2.427465000	1.493296000	-0.009140000
H	4.645317000	1.128435000	0.004710000
H	4.597367000	-1.362139000	0.024343000
H	2.544827000	2.424525000	-0.008369000
H	-0.041179000	-2.472299000	0.026379000
H	0.081057000	2.502417000	-0.012974000
H	-2.534729000	-2.396492000	0.021603000
H	-4.631248000	-1.088532000	0.007717000
H	-4.556680000	1.397989000	-0.011985000
H	-2.390019000	2.579331000	-0.017717000
O	2.425558000	-2.765960000	0.033246000

6 E= -613.85354124 G= -613.702796
Nimag= 0

C	3.688087000	0.616944000	0.000000000
C	3.688456000	-0.857615000	0.000000000
C	2.394980000	-1.506879000	0.000000000
C	1.189506000	-0.778030000	0.000000000
C	2.539109000	1.317803000	0.000000000
C	1.240770000	0.666793000	0.000000000
C	-0.057154000	-1.413390000	0.000000000
C	-1.257371000	-0.681187000	0.000000000
C	0.067868000	1.384547000	0.000000000
C	-1.198173000	0.746936000	0.000000000
C	-2.530059000	-1.318350000	0.000000000
C	-3.681970000	-0.577741000	0.000000000
C	-3.621161000	0.839117000	0.000000000
C	-2.409684000	1.483237000	0.000000000
H	4.660930000	1.097613000	0.000000000
H	2.388563000	-2.592684000	0.000000000
H	2.552250000	2.405336000	0.000000000
H	-0.097006000	-2.500349000	0.000000000
H	0.104573000	2.472083000	0.000000000
H	-2.567943000	-2.404394000	0.000000000
H	-4.648361000	-1.071603000	0.000000000
H	-4.542098000	1.413517000	0.000000000
H	-2.360503000	2.568850000	0.000000000
O	4.739244000	-1.509590000	0.000000000

7 E= -767.454977614 G= -767.259638
Nimag= 0

C	-1.304195000	0.571873000	0.010102000
C	-1.314326000	-0.867034000	0.010214000
C	-2.503153000	-1.551604000	0.007413000
C	-3.745802000	-0.871840000	0.004328000
C	-3.750922000	0.557021000	0.004171000
C	-2.524093000	1.248323000	0.007083000
C	-5.000914000	1.237131000	0.001064000
C	-6.177531000	0.535676000	-0.001709000
C	-6.170175000	-0.882546000	-0.001549000
C	-4.981689000	-1.567270000	0.001391000
H	-2.473568000	-2.638213000	0.007597000
H	-2.530477000	2.336156000	0.006966000
H	-5.003325000	2.323832000	0.000943000
H	-7.125403000	1.064670000	-0.004075000
H	-7.111336000	-1.422835000	-0.003792000
H	-4.966743000	-2.653741000	0.001530000
C	2.403970000	1.283056000	0.019054000
C	-0.066568000	1.263891000	0.013048000
C	3.629332000	-0.799625000	0.022174000
C	1.176749000	0.578887000	0.016125000
C	-0.039126000	-1.631065000	0.013378000
C	1.214348000	-0.840087000	0.016332000
C	3.605929000	0.604307000	0.022013000
C	2.438403000	-1.510183000	0.019350000

H	4.578514000	-1.325451000	0.024525000
H	2.423028000	-2.595445000	0.019403000
H	4.538870000	1.159498000	0.024247000
H	2.385040000	2.369589000	0.018929000
H	-0.070184000	2.350501000	0.012950000
O	-0.026004000	-2.854310000	0.013533000

8 E= -767.436970762 G= -767.242313

Nimag= 0

C	-1.302679000	0.581582000	0.010089000
C	-1.294326000	-0.858635000	0.010245000
C	-2.506432000	-1.548733000	0.007380000
C	-3.731148000	-0.874293000	0.004356000
C	-3.741461000	0.562476000	0.004194000
C	-2.529440000	1.255279000	0.007059000
C	-5.001789000	1.239782000	0.001080000
C	-6.170925000	0.538695000	-0.001671000
C	-6.160045000	-0.888860000	-0.001508000
C	-4.979292000	-1.571131000	0.001402000
H	-2.496796000	-2.636422000	0.007507000
H	-2.538292000	2.343151000	0.006935000
H	-5.004632000	2.326481000	0.000964000
H	-7.121437000	1.062757000	-0.004033000
H	-7.102138000	-1.427757000	-0.003750000
H	-4.964467000	-2.657688000	0.001535000
C	2.394916000	1.282306000	0.019011000
C	-0.063218000	1.272875000	0.013033000
C	3.643445000	-0.798013000	0.022247000
C	1.144170000	0.595312000	0.016015000
C	-0.039359000	-1.537203000	0.013346000
C	1.140328000	-0.850311000	0.016132000
C	3.603901000	0.583170000	0.022034000
C	2.428031000	-1.599461000	0.019341000
H	4.581189000	-1.342828000	0.024604000
H	4.534394000	1.143310000	0.024281000
H	2.392379000	2.368092000	0.018915000
H	-0.069959000	2.360715000	0.012925000
H	-0.007851000	-2.623799000	0.013529000
O	2.460324000	-2.828718000	0.019512000

9 E= -767.430176639 G= -767.235834

Nimag= 0

C	-1.295405000	0.567053000	0.010158000
C	-1.289301000	-0.874389000	0.010298000
C	-2.508315000	-1.561376000	0.007393000
C	-3.730170000	-0.881399000	0.004362000
C	-3.733278000	0.553632000	0.004236000
C	-2.511010000	1.241782000	0.007153000
C	-4.985356000	1.237486000	0.001125000
C	-6.161186000	0.542782000	-0.001675000
C	-6.158902000	-0.882539000	-0.001552000
C	-4.981095000	-1.571943000	0.001367000
H	-2.504262000	-2.649182000	0.007496000
H	-2.516384000	2.329694000	0.007048000
H	-4.982338000	2.324151000	0.001036000
H	-7.108002000	1.073324000	-0.004042000
H	-7.103860000	-1.416336000	-0.003827000
H	-4.972564000	-2.658570000	0.001470000
C	2.422060000	1.301944000	0.019050000
C	-0.042577000	1.258728000	0.013125000
C	3.667599000	-0.823625000	0.022278000
C	1.151481000	0.595606000	0.016062000
C	-0.046143000	-1.551112000	0.013377000
C	1.168916000	-0.860867000	0.016250000
C	3.599476000	0.652167000	0.021957000
C	2.400879000	-1.530499000	0.019298000
H	2.443519000	-2.615456000	0.019509000
H	4.549938000	1.175638000	0.024205000
H	2.387624000	2.389049000	0.018858000
H	-0.054385000	2.346763000	0.012995000
H	-0.037670000	-2.638436000	0.013501000
O	4.745110000	-1.422914000	0.024923000

10 E= -381.897528871 G= -381.829743

Nimag= 0

C	0.699475000	-0.006038000	-2.271685000
C	-0.449457000	-0.364282000	-1.573577000
C	1.826497000	0.346669000	-1.546433000
H	2.730869000	0.628648000	-2.075770000
C	-0.489960000	-0.375607000	-0.109669000
C	1.831663000	0.349673000	-0.124268000
H	2.740537000	0.634103000	0.395976000
C	0.714682000	0.001432000	0.584052000
H	0.689846000	-0.005251000	1.668358000
H	0.691179000	-0.009626000	-3.355871000
O	-1.566539000	-0.711451000	0.431181000
O	-1.572962000	-0.715819000	-2.182250000
H	-2.201930000	-0.911740000	-1.450877000

11 E= -381.877517207 G= -381.812006

Nimag= 0

C	0.698290000	0.036636000	-2.293501000
C	-0.492686000	-0.308688000	-1.551779000
C	1.892846000	0.372313000	-1.539396000
H	-1.391293000	-0.561507000	-2.102863000
H	2.780501000	0.629797000	-2.106365000
C	-0.482663000	-0.315524000	-0.176334000
C	1.872405000	0.356806000	-0.166428000
H	2.761820000	0.606765000	0.402696000
C	0.697132000	0.016430000	0.523418000
H	0.693093000	0.007658000	1.611026000
O	-1.627856000	-0.646962000	0.475991000

H	-1.482379000	-0.612933000	1.431645000
O	0.704831000	0.047200000	-3.543797000

12 E= -381.885821445 G= -381.819245

Nimag= 0

C	0.681134000	-0.006384000	-2.258565000
C	-0.454025000	-0.354782000	-1.574901000
C	1.846499000	0.351163000	-1.548499000
H	-1.365805000	-0.634616000	-2.091865000
C	-0.490934000	-0.366309000	-0.123947000
C	1.862581000	0.355931000	-0.135422000
H	2.784327000	0.638783000	0.362311000
C	0.735999000	0.010197000	0.557033000
H	0.715155000	0.003787000	1.641762000
H	0.697881000	-0.001155000	-3.346620000
O	-1.521838000	-0.682873000	0.502104000
O	2.987700000	0.701497000	-2.170392000
H	2.865803000	0.664651000	-3.130815000

13a E= -457.095336969 G= -457.024514

Nimag= 0

C	0.765187000	0.033572000	-2.326395000
C	-0.402730000	-0.262252000	-1.674682000
C	1.924605000	0.395442000	-1.592489000
H	-1.302757000	-0.541575000	-2.211165000
C	-0.471398000	-0.210705000	-0.240483000
C	1.924264000	0.464127000	-0.195620000
H	2.824584000	0.743450000	0.338146000
C	0.753958000	0.168322000	0.468699000
H	0.819087000	-0.004762000	-3.411893000
O	-1.486353000	-0.461595000	0.448571000
O	3.081391000	0.689720000	-2.216803000
H	2.968311000	0.610653000	-3.175342000
O	0.655224000	0.205944000	1.793013000
H	-0.277290000	-0.046310000	1.977917000

13b E= -457.089185604 G= -457.018898

Nimag= 0

C	0.768989000	0.041051000	-2.295996000
C	-0.402901000	-0.262359000	-1.632001000
C	1.958188000	0.401779000	-1.584776000
H	-1.305769000	-0.536063000	-2.165771000
C	-0.407493000	-0.210715000	-0.239510000
C	1.982003000	0.460583000	-0.218117000
H	2.884760000	0.734235000	0.314308000
C	0.794481000	0.155620000	0.525181000
H	0.796878000	0.007622000	-3.382424000
O	-1.481405000	-0.484539000	0.483753000
H	-1.180243000	-0.364845000	1.415703000
O	3.094366000	0.694644000	-2.266577000
H	2.938233000	0.613919000	-3.217629000
O	0.678748000	0.170288000	1.767251000

13c E= -457.083230006 G= -457.013449

Nimag= 0

C	0.763387000	0.038399000	-2.325936000
C	-0.375158000	-0.254144000	-1.620402000
C	2.001109000	0.411221000	-1.657992000
H	-1.303194000	-0.533198000	-2.107844000
C	-0.361799000	-0.196985000	-0.217462000
C	1.972144000	0.458406000	-0.203519000
H	2.890899000	0.735716000	0.305379000
C	0.830816000	0.164148000	0.477123000
H	0.785722000	0.003416000	-3.409496000
O	-1.471213000	-0.481781000	0.476250000
H	-1.271686000	-0.390442000	1.424499000
O	3.033550000	0.676980000	-2.296993000
O	0.677154000	0.172690000	1.834932000
H	1.509444000	0.423223000	2.260334000

14 E= -457.071321011 G= -457.002957

Nimag= 0

C	0.776269000	0.042672000	-2.286353000
C	-0.405106000	-0.262074000	-1.569563000
C	1.951563000	0.399016000	-1.609497000
C	-0.423750000	-0.214822000	-0.200606000
C	1.969400000	0.456842000	-0.230619000
H	2.869497000	0.730530000	0.306706000
C	0.775677000	0.150274000	0.526830000
H	0.743860000	-0.007884000	-3.371345000
O	3.095279000	0.694968000	-2.278830000
H	2.946977000	0.616422000	-3.231585000
O	0.781015000	0.199560000	1.774783000
H	-1.312362000	-0.442491000	0.380077000
O	-1.475345000	-0.592418000	-2.336509000
H	-2.231117000	-0.781987000	-1.762006000

15a E= -457.101510678 G= -457.030062

Nimag= 0

C	0.621050000	-0.013300000	-2.192057000
C	-0.532471000	-0.327641000	-1.502164000
C	1.772724000	0.312535000	-1.461981000
H	2.676862000	0.559027000	-2.009598000
C	-0.551741000	-0.320689000	-0.053148000
C	1.817118000	0.336544000	-0.060601000
H	2.724911000	0.593065000	0.472162000
C	0.671537000	0.024495000	0.642962000
H	0.621364000	-0.022310000	-3.275417000
O	-1.598428000	-0.605946000	0.580983000
O	-1.678122000	-0.651055000	-2.104797000
H	-2.319567000	-0.823236000	-1.385771000
O	0.612145000	0.018870000	1.976025000

H	-0.305965000	-0.233823000	2.202386000

15b E= -457.089301622 G= -457.018927 Nimag= 0

C	0.641636000	-0.014663000	-2.165401000
C	-0.514735000	-0.343908000	-1.481596000
C	1.833288000	0.331047000	-1.467176000
H	2.712252000	0.581137000	-2.052184000
C	-0.481275000	-0.328055000	-0.085691000
C	1.893177000	0.354382000	-0.102186000
H	2.793415000	0.615740000	0.441687000
C	0.716615000	0.019492000	0.662075000
H	0.619537000	-0.025856000	-3.249644000
O	-1.574643000	-0.639093000	0.613218000
H	-1.300557000	-0.556282000	1.554360000
O	-1.649257000	-0.672866000	-2.148091000
H	-2.340074000	-0.868889000	-1.495307000
O	0.627426000	-0.000766000	1.910137000

16a E= -532.300167962 G= -532.225395 Nimag= 0

C	0.616955000	0.033760000	-2.304933000
C	-0.599762000	-0.328252000	-1.609542000
C	1.759129000	0.365701000	-1.616278000
H	2.673116000	0.637833000	-2.128921000
C	-0.572083000	-0.328691000	-0.164160000
C	1.722473000	0.346818000	-0.209301000
C	0.572139000	0.003996000	0.525864000
H	0.576349000	-0.001132000	1.611196000
O	-1.709679000	-0.667251000	0.446253000
H	-2.347623000	-0.850877000	-0.273936000
O	-1.646979000	-0.632652000	-2.238413000
O	2.868872000	0.680644000	0.413373000
H	2.745441000	0.639751000	1.373099000
O	0.553904000	0.022896000	-3.638699000
H	-0.361940000	-0.244926000	-3.857147000

16b E= -532.284418026 G= -532.210880 Nimag= 0

C	0.605709000	0.010669000	-2.294411000
C	-0.551973000	-0.329717000	-1.574727000
C	1.735569000	0.344469000	-1.587782000
H	2.649786000	0.613432000	-2.103104000
C	-0.598135000	-0.341630000	-0.115089000
C	1.718435000	0.341289000	-0.154169000
C	0.607386000	0.014622000	0.576053000
H	0.601247000	0.014206000	1.660866000
O	-1.690455000	-0.662898000	0.395669000
O	-1.669069000	-0.659669000	-2.217780000
H	-2.313713000	-0.849126000	-1.494987000
O	2.900613000	0.690768000	0.404614000
H	2.816284000	0.668399000	1.369148000
O	0.600078000	0.007465000	-3.647646000
H	-0.284603000	-0.254440000	-3.948963000

16c E= -532.275525634 G= -532.203127 Nimag= 0

C	0.617519000	0.014314000	-2.274648000
C	-0.540007000	-0.326045000	-1.533962000
C	1.765818000	0.353499000	-1.612610000
H	2.662686000	0.617237000	-2.160177000
C	-0.529268000	-0.321165000	-0.118521000
C	1.816056000	0.370077000	-0.160499000
C	0.602017000	0.013130000	0.564397000
H	0.643627000	0.026704000	1.649011000
O	-1.733631000	-0.675679000	0.422572000
H	-1.676995000	-0.660935000	1.388447000
O	-1.649818000	-0.654311000	-2.223358000
H	-2.357104000	-0.862438000	-1.588069000
O	2.852980000	0.676479000	0.450805000
O	0.557000000	-0.005219000	-3.626784000
H	-0.337561000	-0.269813000	-3.894350000

17a E= -532.290138744 G= -532.216651 Nimag= 0

C	0.724679000	0.004740000	-2.247366000
C	-0.429508000	-0.320212000	-1.495727000
C	1.890173000	0.349037000	-1.615733000
H	2.787945000	0.602564000	-2.167707000
C	-0.411408000	-0.298304000	-0.090581000
C	1.961073000	0.386035000	-0.175124000
C	0.748338000	0.044378000	0.554435000
H	0.644050000	-0.031052000	-3.328141000
O	-1.575707000	-0.627451000	0.546084000
H	-1.427133000	-0.573835000	1.503389000
O	-1.558372000	-0.654100000	-2.136818000
H	-2.242157000	-0.844435000	-1.470632000
O	2.970283000	0.686389000	0.500756000
O	0.802367000	0.075555000	1.894840000
H	1.725960000	0.343448000	2.094922000

17b E= -532.291700228 G= -532.217938 Nimag= 0

C	0.708640000	0.001882000	-2.126339000
C	-0.472190000	-0.338545000	-1.430329000
C	1.890898000	0.344559000	-1.471043000
H	2.785563000	0.602514000	-2.024459000
C	-0.468240000	-0.335483000	-0.056565000
C	1.910147000	0.351970000	-0.085738000
C	0.718295000	0.008442000	0.675611000
H	0.665151000	-0.012136000	-3.210238000
O	-1.583525000	-0.656647000	0.635793000

H	-1.344414000	-0.586544000	1.579861000
O	-1.583401000	-0.660782000	-2.142474000
H	-2.293046000	-0.865214000	-1.512466000
O	2.990610000	0.665286000	0.628060000
H	2.719561000	0.588368000	1.566621000
O	0.737829000	0.015834000	1.930556000

18 E= -532.28954931 G= -532.215712

Nimag= 0

C	0.688180000	0.020028000	-2.277723000
C	-0.455962000	-0.318475000	-1.573254000
C	1.820566000	0.359228000	-1.551929000
C	-0.493934000	-0.325366000	-0.105971000
C	1.833310000	0.367273000	-0.116780000
C	0.710461000	0.035132000	0.586438000
H	0.716603000	0.040245000	1.669646000
H	0.686688000	0.016454000	-3.363397000
O	-1.578536000	-0.646549000	0.423998000
O	-1.589426000	-0.657598000	-2.174717000
H	-2.207350000	-0.839177000	-1.428081000
O	2.977075000	0.709672000	0.516775000
H	3.653336000	0.910165000	-0.149837000
O	3.004607000	0.710364000	-2.112359000
H	2.941232000	0.688968000	-3.078258000

19 E= -682.67800981 G= -682.598626

Nimag= 0

C	0.743178000	0.035381000	-2.311774000
C	-0.472547000	-0.309628000	-1.615976000
C	1.872653000	0.361454000	-1.613809000
C	-0.441697000	-0.295743000	-0.174929000
C	1.861287000	0.363460000	-0.198468000
C	0.693129000	0.032015000	0.525998000
O	-1.575082000	-0.618135000	0.486619000
H	-2.237903000	-0.810656000	-0.205126000
O	-1.515913000	-0.611956000	-2.245527000
O	2.970626000	0.683359000	0.474089000
H	3.668599000	0.880856000	-0.175280000
O	3.063190000	0.700154000	-2.195528000
H	2.953774000	0.664789000	-3.158868000
O	0.743736000	0.051666000	1.881146000
H	-0.135891000	-0.197575000	2.207481000
O	0.737007000	0.028501000	-3.661755000
H	-0.170483000	-0.231839000	-3.916193000

20 E= -459.271545134 G= -459.175534

Nimag= 0

C	-0.673495000	-1.444928000	-0.502392000
C	0.780290000	-1.458785000	-0.539771000
H	1.253338000	-2.370494000	-0.889122000
C	1.504991000	-0.362943000	-0.148510000
H	2.589563000	-0.392891000	-0.183787000
C	0.848422000	0.805512000	0.300302000
C	-0.565836000	0.854251000	0.349745000
C	-1.312346000	-0.224845000	-0.034751000
H	-2.397071000	-0.218081000	-0.008244000
C	1.602663000	2.023590000	0.736369000
O	0.998257000	3.006701000	1.114886000
C	3.115128000	1.998690000	0.694065000
H	3.472951000	1.818600000	-0.324799000
H	3.509095000	1.200024000	1.330811000
H	3.486474000	2.960934000	1.045309000
H	-1.025703000	1.771936000	0.701026000
O	-1.337744000	-2.436210000	-0.856037000

21 E= -495.195072425 G= -495.121037

Nimag= 0

C	-0.652725000	-1.432099000	-0.495451000
C	0.799854000	-1.432034000	-0.544239000
H	1.279327000	-2.340307000	-0.893545000
C	1.518217000	-0.328158000	-0.163396000
H	2.601805000	-0.325053000	-0.198640000
C	0.841468000	0.827353000	0.283133000
C	-0.568031000	0.871714000	0.346807000
C	-1.304793000	-0.219870000	-0.028858000
H	-2.389023000	-0.226078000	0.005203000
C	1.581382000	2.047200000	0.705468000
O	1.054118000	3.061038000	1.094917000
H	-1.035461000	1.785708000	0.697744000
O	-1.311574000	-2.431798000	-0.840041000
O	2.916885000	1.909418000	0.610880000
H	3.303274000	2.751840000	0.906792000

22 E= -423.353806220 G= -423.234454

Nimag= 0

H	1.900176000	-1.788571000	-0.120689000
C	1.019615000	-1.155326000	-0.076199000
C	-0.216713000	-1.727725000	-0.042372000
H	-0.349918000	-2.804658000	-0.058687000
C	-1.429067000	-0.919922000	0.017619000
C	-1.240374000	0.522978000	0.038514000
C	0.010233000	1.070366000	0.003496000
C	1.178359000	0.261331000	-0.054405000
C	2.471839000	0.912975000	-0.088579000
C	3.676291000	0.314321000	-0.141532000
H	2.446407000	2.002571000	-0.068253000
H	3.741701000	-0.771951000	-0.162601000
C	4.975408000	1.052936000	-0.174474000
H	5.604724000	0.774059000	0.678496000
H	5.542521000	0.804855000	-1.079024000
H	4.820356000	2.134504000	-0.150052000
H	0.133884000	2.150449000	0.019862000

O	-2.557398000	-1.435677000	0.048961000
H	-2.135538000	1.134655000	0.082865000

23 E= -536.641009075 G= -536.515432

Nimag= 0

H	1.952992000	-1.748406000	-0.041590000
C	1.066244000	-1.122366000	-0.031363000
C	-0.166731000	-1.698456000	-0.035690000
H	-0.299825000	-2.775346000	-0.049217000
C	-1.383313000	-0.889111000	-0.022198000
C	-1.202575000	0.557842000	-0.003775000
H	-2.102023000	1.164480000	0.006428000
C	0.045511000	1.108457000	0.000178000
C	1.215803000	0.297407000	-0.013448000
C	2.502768000	0.947136000	-0.008614000
C	3.716661000	0.355326000	-0.020137000
C	4.948927000	1.192150000	-0.012775000
O	4.891407000	2.405442000	0.002615000
H	2.503877000	2.036621000	0.005783000
H	3.839552000	-0.724517000	-0.034863000
C	6.265721000	0.449395000	-0.027548000
H	7.090223000	1.161909000	-0.020727000
H	6.330704000	-0.184698000	-0.918485000
H	6.336858000	-0.208864000	0.845199000
H	0.169863000	2.188099000	0.013895000
O	-2.507877000	-1.406754000	-0.026088000

24 E= -572.564443869 G= -572.460227

Nimag= 0

H	1.969712000	-1.722240000	-0.035154000
C	1.079396000	-1.101538000	-0.021207000
C	-0.151250000	-1.684546000	-0.016468000
H	-0.277816000	-2.762139000	-0.026304000
C	-1.370598000	-0.881542000	0.002247000
C	-1.198787000	0.565650000	0.015509000
C	0.046968000	1.123799000	0.010362000
C	1.218904000	0.317958000	-0.008009000
C	2.505619000	0.974937000	-0.012480000
C	3.715126000	0.381160000	-0.029071000
C	4.928905000	1.225594000	-0.030921000
O	4.944763000	2.433799000	-0.018657000
H	2.501057000	2.064109000	-0.001310000
H	3.862507000	-0.692996000	-0.041366000
O	6.050097000	0.479065000	-0.048242000
H	6.797640000	1.101790000	-0.048276000
H	0.165115000	2.204125000	0.020340000
H	-2.101521000	1.167194000	0.029491000
O	-2.493278000	-1.405489000	0.006640000

25 E= -537.651298664 G= -537.512803

Nimag= 0

C	-1.174701000	0.006177000	-3.742149000
C	-1.509540000	1.180441000	-4.464949000
C	-0.829784000	-1.164677000	-4.465716000
C	-1.507322000	1.195999000	-5.833135000
H	-1.805808000	2.068007000	-3.913837000
C	-0.812694000	-1.173763000	-5.833868000
H	-0.541302000	-2.054861000	-3.914687000
C	-1.154495000	0.012962000	-6.598642000
H	-1.778192000	2.082621000	-6.397115000
H	-0.533946000	-2.057765000	-6.398121000
C	-1.184936000	0.002724000	-2.270321000
C	-1.535369000	-1.153756000	-1.557027000
C	-0.844388000	1.155818000	-1.546840000
H	-0.540046000	2.051995000	-2.079109000
H	-1.832336000	-2.047413000	-2.097634000
C	-0.850350000	1.150642000	-0.157580000
C	-1.548469000	-1.155114000	-0.167805000
H	-0.571342000	2.048647000	0.384566000
H	-1.834840000	-2.055660000	0.366229000
C	-1.204208000	-0.003893000	0.537368000
H	-1.211631000	-0.006450000	1.622699000
O	-1.145793000	0.015910000	-7.843865000

26 E= -612.845556634 G= -612.704180

Nimag= 0

C	-1.167737000	0.006924000	-3.736404000
C	-1.480166000	1.188033000	-4.460890000
C	-0.855077000	-1.170926000	-4.465907000
C	-1.485486000	1.203933000	-5.828279000
H	-1.756550000	2.082715000	-3.910881000
C	-0.847586000	-1.180109000	-5.833456000
H	-0.580706000	-2.068556000	-3.919804000
C	-1.165558000	0.013801000	-6.597960000
H	-1.740716000	2.096915000	-6.389621000
H	-0.592170000	-2.070664000	-6.398535000
C	-1.167161000	0.003616000	-2.269285000
C	-1.489740000	-1.156951000	-1.542808000
C	-0.843564000	1.158208000	-1.541043000
H	-0.557565000	2.063680000	-2.067170000
C	-0.836014000	1.158621000	-0.153901000
C	-1.494456000	-1.166820000	-0.159320000
H	-0.566097000	2.060646000	0.390142000
C	-1.164029000	-0.005255000	0.543709000
O	-1.164245000	0.017032000	-7.842658000
H	-1.776272000	-2.058925000	-2.074710000
H	-1.758561000	-2.057428000	0.400284000
O	-1.179742000	-0.070713000	1.897718000
H	-0.940185000	0.791368000	2.266644000

27 E= -615.020614405 G= -614.853360

Nimag= 0

H	2.023847000	-1.688491000	0.003531000
C	1.124543000	-1.080755000	0.009704000
C	-0.092480000	-1.689396000	0.015231000
H	-0.192216000	-2.770062000	0.013674000
C	-1.333140000	-0.918977000	0.023529000
C	-1.188754000	0.531607000	0.025576000
C	0.042591000	1.116149000	0.019928000
C	1.241142000	0.343594000	0.011854000
C	2.502507000	1.033538000	0.006369000
C	3.731511000	0.460186000	-0.000967000
H	2.431114000	2.119448000	0.008645000
H	8.337832000	0.381725000	-0.021707000
C	7.441991000	0.994512000	-0.017447000
C	6.190975000	0.390069000	-0.012145000
H	6.114167000	-0.694227000	-0.012268000
C	5.014791000	1.155744000	-0.006512000
C	5.134530000	2.555939000	-0.006517000
H	4.245890000	3.178973000	-0.002503000
C	6.383412000	3.159408000	-0.011818000
H	6.456028000	4.242475000	-0.011759000
C	7.542935000	2.382965000	-0.017280000
H	8.517811000	2.859977000	-0.021426000
H	3.805920000	-0.625329000	-0.002903000
H	0.132409000	2.199804000	0.021552000
H	-2.103336000	1.115453000	0.031768000
O	-2.442912000	-1.468579000	0.028539000

28 E= -690.21498638 G= -690.044518
Nimag= 0

H	2.025508000	-1.685912000	0.014128000
C	1.125809000	-1.078526000	0.010479000
C	-0.090368000	-1.688314000	0.002942000
H	-0.188540000	-2.769172000	0.000494000
C	-1.332583000	-0.920080000	-0.002336000
C	-1.188828000	0.530698000	0.001156000
C	0.041469000	1.116584000	0.008718000
C	1.242350000	0.346356000	0.013647000
C	2.500872000	1.036698000	0.021456000
C	3.732496000	0.464342000	0.026854000
H	2.428715000	2.122674000	0.022931000
H	8.343541000	0.384748000	0.048839000
C	7.443695000	0.995114000	0.045953000
C	6.191905000	0.396749000	0.038608000
H	6.122881000	-0.687891000	0.035773000
C	5.012398000	1.155839000	0.034680000
C	5.140629000	2.558452000	0.038589000
C	6.380556000	3.167421000	0.045920000
C	7.542232000	2.386501000	0.049609000
H	3.805508000	-0.621440000	0.025235000
H	0.129646000	2.200463000	0.011152000
H	-2.103884000	1.113903000	-0.002603000
O	-2.441527000	-1.471117000	-0.009397000
H	4.254327000	3.184735000	0.035931000
H	6.481484000	4.247259000	0.048952000
O	8.729177000	3.041309000	0.056747000
H	9.453336000	2.399187000	0.058591000

29 E= -459.26918229 G= -459.173274
Nimag= 0

H	-1.232693000	-2.278770000	-0.820152000
C	-0.631888000	-1.428969000	-0.513327000
C	0.740449000	-1.453046000	-0.544307000
H	1.267570000	-2.339307000	-0.882669000
C	1.478113000	-0.325987000	-0.136602000
H	2.562170000	-0.367247000	-0.169334000
C	0.832413000	0.842537000	0.308792000
C	-0.548024000	0.886256000	0.347333000
C	-1.343619000	-0.249106000	-0.062569000
C	1.596129000	2.063525000	0.751101000
O	1.001560000	3.048477000	1.128990000
C	3.108434000	2.023066000	0.710010000
H	3.464240000	1.836947000	-0.308676000
H	3.492233000	1.220361000	1.348297000
H	3.491139000	2.981221000	1.060212000
O	-2.593817000	-0.215308000	-0.030003000
H	-1.058818000	1.780707000	0.688555000

30 E= -495.193994061 G= -495.119950
Nimag= 0

H	-1.206285000	-2.269602000	-0.815653000
C	-0.612917000	-1.410083000	-0.521456000
C	0.760969000	-1.429922000	-0.536338000
H	1.293824000	-2.322287000	-0.848374000
C	1.491495000	-0.293926000	-0.147353000
H	2.575270000	-0.308635000	-0.158882000
C	0.823819000	0.876506000	0.261706000
C	-0.553001000	0.924910000	0.286681000
C	-1.338189000	-0.225403000	-0.106975000
C	1.574263000	2.096911000	0.679623000
O	1.060440000	3.125665000	1.039711000
O	-2.587702000	-0.197757000	-0.089979000
H	-1.072117000	1.824389000	0.601141000
O	2.909432000	1.931348000	0.614544000
H	3.304921000	2.771982000	0.903549000

31 E= -423.347356507 G= -423.228685
Nimag= 0

H	1.838467000	-1.740973000	-0.503342000
C	0.982591000	-1.112602000	-0.279591000
C	-0.280577000	-1.708003000	-0.152011000
H	-0.371368000	-2.784165000	-0.261841000
C	-1.392780000	-0.938785000	0.099242000

H	-2.385145000	-1.365174000	0.200110000
C	-1.273009000	0.499419000	0.230266000
C	0.051596000	1.068000000	0.092871000
C	1.163621000	0.288366000	-0.145950000
C	2.487880000	0.923828000	-0.263159000
C	3.671057000	0.305038000	-0.253300000
H	2.469866000	2.010412000	-0.345544000
H	3.716037000	-0.778012000	-0.143637000
C	4.988025000	1.008650000	-0.371142000
H	5.615711000	0.816138000	0.506012000
H	5.545398000	0.653853000	-1.245330000
H	4.852302000	2.089089000	-0.466561000
H	0.130073000	2.146857000	0.193903000
O	-2.271128000	1.217991000	0.452310000

32 E= -536.634992011 G= -536.510189

Nimag= 0

H	1.917570000	-1.711574000	-0.040570000
C	1.039606000	-1.074407000	-0.020938000
C	-0.230100000	-1.667178000	-0.018974000
H	-0.309703000	-2.749437000	-0.037093000
C	-1.363876000	-0.887241000	0.005632000
H	-2.361594000	-1.313212000	0.007866000
C	-1.262595000	0.557463000	0.029813000
C	0.069428000	1.126195000	0.026761000
C	1.199809000	0.334628000	0.002033000
C	2.520786000	0.975048000	0.000409000
C	3.716806000	0.374429000	-0.020803000
C	4.961552000	1.198998000	-0.018535000
O	4.923647000	2.411599000	0.001878000
H	2.526147000	2.064520000	0.018657000
H	3.831655000	-0.706741000	-0.039921000
C	6.267818000	0.437201000	-0.043203000
H	7.103146000	1.137061000	-0.039285000
H	6.317389000	-0.195586000	-0.936139000
H	6.333533000	-0.224748000	0.827275000
H	0.139514000	2.210020000	0.044905000
O	-2.279578000	1.282997000	0.052311000

33 E= -572.559069875 G= -572.455465

Nimag= 0

H	1.949084000	-1.690550000	-0.037462000
C	1.065916000	-1.060676000	-0.023677000
C	-0.199223000	-1.662728000	-0.020626000
H	-0.270289000	-2.745648000	-0.032160000
C	-1.339267000	-0.891740000	-0.003201000
H	-2.333500000	-1.325842000	-0.000383000
C	-1.250225000	0.553773000	0.012268000
C	0.077422000	1.133317000	0.008461000
C	1.212890000	0.349845000	-0.009108000
C	2.529217000	1.000181000	-0.012093000
C	3.723631000	0.399150000	-0.028544000
C	4.947357000	1.232054000	-0.028647000
O	4.980425000	2.439249000	-0.015447000
H	2.527261000	2.089323000	0.000458000
H	3.863983000	-0.676290000	-0.042124000
O	6.060530000	0.472130000	-0.046845000
H	6.813861000	1.087784000	-0.046147000
H	0.138329000	2.217874000	0.020088000
O	-2.272824000	1.270798000	0.028141000

34 E= -537.647733891 G= -537.509611

Nimag= 0

C	-1.152313000	-0.025249000	-3.732634000
C	-1.569332000	1.113940000	-4.461467000
C	-0.729373000	-1.135813000	-4.432863000
C	-1.557865000	1.136441000	-5.866089000
H	-1.929219000	1.981726000	-3.916869000
C	-0.707149000	-1.160982000	-5.880658000
H	-0.374661000	-2.027459000	-3.924640000
C	-1.141519000	0.035303000	-6.573978000
H	-1.890838000	2.030147000	-6.384471000
H	-1.122346000	0.013078000	-7.658429000
C	-1.176971000	-0.011530000	-2.247909000
C	-1.567540000	-1.148831000	-1.532986000
C	-0.806892000	1.135691000	-1.537781000
H	-0.476569000	2.018160000	-2.079068000
H	-1.880215000	-2.037445000	-2.074117000
C	-0.824783000	1.145064000	-0.146974000
C	-1.585476000	-1.139740000	-0.142430000
H	-0.525943000	2.040482000	0.389122000
H	-1.897694000	-2.028371000	0.397244000
C	-1.214565000	0.007350000	0.554989000
H	-1.229327000	0.014543000	1.640280000
O	-0.322563000	-2.172654000	-6.506351000

35 E= -615.01277989 G= -614.845500

Nimag= 0

H	1.985956000	-1.560933000	-0.651021000
C	1.101971000	-0.995910000	-0.374210000
C	-0.141504000	-1.643355000	-0.363238000
H	-0.189922000	-2.699417000	-0.609641000
C	-1.288144000	-0.947986000	-0.056762000
H	-2.266704000	-1.415950000	-0.043524000
C	-1.225957000	0.466163000	0.253260000
C	0.079830000	1.091013000	0.232893000
C	1.227261000	0.382625000	-0.060195000
C	2.527884000	1.067469000	-0.049877000
C	3.728976000	0.472309000	-0.034454000
H	2.470726000	2.153968000	-0.018208000
H	8.295987000	0.461110000	0.661497000
C	7.423297000	1.039782000	0.375171000

C	6.173416000	0.431674000	0.334171000
H	6.074534000	-0.619769000	0.592248000
C	5.029658000	1.155691000	-0.025123000
C	5.177720000	2.507545000	-0.366893000
H	4.313263000	3.081703000	-0.686305000
C	6.425161000	3.116045000	-0.326187000
H	6.520923000	4.162997000	-0.596968000
C	7.553102000	2.386340000	0.047876000
H	8.527339000	2.864044000	0.075106000
H	3.784241000	-0.614818000	0.007330000
H	0.115647000	2.150808000	0.469090000
O	-2.256286000	1.118660000	0.526586000

36 E= -459.261805816 G= -459.166737

Nimag= 0

H	-1.216344000	-2.305380000	-0.754083000
C	-0.657217000	-1.416943000	-0.478333000
C	0.735390000	-1.383145000	-0.668439000
H	1.243089000	-2.242894000	-1.093080000
C	1.480159000	-0.243139000	-0.322172000
H	2.552610000	-0.249101000	-0.494596000
C	0.872955000	0.880348000	0.199672000
C	-0.571062000	0.867235000	0.450416000
C	-1.297927000	-0.329225000	0.059949000
H	-2.367138000	-0.318347000	0.243960000
O	-1.164069000	1.802758000	1.015150000
C	1.663497000	2.126862000	0.476147000
O	1.172219000	3.221603000	0.330062000
C	3.106614000	1.956687000	0.914973000
H	3.726446000	1.633636000	0.071247000
H	3.198875000	1.205426000	1.704254000
H	3.474878000	2.921021000	1.265385000

37 E= -495.197632372 G= -495.122495

Nimag= 0

H	0.555054000	3.117840000	0.148162000
C	0.186065000	2.251369000	-0.390659000
C	0.683765000	1.965410000	-1.678020000
H	1.432188000	2.614485000	-2.120176000
C	0.224286000	0.850107000	-2.399496000
H	0.606438000	0.629005000	-3.390908000
C	-0.723641000	0.007215000	-1.868534000
C	-1.259971000	0.273499000	-0.538537000
C	-0.759618000	1.434514000	0.172254000
H	-1.173198000	1.612223000	1.159201000
O	-2.125345000	-0.462933000	-0.008346000
C	-1.166219000	-1.170226000	-2.707679000
O	-0.699548000	-1.355200000	-3.803388000
O	-2.081623000	-1.974051000	-2.177550000
H	-2.329536000	-1.647844000	-1.281999000

38 E= -423.354017326 G= -423.234788

Nimag= 0

H	1.847765000	-1.817487000	-0.391899000
C	1.006116000	-1.147738000	-0.243696000
C	-0.265570000	-1.686237000	-0.123034000
H	-0.397591000	-2.762136000	-0.178279000
C	-1.400022000	-0.855081000	0.071874000
H	-2.382685000	-1.307438000	0.162735000
C	-1.249072000	0.498285000	0.143223000
H	-2.086855000	1.171622000	0.291150000
C	0.061397000	1.116148000	0.023024000
C	1.219544000	0.232087000	-0.178205000
O	0.194444000	2.352169000	0.088832000
C	2.515887000	0.872010000	-0.295106000
C	3.692597000	0.250268000	-0.481370000
H	2.491379000	1.956524000	-0.219815000
H	3.730213000	-0.835941000	-0.558505000
C	5.002816000	0.961660000	-0.595267000
H	5.697483000	0.629002000	0.184799000
H	5.482400000	0.746142000	-1.557189000
H	4.875151000	2.043203000	-0.506799000

39 E= -536.639911509 G= -536.514568

Nimag= 0

H	1.933373000	-1.733500000	-0.037347000
C	1.059208000	-1.089759000	-0.022463000
C	-0.203255000	-1.669067000	-0.023111000
H	-0.294678000	-2.750291000	-0.038477000
C	-1.376859000	-0.872004000	-0.004013000
H	-2.348676000	-1.355504000	-0.005077000
C	-1.278367000	0.488665000	0.015533000
H	-2.148149000	1.136863000	0.030573000
C	0.018768000	1.147611000	0.017104000
C	1.218242000	0.296070000	-0.003053000
O	0.105255000	2.387072000	0.034906000
C	2.501546000	0.969245000	-0.001325000
C	3.711161000	0.378936000	-0.017706000
C	4.951772000	1.205683000	-0.013596000
O	4.911111000	2.418317000	0.004536000
H	2.467992000	2.056510000	0.014790000
H	3.832505000	-0.702132000	-0.034320000
C	6.260802000	0.446690000	-0.033247000
H	7.093648000	1.149534000	-0.028227000
H	6.315692000	-0.187299000	-0.925016000
H	6.326966000	-0.213294000	0.838690000

40 E= -572.563997629 G= -572.460015

Nimag= 0

H	1.987205000	-1.661817000	-0.209877000
C	1.089027000	-1.064041000	-0.088846000
C	-0.144636000	-1.703511000	-0.036746000

H	-0.188740000	-2.784714000	-0.117627000
C	-1.346399000	-0.967851000	0.120128000
H	-2.293500000	-1.496707000	0.157278000
C	-1.306257000	0.392815000	0.223127000
H	-2.199990000	0.995639000	0.344230000
C	-0.042986000	1.111729000	0.174992000
C	1.186178000	0.322077000	0.011541000
O	-0.008061000	2.350755000	0.269143000
C	2.436826000	1.056443000	-0.034404000
C	3.660324000	0.520077000	-0.177790000
C	4.844931000	1.405555000	-0.206438000
O	4.832251000	2.608973000	-0.111315000
H	2.356185000	2.137221000	0.056986000
H	3.846554000	-0.544393000	-0.276582000
O	5.984116000	0.697493000	-0.355942000
H	6.709688000	1.345409000	-0.365202000

41 E= -537.650491305 G= -537.511862

Nimag= 0

C	-1.170861000	0.008088000	-3.708154000
C	-1.573949000	1.135222000	-4.414549000
C	-0.735693000	-1.172414000	-4.465779000
C	-1.573884000	1.167997000	-5.809718000
H	-1.928225000	2.003447000	-3.866821000
C	-0.761709000	-1.089593000	-5.915879000
C	-1.162414000	0.046775000	-6.562491000
H	-1.908051000	2.064975000	-6.321189000
H	-0.436526000	-1.977659000	-6.447756000
H	-1.173023000	0.092563000	-7.646895000
C	-1.169387000	0.000471000	-2.235597000
C	-1.503443000	-1.149772000	-1.503702000
C	-0.853222000	1.173814000	-1.533175000
H	-0.555596000	2.061720000	-2.083562000
H	-1.743385000	-2.064483000	-2.030545000
C	-0.874863000	1.202159000	-0.144640000
C	-1.533065000	-1.114564000	-0.114626000
H	-0.614911000	2.116311000	0.379940000
H	-1.802727000	-2.010031000	0.436565000
C	-1.219346000	0.057154000	0.570033000
H	-1.238146000	0.076891000	1.655320000
O	-0.337184000	-2.212958000	-3.906015000

42 E= -615.021167139 G= -614.853408

Nimag= 0

H	2.006210000	-1.691649000	-0.022045000
C	1.123614000	-1.059427000	-0.017389000
C	-0.124418000	-1.653204000	-0.020251000
H	-0.202338000	-2.735699000	-0.027042000
C	-1.312350000	-0.869707000	-0.014431000
H	-2.276926000	-1.367996000	-0.016940000
C	-1.235433000	0.489881000	-0.005811000
H	-2.115655000	1.124126000	-0.001158000
C	0.050597000	1.170810000	-0.002375000
C	1.265546000	0.336151000	-0.008647000
O	0.118799000	2.411858000	0.005580000
C	2.524654000	1.034861000	-0.005231000
C	3.748573000	0.458383000	-0.009809000
H	2.426171000	2.116630000	0.001672000
H	8.358874000	0.398480000	-0.012905000
C	7.459706000	1.006555000	-0.008842000
C	6.211362000	0.395942000	-0.011650000
H	6.139527000	-0.688816000	-0.017898000
C	5.032291000	1.156094000	-0.006515000
C	5.143682000	2.556681000	0.001504000
H	4.249913000	3.172080000	0.005522000
C	6.389825000	3.166077000	0.004289000
H	6.456875000	4.249509000	0.010481000
C	7.553269000	2.395449000	-0.000855000
H	8.525857000	2.877351000	0.001348000
H	3.825354000	-0.628088000	-0.016541000

21an E= -494.649830648 G= -494.589057

Nimag= 0

C	-0.665290000	-1.445450000	-0.501666000
C	0.787928000	-1.440660000	-0.543233000
H	1.264765000	-2.352604000	-0.893461000
C	1.505145000	-0.339133000	-0.158627000
H	2.590240000	-0.306159000	-0.178745000
C	0.853308000	0.832337000	0.292437000
C	-0.559912000	0.861656000	0.345419000
C	-1.301937000	-0.225401000	-0.033148000
H	-2.388577000	-0.227735000	-0.001795000
C	1.679331000	2.071213000	0.724435000
O	0.992467000	3.042030000	1.102607000
H	-1.006747000	1.785768000	0.699421000
O	-1.332139000	-2.445822000	-0.850404000
O	2.914863000	1.924581000	0.632822000

24an E= -572.014069174 G= -571.923834

Nimag= 0

H	1.955841000	-1.724848000	-0.035717000
C	1.066234000	-1.102231000	-0.021496000
C	-0.166918000	-1.678609000	-0.016573000
H	-0.291845000	-2.757531000	-0.026596000
C	-1.389718000	-0.883513000	0.002350000
C	-1.200809000	0.560380000	0.015560000
C	0.044500000	1.114916000	0.010272000
C	1.231160000	0.319620000	-0.008336000
C	2.507558000	0.965985000	-0.012937000
C	3.730884000	0.378066000	-0.029347000
C	5.033025000	1.207923000	-0.032295000
O	4.870124000	2.445571000	-0.018943000

H	2.529680000	2.055796000	-0.002271000
H	3.854365000	-0.704520000	-0.041101000
O	6.065497000	0.508490000	-0.047631000
H	0.165120000	2.195811000	0.020321000
H	-2.099743000	1.169716000	0.029745000
O	-2.517956000	-1.405645000	0.006902000

30an E= -494.644487255 G= -494.583704
Nimag= 0

H	-1.221864000	-2.277677000	-0.818139000
C	-0.622507000	-1.420377000	-0.524915000
C	0.749496000	-1.440110000	-0.539900000
H	1.281580000	-2.335877000	-0.853063000
C	1.476594000	-0.296283000	-0.148290000
H	2.562142000	-0.274848000	-0.147342000
C	0.829289000	0.882818000	0.263677000
C	-0.546203000	0.916583000	0.283665000
C	-1.343045000	-0.228251000	-0.107865000
C	1.683051000	2.116267000	0.685669000
O	1.021658000	3.111500000	1.034535000
O	-2.597945000	-0.212853000	-0.094962000
H	-1.045410000	1.826963000	0.601814000
O	2.916127000	1.931536000	0.615117000

33an E= -572.00006935 G= -571.910621
Nimag= 0

H	1.951279000	-1.661495000	-0.035956000
C	1.059708000	-1.043620000	-0.022789000
C	-0.201396000	-1.658346000	-0.020219000
H	-0.260736000	-2.743264000	-0.031616000
C	-1.347124000	-0.899390000	-0.003434000
H	-2.340003000	-1.337810000	-0.000893000
C	-1.262349000	0.550520000	0.011890000
C	0.060968000	1.134406000	0.008464000
C	1.213029000	0.372378000	-0.008524000
C	2.535791000	1.008001000	-0.011471000
C	3.725931000	0.398417000	-0.029030000
C	5.058927000	1.185656000	-0.030281000
O	4.946266000	2.428245000	-0.013479000
H	2.563246000	2.097266000	0.001795000
H	3.828816000	-0.687792000	-0.043605000
O	6.068412000	0.451186000	-0.048175000
H	0.116496000	2.219320000	0.019938000
O	-2.300035000	1.250795000	0.027270000

37an E= -494.628038319 G= -494.568894
Nimag= 0

H	-1.176304000	-2.286376000	-0.817167000
C	-0.637218000	-1.396024000	-0.501341000
C	0.774367000	-1.349169000	-0.627974000
H	1.302515000	-2.206361000	-1.038754000
C	1.480015000	-0.225802000	-0.222809000
H	2.564191000	-0.198774000	-0.290540000
C	0.841137000	0.906037000	0.288453000
C	-0.607991000	0.872068000	0.469314000
C	-1.306372000	-0.329941000	0.030899000
H	-2.383892000	-0.336518000	0.171443000
O	-1.248624000	1.801539000	1.004445000
C	1.692187000	2.143982000	0.607264000
O	1.376485000	3.161827000	-0.029789000
O	2.621298000	1.921305000	1.409360000

40an E= -572.004652314 G= -571.915597
Nimag= 0

H	1.987431000	-1.625762000	-0.263974000
C	1.078314000	-1.051473000	-0.115007000
C	-0.130895000	-1.701733000	-0.076133000
H	-0.169523000	-2.780652000	-0.195524000
C	-1.342535000	-0.974511000	0.121236000
H	-2.287385000	-1.511855000	0.148481000
C	-1.312592000	0.376559000	0.274355000
H	-2.213185000	0.963610000	0.427258000
C	-0.059647000	1.123588000	0.243358000
C	1.181427000	0.353080000	0.035762000
O	-0.057633000	2.357785000	0.388278000
C	2.421364000	1.065381000	-0.003702000
C	3.651417000	0.518796000	-0.195289000
C	4.951220000	1.345716000	-0.177380000
O	4.800730000	2.581762000	-0.228060000
H	2.382278000	2.144660000	0.121614000
H	3.786446000	-0.553785000	-0.336675000
O	5.977388000	0.637676000	-0.097809000

43a E= -531.743497374 G= -531.683001
Nimag= 0

C	0.576305000	-0.006202000	-2.417981000
C	-0.653871000	-0.342928000	-1.658865000
C	1.738377000	0.330840000	-1.618610000
H	2.656815000	0.582927000	-2.138690000
C	-0.598270000	-0.309124000	-0.210194000
C	1.703822000	0.337961000	-0.250667000
C	0.533153000	0.017814000	0.492962000
H	0.529540000	0.030143000	1.580672000
O	-1.771253000	-0.631658000	0.389682000
H	-2.336779000	-0.800801000	-0.403865000
O	-1.765054000	-0.662222000	-2.178969000
O	2.855470000	0.670268000	0.438293000
H	2.644922000	0.621945000	1.379943000
O	0.593670000	-0.017021000	-3.664359000

43b E= -531.735191473 G= -531.674837
Nimag= 0

C	0.639076000	0.020573000	-2.394414000
C	-0.537985000	-0.325739000	-1.599608000
C	1.792196000	0.361597000	-1.603712000
H	2.712979000	0.632540000	-2.106486000
C	-0.596700000	-0.341121000	-0.130582000
C	1.731085000	0.345248000	-0.226642000
C	0.586618000	0.008807000	0.532110000
H	0.613934000	0.018302000	1.619296000
O	-1.723464000	-0.672668000	0.358144000
O	-1.689966000	-0.666502000	-2.165399000
H	-2.237468000	-0.827461000	-1.341453000
O	2.882534000	0.685496000	0.451504000
H	2.677218000	0.625682000	1.394169000
O	0.599165000	0.007273000	-3.643034000

43c E= -531.740810746 G= -531.681294
Nimag= 0

C	0.706999000	0.057715000	-2.389820000
C	-0.497646000	-0.307096000	-1.638676000
C	1.826398000	0.382900000	-1.637041000
H	2.756345000	0.663738000	-2.119324000
C	-0.572045000	-0.343505000	-0.241051000
C	1.800705000	0.360884000	-0.193430000
C	0.546962000	-0.018353000	0.479760000
H	0.541845000	-0.031059000	1.564238000
O	-1.759860000	-0.702274000	0.354152000
H	-2.373468000	-0.878540000	-0.376452000
O	-1.531813000	-0.606606000	-2.432981000
H	-1.035394000	-0.450398000	-3.323490000
O	2.794383000	0.648857000	0.511715000
O	0.512747000	0.012491000	-3.658058000

44b E= -531.76488232 G= -531.702734
Nimag= 0

C	0.571989000	-0.006120000	-2.316318000
C	-0.646391000	-0.340218000	-1.627536000
C	1.714860000	0.323365000	-1.647573000
H	2.625737000	0.573153000	-2.182037000
C	-0.613416000	-0.313241000	-0.189256000
C	1.751147000	0.351566000	-0.191303000
C	0.516218000	0.012885000	0.503335000
H	0.523915000	0.028240000	1.588460000
O	-1.798803000	-0.639440000	0.401508000
H	-2.376159000	-0.811636000	-0.371268000
O	-1.712256000	-0.647840000	-2.266048000
O	2.793534000	0.652516000	0.432749000
O	0.473289000	-0.050511000	-3.676136000
H	-0.460748000	-0.314959000	-3.809417000

46a E= -531.743256427 G= -531.682493
Nimag= 0

C	0.667166000	-0.011604000	-2.134944000
C	-0.454062000	-0.325342000	-1.323144000
C	1.860030000	0.342039000	-1.553451000
H	2.725841000	0.584405000	-2.164306000
C	-0.334652000	-0.272305000	0.036662000
C	2.059850000	0.419753000	-0.118454000
C	0.879959000	0.088895000	0.719764000
H	0.546390000	-0.061441000	-3.213891000
O	-1.403639000	-0.571202000	0.849640000
H	-0.991352000	-0.439139000	1.735326000
O	-1.650648000	-0.680120000	-1.912879000
H	-2.254465000	-0.845721000	-1.171480000
O	3.158675000	0.745635000	0.389350000
O	0.851916000	0.097746000	1.981892000

46b E= -531.740782882 G= -531.680795
Nimag= 0

C	0.643501000	-0.019075000	-2.177937000
C	-0.445910000	-0.323417000	-1.345331000
C	1.832237000	0.333693000	-1.581605000
H	2.719418000	0.583035000	-2.155553000
C	-0.414810000	-0.294384000	0.120702000
C	1.952182000	0.388382000	-0.133920000
C	0.802065000	0.065818000	0.676195000
H	0.522504000	-0.068955000	-3.256230000
O	-1.539417000	-0.613012000	0.660031000
O	-1.648372000	-0.678034000	-1.781500000
H	-2.085289000	-0.791951000	-0.848638000
O	3.033867000	0.709675000	0.437335000
O	1.006859000	0.143622000	2.030614000
H	1.945752000	0.415975000	2.072645000

46c E= -531.761093291 G= -531.698948
Nimag= 0

C	0.698553000	-0.001065000	-2.215068000
C	-0.488005000	-0.312224000	-1.470386000
C	1.874003000	0.334711000	-1.584976000
H	2.774874000	0.571416000	-2.145075000
C	-0.398013000	-0.258317000	-0.014634000
C	1.967680000	0.388924000	-0.154067000
C	0.763580000	0.073416000	0.608047000
H	0.623087000	-0.043521000	-3.298493000
O	-1.556750000	-0.563954000	0.634141000
H	-2.153874000	-0.743152000	-0.126303000
O	-1.605770000	-0.629849000	-1.973789000
O	3.009619000	0.688772000	0.500289000
O	0.917289000	0.142611000	1.960228000
H	1.863133000	0.403434000	2.026451000

47a E= -531.755561002 G= -531.693579
Nimag= 0

C	0.610985000	-0.012609000	-2.138867000
C	-0.531750000	-0.317575000	-1.462443000
C	1.805177000	0.318540000	-1.415526000
H	2.719847000	0.563049000	-1.947618000
C	-0.610461000	-0.326690000	-0.005602000
C	1.794754000	0.327412000	-0.053142000
C	0.606066000	0.010432000	0.730984000
H	0.611430000	-0.021819000	-3.224877000
O	-1.718653000	-0.623834000	0.523470000
O	-1.709046000	-0.643013000	-2.074138000
H	-2.290764000	-0.795393000	-1.295283000
O	2.886657000	0.631297000	0.709325000
H	2.514401000	0.537173000	1.615213000
O	0.702675000	0.047232000	1.989951000

48a E= -531.729133207 G= -531.670135

Nimag= 0

C	0.686763000	0.020404000	-2.231719000
C	-0.546049000	-0.345349000	-1.573746000
C	1.817459000	0.359095000	-1.533694000
C	-0.533185000	-0.337250000	-0.061902000
C	1.835671000	0.368333000	-0.115457000
C	0.705759000	0.033770000	0.584934000
H	0.724528000	0.042445000	1.670552000
H	0.684106000	0.017079000	-3.321570000
O	-1.554845000	-0.639755000	0.586129000
O	-1.571857000	-0.652913000	-2.214003000
O	3.005676000	0.718564000	0.525118000
H	3.645803000	0.907463000	-0.177864000
O	3.022701000	0.716899000	-2.133011000
H	2.886217000	0.669732000	-3.088207000

48b E= -531.770132216 G= -531.707790

Nimag= 0

C	0.667362000	0.015072000	-2.265873000
C	-0.443358000	-0.314116000	-1.535937000
C	1.876584000	0.377080000	-1.593313000
C	-0.473407000	-0.319494000	-0.072746000
C	1.846387000	0.371643000	-0.130804000
C	0.735373000	0.042404000	0.599498000
H	0.754276000	0.050825000	1.684543000
H	0.649877000	0.007130000	-3.351107000
O	-1.570114000	-0.644661000	0.475730000
O	-1.627430000	-0.668474000	-2.093913000
H	-2.171200000	-0.828749000	-1.284446000
O	3.030574000	0.725983000	0.428188000
H	3.574572000	0.886227000	-0.380556000
O	2.972603000	0.702259000	-2.142896000

48c E= -531.761955031 G= -531.700428

Nimag= 0

C	0.708893000	0.039300000	-2.239882000
C	-0.428278000	-0.306890000	-1.526911000
C	1.829482000	0.379208000	-1.498475000
C	-0.489449000	-0.326666000	-0.050879000
C	1.857951000	0.386526000	-0.021107000
C	0.676411000	0.026941000	0.655950000
H	0.663815000	0.022079000	1.740440000
H	0.721564000	0.044044000	-3.324813000
O	-1.620670000	-0.670931000	0.416554000
O	-1.590398000	-0.659836000	-2.098353000
H	-2.131333000	-0.824695000	-1.272233000
O	2.978264000	0.726170000	0.474600000
O	3.004320000	0.736190000	-2.040881000
H	3.527083000	0.894789000	-1.202405000

49a E= -682.136816906 G= -682.069390

Nimag= 0

C	0.769057000	0.069280000	-2.284932000
C	-0.347378000	-0.278703000	-1.582561000
C	2.019729000	0.449856000	-1.635355000
C	-0.293581000	-0.271583000	-0.153106000
C	2.060697000	0.452954000	-0.170912000
C	0.842662000	0.073430000	0.506065000
O	-1.455381000	-0.633273000	0.508704000
H	-1.243037000	-0.572925000	1.453098000
O	-1.526590000	-0.637536000	-2.195085000
H	-2.145216000	-0.832579000	-1.474585000
O	3.057256000	0.755053000	0.547706000
O	2.990235000	0.753361000	-2.384985000
O	0.894204000	0.080306000	1.880415000
H	1.826456000	0.366657000	2.020603000
O	0.814463000	0.091983000	-3.648670000
H	1.744517000	0.379261000	-3.793472000

49b E= -682.137565621 G= -682.070876

Nimag= 0

C	0.713145000	0.050807000	-2.259046000
C	-0.431521000	-0.305283000	-1.559773000
C	1.949567000	0.426284000	-1.571675000
C	-0.333182000	-0.283995000	-0.180419000
C	1.997792000	0.431991000	-0.170337000
C	0.868475000	0.080537000	0.589361000
O	-1.385846000	-0.612515000	0.598358000
H	-0.963745000	-0.488230000	1.497810000
O	-1.601533000	-0.660700000	-2.196203000
H	-2.226712000	-0.857880000	-1.481687000
O	3.194858000	0.796246000	0.413851000
H	3.755477000	0.973307000	-0.364045000
O	2.942269000	0.735746000	-2.320295000
O	0.717893000	0.026551000	1.849549000
O	0.802827000	0.086947000	-3.597915000

H	1.746232000	0.377793000	-3.704542000

49c E= -682.16052263 G= -682.091343
Nimag= 0

C	0.712606000	0.116727000	-2.307813000
C	-0.422051000	-0.244227000	-1.639285000
C	1.913020000	0.437507000	-1.577525000
C	-0.425286000	-0.310610000	-0.199455000
C	1.909816000	0.371157000	-0.137468000
C	0.775262000	0.010177000	0.531034000
O	-1.484056000	-0.649326000	0.424386000
O	-1.603697000	-0.564422000	-2.244204000
H	-2.176088000	-0.766560000	-1.476086000
O	3.091566000	0.691420000	0.467210000
H	3.663725000	0.893248000	-0.301213000
O	2.972003000	0.776013000	-2.201113000
O	0.676891000	-0.080003000	1.890031000
H	-0.253927000	-0.359550000	2.007587000
O	0.810778000	0.206842000	-3.666875000
H	1.741580000	0.486316000	-3.784704000

Transition states

1 E= -458.147730965 G= -458.065825
Nimag= -2139.66

O	9.260700000	12.992977000	0.769349000
O	8.040723000	12.406342000	0.957669000
H	9.889566000	12.258488000	0.896182000
C	8.977317000	13.329684000	3.583297000
C	9.687323000	12.502222000	4.488119000
C	9.585580000	14.523147000	3.118156000
H	9.203395000	11.592486000	4.827806000
H	9.029277000	15.144210000	2.424601000
C	10.944196000	12.872955000	4.926149000
C	10.854071000	14.873702000	3.556595000
H	11.480267000	12.244915000	5.630790000
H	11.315112000	15.788826000	3.199207000
C	11.533979000	14.056553000	4.459716000
H	12.523470000	14.338140000	4.805600000
O	7.766086000	12.990930000	3.203845000
H	7.737715000	12.801439000	2.057462000

2 E= -611.735486317 G= -611.609046
Nimag= -1899.74

O	-1.435315000	-2.346152000	-0.845194000
H	-1.282850000	-3.166353000	-0.100086000
O	-1.069138000	-3.784304000	0.993734000
O	-0.487863000	-2.807282000	1.744086000
H	-1.221382000	-2.462929000	2.287364000
C	-0.696482000	-1.318482000	-0.485325000
C	-1.305710000	-0.153796000	-0.003609000
C	-0.539176000	0.974059000	0.320375000
C	0.834843000	0.943765000	0.197155000
C	1.502147000	-0.223643000	-0.263886000
C	0.739808000	-1.368889000	-0.613063000
C	1.390498000	-2.534984000	-1.061736000
C	2.763775000	-2.563475000	-1.162279000
C	3.530252000	-1.427937000	-0.822539000
C	2.912892000	-0.281391000	-0.382793000
H	-2.387644000	-0.148011000	0.080245000
H	-1.032147000	1.873984000	0.673907000
H	1.428619000	1.816736000	0.454108000
H	3.497455000	0.595576000	-0.117785000
H	4.611612000	-1.464136000	-0.908956000
H	3.263100000	-3.463869000	-1.505793000
H	0.790572000	-3.400952000	-1.319630000

12 E= -533.343752602 G= -533.259031
Nimag= -1935.55

C	0.812466000	-0.270414000	-1.988084000
C	-0.258881000	-0.591231000	-1.174047000
C	1.892395000	0.449891000	-1.465362000
H	-1.108099000	-1.146152000	-1.557749000
C	-0.274449000	-0.199414000	0.186056000
C	1.900049000	0.842266000	-0.116468000
H	2.753109000	1.400463000	0.253940000
C	0.838039000	0.512473000	0.697696000
H	0.815573000	0.803763000	1.742600000
H	0.819022000	-0.574252000	-3.032117000
O	-1.304675000	-0.461025000	0.965411000
H	-1.405503000	-1.569789000	1.117263000
O	2.966342000	0.798125000	-2.211216000
H	2.847254000	0.484326000	-3.119346000
O	-0.182516000	-2.995517000	0.342092000
O	-1.120510000	-2.781937000	1.309992000
H	0.663076000	-2.956846000	0.826685000

20 E= -610.733478620 G= -610.618331
Nimag= -2181.04

C	-0.711909000	-1.274216000	-0.408941000
C	0.702048000	-1.382210000	-0.339703000
H	1.156309000	-2.340442000	-0.566189000
C	1.466976000	-0.278295000	-0.000561000
H	2.547048000	-0.375572000	0.045524000
C	0.855717000	0.950768000	0.272107000
C	-0.547098000	1.063485000	0.207125000
C	-1.323055000	-0.026183000	-0.115105000
H	-2.404884000	0.032250000	-0.171534000
C	1.640499000	2.174759000	0.636843000
O	1.068264000	3.216808000	0.881434000
C	3.150342000	2.078586000	0.691295000
H	3.554558000	1.772792000	-0.279032000
H	3.465868000	1.335842000	1.431109000

H	3.550495000	3.055182000	0.962163000
H	-0.989850000	2.030119000	0.423658000
O	-1.453887000	-2.293316000	-0.756639000
H	-1.279473000	-3.203296000	-0.034542000
O	-1.189943000	-3.837839000	0.971742000
O	-0.136570000	-3.238275000	1.604717000
H	-0.557499000	-2.773209000	2.351335000

21 E= -646.657496000 G= -646.564492

Nimag= -2202.02

C	-0.703648000	-1.240060000	-0.379646000
C	0.710489000	-1.349557000	-0.298291000
H	1.163409000	-2.314892000	-0.494505000
C	1.477712000	-0.239039000	0.011069000
H	2.557560000	-0.314320000	0.071072000
C	0.857557000	0.993416000	0.237486000
C	-0.541105000	1.116579000	0.160678000
C	-1.316311000	0.016866000	-0.131057000
H	-2.397431000	0.074456000	-0.196399000
C	1.637512000	2.216323000	0.563229000
O	1.151940000	3.302201000	0.771268000
H	-0.983438000	2.090692000	0.341661000
O	-1.446143000	-2.266975000	-0.700144000
H	-1.278700000	-3.163893000	0.051533000
O	2.965825000	1.996076000	0.606914000
H	3.377269000	2.850910000	0.821704000
O	-1.203222000	-3.763755000	1.069874000
O	-0.114265000	-3.192271000	1.668440000
H	-0.500520000	-2.703697000	2.418669000

22 E= -574.811713102 G= -574.674689

Nimag= -2097.51

H	1.961674000	-1.680883000	-0.028999000
C	1.100357000	-1.020823000	-0.016043000
C	-0.160553000	-1.567734000	0.100462000
H	-0.305467000	-2.639686000	0.182306000
C	-1.310435000	-0.734898000	0.107422000
C	-1.131579000	0.664871000	0.002088000
C	0.135126000	1.193278000	-0.128599000
C	1.281310000	0.370599000	-0.131948000
C	2.596257000	1.001314000	-0.257112000
C	3.788489000	0.396297000	-0.178269000
H	2.578818000	2.078738000	-0.423447000
H	3.840713000	-0.676597000	0.001111000
C	5.099094000	1.105213000	-0.316782000
H	5.710435000	0.979282000	0.584040000
H	5.678940000	0.696430000	-1.152162000
H	4.955878000	2.175124000	-0.489095000
H	0.263684000	2.268383000	-0.224057000
O	-2.522494000	-1.235866000	0.176232000
H	-2.656902000	-1.828817000	1.134337000
H	-2.016519000	1.292558000	0.008149000
O	-1.322418000	-2.001579000	2.647389000
O	-2.647180000	-2.132144000	2.340533000
H	-1.283693000	-1.164308000	3.145620000

23 E= -688.099844464 G= -687.955688

Nimag= -2220.94

H	1.689018000	-1.361885000	0.533720000
C	0.874269000	-0.685105000	0.297195000
C	-0.423447000	-1.145763000	0.359197000
H	-0.647854000	-2.170096000	0.636350000
C	-1.509221000	-0.280445000	0.051387000
C	-1.230209000	1.061750000	-0.308184000
H	-2.068465000	1.710584000	-0.538411000
C	0.073653000	1.500214000	-0.380446000
C	1.153714000	0.643920000	-0.075332000
C	2.508080000	1.176048000	-0.159948000
C	3.656836000	0.531281000	0.101002000
C	4.957509000	1.245460000	-0.041386000
O	5.013188000	2.409465000	-0.382985000
H	2.603318000	2.216841000	-0.469172000
H	3.684583000	-0.508563000	0.417308000
C	6.200384000	0.439230000	0.263203000
H	7.085496000	1.060269000	0.127230000
H	6.253713000	-0.433308000	-0.397082000
H	6.163489000	0.064901000	1.292111000
H	0.286203000	2.525406000	-0.671255000
O	-2.747026000	-0.704274000	0.064651000
H	-3.022926000	-1.140325000	1.095899000
O	-3.181279000	-1.228432000	2.300898000
O	-1.905296000	-1.084717000	2.768964000
H	-1.912463000	-0.200038000	3.178831000

24 E= -724.023674460 G= -723.900887

Nimag= -2235.24

H	1.885281000	-1.714898000	0.150887000
C	1.017585000	-1.067767000	0.073307000
C	-0.243671000	-1.622515000	0.120032000
H	-0.387473000	-2.691917000	0.229505000
C	-1.396911000	-0.796569000	0.017095000
C	-1.222894000	0.602981000	-0.124370000
C	0.045012000	1.138107000	-0.185406000
C	1.190707000	0.320648000	-0.081893000
C	2.503413000	0.953061000	-0.139819000
C	3.696681000	0.348705000	-0.040079000
C	4.925048000	1.167476000	-0.121314000
O	4.967156000	2.366078000	-0.270307000
H	2.516839000	2.033855000	-0.276908000
H	3.825068000	-0.719147000	0.099447000
O	6.033542000	0.408942000	-0.006451000
H	6.790292000	1.017043000	-0.069352000

H	0.176355000	2.209712000	-0.308836000
H	-2.111626000	1.220183000	-0.200248000
O	-2.603059000	-1.302433000	0.021098000
H	-2.788846000	-1.925604000	0.978648000
O	-2.868687000	-2.220529000	2.152629000
O	-1.574959000	-2.084583000	2.571924000
H	-1.602368000	-1.290370000	3.136824000

25 E= -689.111214348 G= -688.954713

Nimag= -2135.61

C	-1.161989000	-0.114571000	-3.711630000
C	-1.356731000	1.050480000	-4.470553000
C	-0.935561000	-1.330261000	-4.390280000
C	-1.334802000	1.014393000	-5.852917000
H	-1.561717000	1.987957000	-3.962591000
C	-0.894286000	-1.380892000	-5.767407000
H	-0.756879000	-2.232600000	-3.812908000
C	-1.106905000	-0.208499000	-6.533364000
H	-1.504701000	1.907264000	-6.444783000
H	-0.706970000	-2.307404000	-6.300026000
C	-1.190368000	-0.070554000	-2.234216000
C	-1.723685000	-1.134876000	-1.495025000
C	-0.683607000	1.036894000	-1.541076000
H	-0.240846000	1.858203000	-2.097149000
H	-2.148230000	-1.988011000	-2.016283000
C	-0.708133000	1.078144000	-0.151856000
C	-1.750638000	-1.092129000	-0.105832000
H	-0.301892000	1.940247000	0.367931000
H	-2.177474000	-1.921421000	0.449596000
C	-1.242167000	0.013977000	0.571033000
H	-1.262155000	0.046520000	1.655758000
O	-1.112552000	-0.277550000	-7.844231000
H	-0.295891000	0.394938000	-8.289415000
O	0.795253000	0.917248000	-8.487318000
O	1.054195000	1.495576000	-7.276445000
H	1.729334000	0.911590000	-6.884074000

26 E= -764.304943089 G= -764.145115

Nimag= -2094.15

C	-1.160893000	-0.028848000	-3.678866000
C	-1.445871000	1.135817000	-4.411460000
C	-0.846877000	-1.206774000	-4.389542000
C	-1.426445000	1.134774000	-5.793652000
H	-1.720658000	2.043212000	-3.882158000
C	-0.810008000	-1.221374000	-5.767616000
H	-0.595803000	-2.107199000	-3.836624000
C	-1.112964000	-0.050845000	-6.504806000
H	-1.664061000	2.027022000	-6.362922000
H	-0.556879000	-2.118764000	-6.322531000
C	-1.184931000	-0.022943000	-2.203721000
C	-1.600796000	-1.151668000	-1.479839000
C	-0.792722000	1.109133000	-1.479262000
H	-0.438197000	1.988641000	-2.008589000
C	-0.810582000	1.119321000	-0.090766000
C	-1.627192000	-1.153580000	-0.095070000
H	-0.489326000	2.004748000	0.452889000
C	-1.229373000	-0.013878000	0.607051000
O	-1.123199000	-0.090020000	-7.817626000
H	-0.368975000	0.649455000	-8.247692000
H	-1.939723000	-2.033954000	-2.014819000
H	-1.961210000	-2.020596000	0.464356000
O	-1.271414000	-0.069126000	1.963034000
H	-0.981628000	0.778559000	2.328836000
O	0.684510000	1.265069000	-8.443364000
O	0.929316000	1.799467000	-7.209556000
H	1.645511000	1.240084000	-6.856110000

27 E= -766.477894899 G= -766.292049

Nimag= -2110.81

H	1.991592000	-1.641057000	0.380428000
C	1.106950000	-1.027701000	0.242399000
C	-0.130448000	-1.627984000	0.242768000
H	-0.244311000	-2.698998000	0.374052000
C	-1.309793000	-0.852521000	0.098046000
C	-1.181879000	0.549116000	-0.067505000
C	0.069338000	1.134009000	-0.080659000
C	1.241056000	0.370080000	0.069859000
C	2.529966000	1.050406000	0.040469000
C	3.742069000	0.466239000	0.064396000
H	2.469782000	2.135205000	-0.028536000
H	8.332953000	0.387182000	-0.389593000
C	7.448619000	0.995268000	-0.226790000
C	6.196531000	0.393804000	-0.171983000
H	6.107913000	-0.682709000	-0.294668000
C	5.036158000	1.153389000	0.031660000
C	5.171696000	2.540520000	0.197922000
H	4.295138000	3.152443000	0.386817000
C	6.421344000	3.141859000	0.144037000
H	6.506301000	4.215864000	0.277623000
C	7.565526000	2.373428000	-0.071233000
H	8.541148000	2.847398000	-0.110416000
H	3.806805000	-0.620328000	0.088734000
H	0.157457000	2.209344000	-0.210297000
H	-2.084711000	1.139607000	-0.179262000
O	-2.482122000	-1.439482000	0.151389000
H	-3.079025000	-1.237599000	-0.790269000
O	-2.447684000	-0.274631000	-2.457865000
O	-3.383966000	-1.155740000	-1.995905000
H	-1.834946000	-0.837345000	-2.966293000

28 E= -841.671811742 G= -841.483771

Nimag= -2076.49

H	2.074940000	-1.711634000	-0.138524000
C	1.178089000	-1.106842000	-0.049514000
C	-0.050190000	-1.725708000	-0.065856000
H	-0.144181000	-2.803045000	-0.154458000
C	-1.243364000	-0.967084000	0.046975000
C	-1.140670000	0.442241000	0.150878000
C	0.099837000	1.049107000	0.151071000
C	1.287494000	0.299622000	0.059702000
C	2.563556000	1.001882000	0.073485000
C	3.785388000	0.436196000	0.107020000
H	2.484509000	2.087650000	0.070611000
H	8.389093000	0.408693000	0.480426000
C	7.492334000	1.003581000	0.324027000
C	6.243579000	0.396045000	0.304333000
H	6.175310000	-0.679037000	0.449107000
C	5.069773000	1.134358000	0.108892000
C	5.197651000	2.521901000	-0.081612000
C	6.434011000	3.140560000	-0.064992000
C	7.591063000	2.381888000	0.140727000
H	3.862186000	-0.649077000	0.153083000
H	0.167654000	2.130774000	0.230645000
H	-2.054475000	1.021155000	0.229797000
O	-2.403493000	-1.581938000	0.074536000
H	-3.069484000	-1.220466000	-0.759916000
H	4.314538000	3.128157000	-0.257218000
H	6.534198000	4.210087000	-0.214884000
O	8.776083000	3.044003000	0.145224000
H	9.495811000	2.414475000	0.293988000
O	-2.570832000	0.058945000	-2.256534000
O	-3.469024000	-0.909388000	-1.908780000
H	-1.985171000	-0.389262000	-2.894346000

29 E= -610.731707152 G= -610.616974

Nimag= -2118.35

C	-0.656103000	-1.275796000	-0.432318000
C	0.755111000	-1.397541000	-0.334025000
H	1.201981000	-2.360644000	-0.556992000
C	1.524710000	-0.305271000	0.031392000
H	2.602987000	-0.401671000	0.103369000
C	0.917044000	0.921157000	0.302368000
C	-0.477896000	1.059311000	0.219795000
C	-1.258207000	-0.033448000	-0.131405000
H	-2.336719000	0.065510000	-0.196303000
O	-1.398234000	-2.287989000	-0.814225000
H	-1.307040000	-3.170561000	-0.044866000
O	-1.275362000	-3.769101000	0.986330000
O	-0.274701000	-3.131799000	1.666163000
H	-0.753973000	-2.609090000	2.335644000
H	1.533762000	1.770270000	0.579564000
C	-1.175935000	2.359932000	0.511597000
O	-2.384847000	2.428324000	0.464292000
C	-0.334027000	3.567934000	0.862914000
H	0.253397000	3.381050000	1.767849000
H	0.366840000	3.800110000	0.054521000
H	-0.996406000	4.417155000	1.028249000

36 E= -610.727683097 G= -610.610836

Nimag= -2345.84

C	-0.316052000	-1.389465000	-0.585755000
C	1.102991000	-1.359041000	-0.653230000
H	1.597184000	-2.263771000	-0.990973000
C	1.805256000	-0.220051000	-0.321172000
H	2.887924000	-0.206341000	-0.397109000
C	1.120215000	0.915919000	0.131302000
C	-0.268060000	0.898387000	0.233424000
C	-1.011917000	-0.222352000	-0.130959000
O	-0.917699000	-2.486826000	-0.943428000
H	-1.737993000	-2.813935000	-0.150315000
O	-2.118190000	-3.214055000	0.904886000
O	-1.896835000	-2.146395000	1.737423000
H	-1.195515000	-2.471865000	2.330177000
H	1.670371000	1.810426000	0.404267000
H	-0.781078000	1.785690000	0.590468000
C	-2.514214000	-0.198549000	-0.070967000
O	-3.183406000	-0.959290000	-0.733308000
C	-3.163577000	0.816432000	0.848698000
H	-3.042667000	1.832275000	0.457715000
H	-4.227089000	0.586283000	0.908024000
H	-2.715859000	0.779129000	1.845892000
